# Supplementary material for: An investigation of the modulatory effects of empathic and autistic traits on emotional and facial motor responses during live social interactions
Source: PLoS One. 2024 Jan 9;19(1):e0290765. doi: 10.1371/journal.pone.0290765 (PMC10775989; doi:10.1371/journal.pone.0290765)
Supplement: S1 File — (ZIP) [file pone.0290765.s001.zip › Data_Code/Live_Trait_Rating_EMG_94_2f_robust.html]

Live\_Trait\_Rating\_EMG\_94\_2f\_robust.knit


```
library(tidyverse)
```

```
## ── Attaching core tidyverse packages ──────────────────────── tidyverse 2.0.0 ──
## ✔ dplyr     1.1.2     ✔ readr     2.1.4
## ✔ forcats   1.0.0     ✔ stringr   1.5.0
## ✔ ggplot2   3.4.2     ✔ tibble    3.2.1
## ✔ lubridate 1.9.2     ✔ tidyr     1.3.0
## ✔ purrr     1.0.1     
## ── Conflicts ────────────────────────────────────────── tidyverse_conflicts() ──
## ✖ dplyr::filter() masks stats::filter()
## ✖ dplyr::lag()    masks stats::lag()
## ℹ Use the conflicted package (<http://conflicted.r-lib.org/>) to force all conflicts to become errors
```

```
getCurrentFileLocation <-  function()
{
    this_file <- commandArgs() %>% 
    tibble::enframe(name = NULL) %>%
    tidyr::separate(col=value, into=c("key", "value"), sep="=", fill='right') %>%
    dplyr::filter(key == "--file") %>%
    dplyr::pull(value)
    if (length(this_file)==0)
    {
      this_file <- rstudioapi::getSourceEditorContext()$path
    }
    return(dirname(this_file))
}
# see this post for the source of this function. "https://stackoverflow.com/questions/47044068/get-the-path-of-current-script"
working_directory <- getCurrentFileLocation()
setwd(working_directory)

# LMER
library(lme4)
```

```
## Loading required package: Matrix
## 
## Attaching package: 'Matrix'
## 
## The following objects are masked from 'package:tidyr':
## 
##     expand, pack, unpack
```

```
library(lmerTest)
```

```
## 
## Attaching package: 'lmerTest'
## 
## The following object is masked from 'package:lme4':
## 
##     lmer
## 
## The following object is masked from 'package:stats':
## 
##     step
```

```
library(emmeans)
library(varhandle)
library(parallel)
library(ggpubr)
library(HLMdiag)
```

```
## 
## Attaching package: 'HLMdiag'
## 
## The following object is masked from 'package:stats':
## 
##     covratio
```

```
library(r2glmm)
library(simr)
```

```
## 
## Attaching package: 'simr'
## 
## The following object is masked from 'package:lme4':
## 
##     getData
## 
## The following object is masked from 'package:stringr':
## 
##     fixed
```

```
library(car)
```

```
## Loading required package: carData
## 
## Attaching package: 'car'
## 
## The following object is masked from 'package:dplyr':
## 
##     recode
## 
## The following object is masked from 'package:purrr':
## 
##     some
```

```
library(robustlmm)
```

```
## 
## Attaching package: 'robustlmm'
## 
## The following object is masked from 'package:simr':
## 
##     compare
```

```
library(sjPlot)
```

```
## #refugeeswelcome
```

```
# plotting
library(ggplot2)
library(cowplot)
```

```
## 
## Attaching package: 'cowplot'
## 
## The following objects are masked from 'package:sjPlot':
## 
##     plot_grid, save_plot
## 
## The following object is masked from 'package:ggpubr':
## 
##     get_legend
## 
## The following object is masked from 'package:lubridate':
## 
##     stamp
```

```
library(scales)
```

```
## 
## Attaching package: 'scales'
## 
## The following object is masked from 'package:purrr':
## 
##     discard
## 
## The following object is masked from 'package:readr':
## 
##     col_factor
```

```
library(ggpol)
library(ggeffects)
```

```
## 
## Attaching package: 'ggeffects'
## 
## The following object is masked from 'package:cowplot':
## 
##     get_title
```

```
# import
Rating <- read.delim("Rating_94_traits_meancentered.txt", sep = "\t")
Rating$emotion[Rating$emotion == 1] <- "Positive"
Rating$emotion[Rating$emotion == 2] <- "Negative"
Rating$live[Rating$live == 1] <- "Video" # In the data file the presentation condition is coded as "live".
Rating$live[Rating$live == 2] <- "Live"
Rating$emotion <- factor(Rating$emotion, levels=c('Negative','Positive'),ordered=TRUE)
Rating$live <- factor(Rating$live, levels=c('Video','Live'),ordered=TRUE)
Rating$Type <- factor(Rating$Type)

Rating50idx <- which(Rating$subject<=50)
Rating50 <- Rating[Rating50idx,]

Rating$subject <- factor(Rating$subject)
Rating50$subject <- factor(Rating50$subject)
```

```
## Val: AQ, IRIEC model

val_2f0 <- lmer(VAL~1+emotion*live*IRIEC+emotion*live*AQ+
                  (1+emotion+live|subject), data = Rating, REML = TRUE,
                control=lmerControl(optimizer="bobyqa",
                                    optCtrl=list(maxfun=2e8)))

val_2f1 <- lmer(VAL~1+emotion*live*IRIEC+emotion*live*AQ+
                  (1+emotion*live|subject), data = Rating, REML = TRUE,
                control=lmerControl(optimizer="bobyqa",
                                    optCtrl=list(maxfun=2e8)))
anova(val_2f0, val_2f1) # p < 2.2e-16 ***
```

```
## refitting model(s) with ML (instead of REML)
```

```
## Data: Rating
## Models:
## val_2f0: VAL ~ 1 + emotion * live * IRIEC + emotion * live * AQ + (1 + emotion + live | subject)
## val_2f1: VAL ~ 1 + emotion * live * IRIEC + emotion * live * AQ + (1 + emotion * live | subject)
##         npar    AIC    BIC  logLik deviance  Chisq Df Pr(>Chisq)    
## val_2f0   19 3856.5 3957.5 -1909.2   3818.5                         
## val_2f1   23 3761.3 3883.6 -1857.7   3715.3 103.15  4  < 2.2e-16 ***
## ---
## Signif. codes:  0 '***' 0.001 '**' 0.01 '*' 0.05 '.' 0.1 ' ' 1
```

```
val_2f2 <- lmer(VAL~1+emotion*live*IRIEC+emotion*live*AQ+
                  (1+emotion*live|subject)+(1|Type), data = Rating, REML = TRUE,
                control=lmerControl(optimizer="bobyqa",
                                    optCtrl=list(maxfun=2e8)))
anova(val_2f1, val_2f2) # p = 8.992e-05
```

```
## refitting model(s) with ML (instead of REML)
```

```
## Data: Rating
## Models:
## val_2f1: VAL ~ 1 + emotion * live * IRIEC + emotion * live * AQ + (1 + emotion * live | subject)
## val_2f2: VAL ~ 1 + emotion * live * IRIEC + emotion * live * AQ + (1 + emotion * live | subject) + (1 | Type)
##         npar    AIC    BIC  logLik deviance  Chisq Df Pr(>Chisq)    
## val_2f1   23 3761.3 3883.6 -1857.7   3715.3                         
## val_2f2   24 3748.0 3875.6 -1850.0   3700.0 15.337  1  8.992e-05 ***
## ---
## Signif. codes:  0 '***' 0.001 '**' 0.01 '*' 0.05 '.' 0.1 ' ' 1
```

```
summary(val_2f2, ddf = "Satterthwaite") # Table 1, IRIEC:emotion
```

```
## Linear mixed model fit by REML. t-tests use Satterthwaite's method [
## lmerModLmerTest]
## Formula: VAL ~ 1 + emotion * live * IRIEC + emotion * live * AQ + (1 +  
##     emotion * live | subject) + (1 | Type)
##    Data: Rating
## Control: lmerControl(optimizer = "bobyqa", optCtrl = list(maxfun = 2e+08))
## 
## REML criterion at convergence: 3772.2
## 
## Scaled residuals: 
##     Min      1Q  Median      3Q     Max 
## -4.2633 -0.5422  0.0097  0.5135  6.6449 
## 
## Random effects:
##  Groups   Name             Variance Std.Dev. Corr             
##  subject  (Intercept)      0.1799   0.4241                    
##           emotion.L        0.8980   0.9476    0.13            
##           live.L           0.1002   0.3166   -0.15  0.01      
##           emotion.L:live.L 0.2610   0.5109    0.19  0.14  0.64
##  Type     (Intercept)      0.1195   0.3456                    
##  Residual                  0.4510   0.6716                    
## Number of obs: 1504, groups:  subject, 94; Type, 2
## 
## Fixed effects:
##                          Estimate Std. Error         df t value Pr(>|t|)    
## (Intercept)             5.3044882  0.2488989  0.9898392  21.312   0.0307 *  
## emotion.L               2.2614253  0.1007604 90.9999461  22.444  < 2e-16 ***
## live.L                  0.1269407  0.0408176 90.9999518   3.110   0.0025 ** 
## IRIEC                   0.0037296  0.0094344 90.6507131   0.395   0.6935    
## AQ                      0.0103885  0.0069921 90.3978886   1.486   0.1408    
## emotion.L:live.L        0.3390957  0.0630576 90.9998816   5.378 5.80e-07 ***
## emotion.L:IRIEC         0.0873386  0.0199208 90.9999462   4.384 3.12e-05 ***
## live.L:IRIEC            0.0008896  0.0080698 90.9999517   0.110   0.9125    
## emotion.L:AQ            0.0246393  0.0148776 90.9999465   1.656   0.1011    
## live.L:AQ              -0.0093097  0.0060268 90.9999517  -1.545   0.1259    
## emotion.L:live.L:IRIEC -0.0161697  0.0124668 90.9998816  -1.297   0.1979    
## emotion.L:live.L:AQ    -0.0171592  0.0093106 90.9998816  -1.843   0.0686 .  
## ---
## Signif. codes:  0 '***' 0.001 '**' 0.01 '*' 0.05 '.' 0.1 ' ' 1
## 
## Correlation of Fixed Effects:
##             (Intr) emtn.L live.L IRIEC  AQ     em.L:.L e.L:IR l.L:IR e.L:AQ
## emotion.L    0.022                                                         
## live.L      -0.021  0.005                                                  
## IRIEC        0.002  0.000  0.000                                           
## AQ           0.001  0.000  0.000  0.194                                    
## emtn.L:lv.L  0.028  0.116  0.430  0.000  0.000                             
## emt.L:IRIEC  0.000  0.000  0.000  0.115  0.021  0.000                      
## liv.L:IRIEC  0.000  0.000  0.000 -0.109 -0.020  0.000   0.005              
## emotin.L:AQ  0.000  0.000  0.000  0.021  0.116  0.000   0.178  0.001       
## live.L:AQ    0.000  0.000  0.000 -0.019 -0.110  0.000   0.001  0.178  0.005
## e.L:.L:IRIE  0.000  0.000  0.000  0.148  0.027  0.000   0.116  0.430  0.021
## emt.L:.L:AQ  0.000  0.000  0.000  0.026  0.149  0.000   0.021  0.077  0.116
##             l.L:AQ e.L:.L:I
## emotion.L                  
## live.L                     
## IRIEC                      
## AQ                         
## emtn.L:lv.L                
## emt.L:IRIEC                
## liv.L:IRIEC                
## emotin.L:AQ                
## live.L:AQ                  
## e.L:.L:IRIE  0.077         
## emt.L:.L:AQ  0.430  0.178
```

```
r2beta.val_2f2 <- r2beta(val_2f2, partial = TRUE, method = "nsj")
r2beta.val_2f2
```

```
##                    Effect   Rsq upper.CL lower.CL
## 1                   Model 0.673    0.696    0.652
## 2               emotion.L 0.661    0.683    0.637
## 7         emotion.L:IRIEC 0.069    0.095    0.047
## 6        emotion.L:live.L 0.021    0.038    0.009
## 9            emotion.L:AQ 0.010    0.023    0.003
## 3                  live.L 0.006    0.016    0.001
## 5                      AQ 0.004    0.012    0.000
## 12    emotion.L:live.L:AQ 0.003    0.010    0.000
## 10              live.L:AQ 0.002    0.008    0.000
## 11 emotion.L:live.L:IRIEC 0.001    0.007    0.000
## 4                   IRIEC 0.000    0.005    0.000
## 8            live.L:IRIEC 0.000    0.003    0.000
```

```
conf.val_2f2 <- confint(val_2f2, oldNames = F)
```

```
## Computing profile confidence intervals ...
```

```
## Warning in optwrap(optimizer, par = thopt, fn = mkdevfun(rho, 0L), lower =
## fitted@lower): convergence code 1 from bobyqa: bobyqa -- maximum number of
## function evaluations exceeded

## Warning in optwrap(optimizer, par = thopt, fn = mkdevfun(rho, 0L), lower =
## fitted@lower): convergence code 1 from bobyqa: bobyqa -- maximum number of
## function evaluations exceeded

## Warning in optwrap(optimizer, par = thopt, fn = mkdevfun(rho, 0L), lower =
## fitted@lower): convergence code 1 from bobyqa: bobyqa -- maximum number of
## function evaluations exceeded

## Warning in optwrap(optimizer, par = thopt, fn = mkdevfun(rho, 0L), lower =
## fitted@lower): convergence code 1 from bobyqa: bobyqa -- maximum number of
## function evaluations exceeded

## Warning in optwrap(optimizer, par = thopt, fn = mkdevfun(rho, 0L), lower =
## fitted@lower): convergence code 1 from bobyqa: bobyqa -- maximum number of
## function evaluations exceeded

## Warning in optwrap(optimizer, par = thopt, fn = mkdevfun(rho, 0L), lower =
## fitted@lower): convergence code 1 from bobyqa: bobyqa -- maximum number of
## function evaluations exceeded

## Warning in optwrap(optimizer, par = thopt, fn = mkdevfun(rho, 0L), lower =
## fitted@lower): convergence code 1 from bobyqa: bobyqa -- maximum number of
## function evaluations exceeded

## Warning in optwrap(optimizer, par = thopt, fn = mkdevfun(rho, 0L), lower =
## fitted@lower): convergence code 1 from bobyqa: bobyqa -- maximum number of
## function evaluations exceeded

## Warning in optwrap(optimizer, par = thopt, fn = mkdevfun(rho, 0L), lower =
## fitted@lower): convergence code 1 from bobyqa: bobyqa -- maximum number of
## function evaluations exceeded

## Warning in optwrap(optimizer, par = thopt, fn = mkdevfun(rho, 0L), lower =
## fitted@lower): convergence code 1 from bobyqa: bobyqa -- maximum number of
## function evaluations exceeded

## Warning in optwrap(optimizer, par = thopt, fn = mkdevfun(rho, 0L), lower =
## fitted@lower): convergence code 1 from bobyqa: bobyqa -- maximum number of
## function evaluations exceeded

## Warning in optwrap(optimizer, par = thopt, fn = mkdevfun(rho, 0L), lower =
## fitted@lower): convergence code 1 from bobyqa: bobyqa -- maximum number of
## function evaluations exceeded
```

```
conf.val_2f2
```

```
##                                                 2.5 %       97.5 %
## sd_(Intercept)|subject                    0.355104937 0.4969373908
## cor_emotion.L.(Intercept)|subject        -0.095006735 0.3419441347
## cor_live.L.(Intercept)|subject           -0.422814969 0.1277676606
## cor_emotion.L:live.L.(Intercept)|subject -0.099972254 0.4419416413
## sd_emotion.L|subject                      0.804242521 1.0913323888
## cor_live.L.emotion.L|subject             -0.251889552 0.2644259410
## cor_emotion.L:live.L.emotion.L|subject   -0.107173311 0.3791690776
## sd_live.L|subject                         0.242204130 0.3853345811
## cor_emotion.L:live.L.live.L|subject       0.389055411 0.8579366024
## sd_emotion.L:live.L|subject               0.402566301 0.6132271819
## sd_(Intercept)|Type                       0.095239151 1.0371163085
## sigma                                     0.644792195 0.7002749940
## (Intercept)                               4.705103613 5.9028621951
## emotion.L                                 2.065113277 2.4577372780
## live.L                                    0.047415665 0.2064657587
## IRIEC                                    -0.014468530 0.0226373667
## AQ                                       -0.003148977 0.0242821858
## emotion.L:live.L                          0.216240364 0.4619511249
## emotion.L:IRIEC                           0.048526799 0.1261503599
## live.L:IRIEC                             -0.014832843 0.0166120877
## emotion.L:AQ                             -0.004346721 0.0536252897
## live.L:AQ                                -0.021051804 0.0024323789
## emotion.L:live.L:IRIEC                   -0.040458801 0.0081193405
## emotion.L:live.L:AQ                      -0.035299096 0.0009807719
```

```
val_2f2.em1 <- emmeans(val_2f2, list(pairwise ~ live|emotion),  adjust = "tukey")
```

```
## NOTE: Results may be misleading due to involvement in interactions
```

```
val_2f2.em1
```

```
## $`emmeans of live | emotion`
## emotion = Negative:
##  live  emmean    SE   df lower.CL upper.CL
##  Video   3.79 0.260 1.19     1.50     6.07
##  Live    3.63 0.259 1.17     1.29     5.96
## 
## emotion = Positive:
##  live  emmean    SE   df lower.CL upper.CL
##  Video   6.64 0.264 1.27     4.58     8.71
##  Live    7.16 0.267 1.32     5.21     9.11
## 
## Degrees-of-freedom method: kenward-roger 
## Confidence level used: 0.95 
## 
## $`pairwise differences of live | emotion`
## emotion = Negative:
##  2            estimate     SE df t.ratio p.value
##  Video - Live    0.160 0.0646 91   2.469  0.0154
## 
## emotion = Positive:
##  2            estimate     SE df t.ratio p.value
##  Video - Live   -0.519 0.1022 91  -5.076  <.0001
## 
## Degrees-of-freedom method: kenward-roger
```

```
val_2f2.em11 <- emmeans(val_2f2, list(pairwise ~ emotion|live),  adjust = "tukey")
```

```
## NOTE: Results may be misleading due to involvement in interactions
```

```
val_2f2.em11
```

```
## $`emmeans of emotion | live`
## live = Video:
##  emotion  emmean    SE   df lower.CL upper.CL
##  Negative   3.79 0.260 1.19     1.50     6.07
##  Positive   6.64 0.264 1.27     4.58     8.71
## 
## live = Live:
##  emotion  emmean    SE   df lower.CL upper.CL
##  Negative   3.63 0.259 1.17     1.29     5.96
##  Positive   7.16 0.267 1.32     5.21     9.11
## 
## Degrees-of-freedom method: kenward-roger 
## Confidence level used: 0.95 
## 
## $`pairwise differences of emotion | live`
## live = Video:
##  2                   estimate    SE df t.ratio p.value
##  Negative - Positive    -2.86 0.149 91 -19.187  <.0001
## 
## live = Live:
##  2                   estimate    SE df t.ratio p.value
##  Negative - Positive    -3.54 0.162 91 -21.787  <.0001
## 
## Degrees-of-freedom method: kenward-roger
```

```
val_2f2.em2 <- emtrends(val_2f2, list(pairwise ~ emotion), var = "IRIEC", adjust = "tukey")
```

```
## NOTE: Results may be misleading due to involvement in interactions
```

```
val_2f2.em2
```

```
## $`emmeans of emotion`
##  emotion  IRIEC.trend     SE   df lower.CL upper.CL
##  Negative     -0.0580 0.0160 91.7  -0.0899  -0.0262
##  Positive      0.0655 0.0178 91.6   0.0300   0.1009
## 
## Results are averaged over the levels of: live 
## Degrees-of-freedom method: kenward-roger 
## Confidence level used: 0.95 
## 
## $`pairwise differences of emotion`
##  1                   estimate     SE df t.ratio p.value
##  Negative - Positive   -0.124 0.0282 91  -4.384  <.0001
## 
## Results are averaged over the levels of: live 
## Degrees-of-freedom method: kenward-roger
```

```
val_2f2.predict2 <- ggemmeans(val_2f2, terms = c("IRIEC",  "emotion"))
```

```
## NOTE: Results may be misleading due to involvement in interactions
```

```
val_2f2.IRIECemo <- plot(val_2f2.predict2,  colors = c("steelblue","red"), show.title = F)  +
  labs(x = "Mean-Centered IRIEC", y = "Valence", colour = "Emotion") +
  guides(colour = guide_legend(reverse = T)) +
  theme(title = element_text(size = 8, face = "bold"), axis.title.x = element_text(size = 8, face = "bold"), 
        axis.title.y = element_text(size = 8, face = "bold"), strip.text = element_text(size = 8, face = "bold"),
        legend.position = "none",
        legend.title = element_text(size = 6, face = "bold"), legend.text = element_text(size = 6), 
        axis.text = element_text(size = 6))
val_2f2.IRIECemo　# Fig 4A
```

```
val_2f2.em22 <- emmeans(val_2f2,  ~ emotion*IRIEC, at=list(IRIEC=c(-15.504, -10.336, -5.168, 0, 5.168, 10.336, 15.504), emotion = c("Positive","Negative")), adjust = "sidak")
```

```
## NOTE: Results may be misleading due to involvement in interactions
```

```
val_2f2.em22 # -3SD to +3SD
```

```
##  emotion   IRIEC emmean    SE   df lower.CL upper.CL
##  Positive -15.50   5.89 0.380 5.31    4.012     7.76
##  Negative -15.50   4.61 0.358 4.21    2.518     6.69
##  Positive -10.34   6.23 0.319 2.69    3.140     9.31
##  Negative -10.34   4.31 0.306 2.28    0.420     8.19
##  Positive  -5.17   6.57 0.276 1.52   -2.737    15.87
##  Negative  -5.17   4.01 0.270 1.39   -7.972    15.98
##  Positive   0.00   6.90 0.260 1.20  -13.084    26.89
##  Negative   0.00   3.71 0.257 1.15  -20.251    27.66
##  Positive   5.17   7.24 0.276 1.52   -2.000    16.48
##  Negative   5.17   3.41 0.271 1.40   -8.481    15.29
##  Positive  10.34   7.58 0.319 2.70    4.507    10.65
##  Negative  10.34   3.11 0.306 2.29   -0.757     6.97
##  Positive  15.50   7.92 0.380 5.33    6.045     9.79
##  Negative  15.50   2.81 0.358 4.23    0.723     4.89
## 
## Results are averaged over the levels of: live 
## Degrees-of-freedom method: kenward-roger 
## Confidence level used: 0.95 
## Conf-level adjustment: sidak method for 14 estimates
```

```
contrast(val_2f2.em22, "pairwise", by="IRIEC")
```

```
## IRIEC = -15.50:
##  contrast            estimate    SE df t.ratio p.value
##  Positive - Negative     1.28 0.459 91   2.793  0.0064
## 
## IRIEC = -10.34:
##  contrast            estimate    SE df t.ratio p.value
##  Positive - Negative     1.92 0.324 91   5.927  <.0001
## 
## IRIEC =  -5.17:
##  contrast            estimate    SE df t.ratio p.value
##  Positive - Negative     2.56 0.204 91  12.565  <.0001
## 
## IRIEC =   0.00:
##  contrast            estimate    SE df t.ratio p.value
##  Positive - Negative     3.20 0.142 91  22.444  <.0001
## 
## IRIEC =   5.17:
##  contrast            estimate    SE df t.ratio p.value
##  Positive - Negative     3.84 0.204 91  18.832  <.0001
## 
## IRIEC =  10.34:
##  contrast            estimate    SE df t.ratio p.value
##  Positive - Negative     4.47 0.324 91  13.803  <.0001
## 
## IRIEC =  15.50:
##  contrast            estimate    SE df t.ratio p.value
##  Positive - Negative     5.11 0.459 91  11.129  <.0001
## 
## Results are averaged over the levels of: live 
## Degrees-of-freedom method: kenward-roger
```

```
val_r2f2 <- rlmer(VAL~1+emotion*live*IRIEC+emotion*live*AQ+
                  (1+emotion*live|subject)+(1|Type), data = Rating, REML = TRUE,
                control=lmerControl(optimizer="bobyqa",
                                    optCtrl=list(maxfun=2e8)))
```

```
## Warning in .rlmerInit(lcall, pf, formula, data, method, rho.e, rho.b,
## rho.sigma.e, : Method 'DAStau' does not support blocks of size larger than 2.
## Falling back to method 'DASvar'.
```

```
summary(val_r2f2)
```

```
## Robust linear mixed model fit by DASvar 
## Formula: VAL ~ 1 + emotion * live * IRIEC + emotion * live * AQ + (1 +      emotion * live | subject) + (1 | Type) 
##    Data: Rating 
## Control: lmerControl(optimizer = "bobyqa", optCtrl = list(maxfun = 2e+08)) 
## 
## Scaled residuals: 
##     Min      1Q  Median      3Q     Max 
## -5.9801 -0.6176  0.0134  0.6131  8.9544 
## 
## Random effects:
##  Groups   Name             Variance Std.Dev. Corr             
##  subject  (Intercept)      0.13441  0.3666                    
##           emotion.L        0.86943  0.9324    0.23            
##           live.L           0.08339  0.2888   -0.07  0.03      
##           emotion.L:live.L 0.23672  0.4865    0.19  0.06  0.55
##  Type     (Intercept)      0.14984  0.3871                    
##  Residual                  0.32832  0.5730                    
## Number of obs: 1504, groups: subject, 94; Type, 2
## 
## Fixed effects:
##                         Estimate Std. Error t value
## (Intercept)             5.280028   0.283806  18.604
## emotion.L               2.255842   0.100699  22.402
## live.L                  0.146520   0.037253   3.933
## IRIEC                   0.001928   0.008332   0.231
## AQ                      0.006667   0.006175   1.080
## emotion.L:live.L        0.372523   0.059618   6.248
## emotion.L:IRIEC         0.087164   0.019909   4.378
## live.L:IRIEC            0.002069   0.007365   0.281
## emotion.L:AQ            0.029006   0.014868   1.951
## live.L:AQ              -0.008831   0.005501  -1.605
## emotion.L:live.L:IRIEC -0.006476   0.011787  -0.549
## emotion.L:live.L:AQ    -0.006357   0.008803  -0.722
## 
## Correlation of Fixed Effects:
##             (Intr) emtn.L live.L IRIEC  AQ     em.L:.L e.L:IR l.L:IR e.L:AQ
## emotion.L    0.030                                                         
## live.L      -0.008  0.022                                                  
## IRIEC        0.002  0.000  0.000                                           
## AQ           0.001  0.000  0.000  0.194                                    
## emtn.L:lv.L  0.022  0.051  0.384  0.000  0.000                             
## emt.L:IRIEC  0.000  0.000  0.000  0.202  0.036  0.000                      
## liv.L:IRIEC  0.000  0.000  0.000 -0.052 -0.009  0.000   0.022              
## emotin.L:AQ  0.000  0.000  0.000  0.036  0.204  0.000   0.178  0.004       
## live.L:AQ    0.000  0.000  0.000 -0.009 -0.053  0.000   0.004  0.178  0.022
## e.L:.L:IRIE  0.000  0.000  0.000  0.150  0.027  0.000   0.051  0.384  0.009
## emt.L:.L:AQ  0.000  0.000  0.000  0.027  0.151  0.000   0.009  0.069  0.051
##             l.L:AQ e.L:.L:I
## emotion.L                  
## live.L                     
## IRIEC                      
## AQ                         
## emtn.L:lv.L                
## emt.L:IRIEC                
## liv.L:IRIEC                
## emotin.L:AQ                
## live.L:AQ                  
## e.L:.L:IRIE  0.069         
## emt.L:.L:AQ  0.384  0.178  
## 
## Robustness weights for the residuals: 
##  1259 weights are ~= 1. The remaining 245 ones are summarized as
##    Min. 1st Qu.  Median    Mean 3rd Qu.    Max. 
##   0.150   0.639   0.826   0.773   0.945   0.999 
## 
## Robustness weights for the random effects: 
##  310 weights are ~= 1. The remaining 68 ones are summarized as
##    Min. 1st Qu.  Median    Mean 3rd Qu.    Max. 
##   0.141   0.484   0.631   0.645   0.870   0.965 
## 
## Rho functions used for fitting:
##   Residuals:
##     eff: smoothed Huber (k = 1.345, s = 10) 
##     sig: smoothed Huber, Proposal 2 (k = 1.345, s = 10) 
##   Random Effects, variance component 1 (subject):
##     eff: smoothed Huber (k = 5.91, s = 10) 
##     vcp: smoothed Huber (k = 5.91, s = 10) 
##   Random Effects, variance component 2 (Type):
##     eff: smoothed Huber (k = 1.345, s = 10) 
##     vcp: smoothed Huber, Proposal 2 (k = 1.345, s = 10)
```

```
coefs_val_2f2 <- data.frame(coef(summary(val_2f2, ddf = "Satterthwaite")))
coefs_val_r2f2 <- data.frame(coef(summary(val_r2f2)))
p_values_val_r2f2 <- 2 * pt(abs(coefs_val_r2f2[, "t.value"]), coefs_val_2f2$df, lower = FALSE)
p_values_val_r2f2
```

```
##  [1] 3.514956e-02 8.399038e-39 1.635703e-04 8.175264e-01 2.831651e-01
##  [6] 1.310901e-08 3.189772e-05 7.794346e-01 5.415567e-02 1.118571e-01
## [11] 5.840613e-01 4.720358e-01
```

```
val50_2f0 <- lmer(VAL~1+emotion*live*IRIEC+emotion*live*AQ+
                  (1+emotion+live|subject), data = Rating50, REML = TRUE,
                control=lmerControl(optimizer="bobyqa",
                                    optCtrl=list(maxfun=2e8)))

val50_2f1 <- lmer(VAL~1+emotion*live*IRIEC+emotion*live*AQ+
                  (1+emotion*live|subject), data = Rating50, REML = TRUE,
                control=lmerControl(optimizer="bobyqa",
                                    optCtrl=list(maxfun=2e8)))
anova(val50_2f0, val50_2f1) # p = 6.354e-08 ***
```

```
## refitting model(s) with ML (instead of REML)
```

```
## Data: Rating50
## Models:
## val50_2f0: VAL ~ 1 + emotion * live * IRIEC + emotion * live * AQ + (1 + emotion + live | subject)
## val50_2f1: VAL ~ 1 + emotion * live * IRIEC + emotion * live * AQ + (1 + emotion * live | subject)
##           npar    AIC    BIC  logLik deviance  Chisq Df Pr(>Chisq)    
## val50_2f0   19 2162.1 2251.1 -1062.1   2124.1                         
## val50_2f1   23 2130.9 2238.7 -1042.5   2084.9 39.194  4  6.354e-08 ***
## ---
## Signif. codes:  0 '***' 0.001 '**' 0.01 '*' 0.05 '.' 0.1 ' ' 1
```

```
summary(val50_2f1, ddf = "Satterthwaite") #
```

```
## Linear mixed model fit by REML. t-tests use Satterthwaite's method [
## lmerModLmerTest]
## Formula: VAL ~ 1 + emotion * live * IRIEC + emotion * live * AQ + (1 +  
##     emotion * live | subject)
##    Data: Rating50
## Control: lmerControl(optimizer = "bobyqa", optCtrl = list(maxfun = 2e+08))
## 
## REML criterion at convergence: 2150
## 
## Scaled residuals: 
##     Min      1Q  Median      3Q     Max 
## -3.9200 -0.5481  0.0456  0.5178  4.5139 
## 
## Random effects:
##  Groups   Name             Variance Std.Dev. Corr             
##  subject  (Intercept)      0.2640   0.5138                    
##           emotion.L        1.1604   1.0772    0.17            
##           live.L           0.1241   0.3523   -0.17 -0.03      
##           emotion.L:live.L 0.2212   0.4703    0.17  0.13  0.69
##  Residual                  0.5242   0.7240                    
## Number of obs: 800, groups:  subject, 50
## 
## Fixed effects:
##                         Estimate Std. Error        df t value Pr(>|t|)    
## (Intercept)             5.537210   0.078199 46.999979  70.810  < 2e-16 ***
## emotion.L               2.256249   0.158948 46.999978  14.195  < 2e-16 ***
## live.L                  0.037378   0.062518 47.000006   0.598 0.552788    
## IRIEC                   0.003420   0.015505 46.999979   0.221 0.826378    
## AQ                      0.017900   0.011917 46.999979   1.502 0.139758    
## emotion.L:live.L        0.136197   0.085202 47.000047   1.599 0.116629    
## emotion.L:IRIEC         0.121484   0.031516 46.999978   3.855 0.000351 ***
## live.L:IRIEC            0.001008   0.012396 47.000006   0.081 0.935510    
## emotion.L:AQ            0.017875   0.024222 46.999978   0.738 0.464203    
## live.L:AQ              -0.017284   0.009527 47.000006  -1.814 0.076028 .  
## emotion.L:live.L:IRIEC -0.007528   0.016894 47.000048  -0.446 0.657950    
## emotion.L:live.L:AQ    -0.027203   0.012984 47.000048  -2.095 0.041573 *  
## ---
## Signif. codes:  0 '***' 0.001 '**' 0.01 '*' 0.05 '.' 0.1 ' ' 1
## 
## Correlation of Fixed Effects:
##             (Intr) emtn.L live.L IRIEC  AQ     em.L:.L e.L:IR l.L:IR e.L:AQ
## emotion.L    0.158                                                         
## live.L      -0.128 -0.022                                                  
## IRIEC       -0.148 -0.023  0.019                                           
## AQ          -0.084 -0.013  0.011 -0.016                                    
## emtn.L:lv.L  0.130  0.099  0.439 -0.019 -0.011                             
## emt.L:IRIEC -0.023 -0.148  0.003  0.158 -0.003 -0.015                      
## liv.L:IRIEC  0.019  0.003 -0.148 -0.128  0.002 -0.065  -0.022              
## emotin.L:AQ -0.013 -0.084  0.002 -0.003  0.158 -0.008  -0.016  0.000       
## live.L:AQ    0.011  0.002 -0.084  0.002 -0.128 -0.037   0.000 -0.016 -0.022
## e.L:.L:IRIE -0.019 -0.015 -0.065  0.130 -0.002 -0.148   0.099  0.439 -0.002
## emt.L:.L:AQ -0.011 -0.008 -0.037 -0.002  0.130 -0.084  -0.002 -0.007  0.099
##             l.L:AQ e.L:.L:I
## emotion.L                  
## live.L                     
## IRIEC                      
## AQ                         
## emtn.L:lv.L                
## emt.L:IRIEC                
## liv.L:IRIEC                
## emotin.L:AQ                
## live.L:AQ                  
## e.L:.L:IRIE -0.007         
## emt.L:.L:AQ  0.439 -0.016
```

```
val50_r2f1 <- rlmer(VAL~1+emotion*live*IRIEC+emotion*live*AQ+
                  (1+emotion*live|subject), data = Rating50, REML = TRUE,
                control=lmerControl(optimizer="bobyqa",
                                    optCtrl=list(maxfun=2e8)))
```

```
## Warning in .rlmerInit(lcall, pf, formula, data, method, rho.e, rho.b,
## rho.sigma.e, : Method 'DAStau' does not support blocks of size larger than 2.
## Falling back to method 'DASvar'.
```

```
summary(val50_r2f1)
```

```
## Robust linear mixed model fit by DASvar 
## Formula: VAL ~ 1 + emotion * live * IRIEC + emotion * live * AQ + (1 +      emotion * live | subject) 
##    Data: Rating50 
## Control: lmerControl(optimizer = "bobyqa", optCtrl = list(maxfun = 2e+08)) 
## 
## Scaled residuals: 
##     Min      1Q  Median      3Q     Max 
## -5.3909 -0.6427  0.0131  0.6539  7.2871 
## 
## Random effects:
##  Groups   Name             Variance Std.Dev. Corr             
##  subject  (Intercept)      0.18223  0.4269                    
##           emotion.L        1.18900  1.0904    0.27            
##           live.L           0.06715  0.2591   -0.04  0.07      
##           emotion.L:live.L 0.15307  0.3912    0.06 -0.05  0.73
##  Residual                  0.39128  0.6255                    
## Number of obs: 800, groups: subject, 50
## 
## Fixed effects:
##                         Estimate Std. Error t value
## (Intercept)             5.495314   0.066792   82.28
## emotion.L               2.295080   0.163427   14.04
## live.L                  0.077765   0.050089    1.55
## IRIEC                   0.007255   0.013244    0.55
## AQ                      0.012089   0.010178    1.19
## emotion.L:live.L        0.140497   0.073638    1.91
## emotion.L:IRIEC         0.125410   0.032405    3.87
## live.L:IRIEC           -0.000928   0.009932   -0.09
## emotion.L:AQ            0.031070   0.024904    1.25
## live.L:AQ              -0.017462   0.007633   -2.29
## emotion.L:live.L:IRIEC  0.007416   0.014601    0.51
## emotion.L:live.L:AQ    -0.016900   0.011222   -1.51
## 
## Correlation of Fixed Effects:
##             (Intr) emtn.L live.L IRIEC  AQ     em.L:.L e.L:IR l.L:IR e.L:AQ
## emotion.L    0.247                                                         
## live.L      -0.028  0.051                                                  
## IRIEC       -0.148 -0.037  0.004                                           
## AQ          -0.084 -0.021  0.002 -0.016                                    
## emtn.L:lv.L  0.045 -0.038  0.434 -0.007 -0.004                             
## emt.L:IRIEC -0.037 -0.148 -0.008  0.247 -0.004  0.006                      
## liv.L:IRIEC  0.004 -0.008 -0.148 -0.028  0.000 -0.064   0.051              
## emotin.L:AQ -0.021 -0.084 -0.004 -0.004  0.247  0.003  -0.016 -0.001       
## live.L:AQ    0.002 -0.004 -0.084  0.000 -0.028 -0.037  -0.001 -0.016  0.051
## e.L:.L:IRIE -0.007  0.006 -0.064  0.045 -0.001 -0.148  -0.038  0.434  0.001
## emt.L:.L:AQ -0.004  0.003 -0.037 -0.001  0.045 -0.084   0.001 -0.007 -0.038
##             l.L:AQ e.L:.L:I
## emotion.L                  
## live.L                     
## IRIEC                      
## AQ                         
## emtn.L:lv.L                
## emt.L:IRIEC                
## liv.L:IRIEC                
## emotin.L:AQ                
## live.L:AQ                  
## e.L:.L:IRIE -0.007         
## emt.L:.L:AQ  0.434 -0.016  
## 
## Robustness weights for the residuals: 
##  664 weights are ~= 1. The remaining 136 ones are summarized as
##    Min. 1st Qu.  Median    Mean 3rd Qu.    Max. 
##   0.185   0.634   0.813   0.770   0.939   0.998 
## 
## Robustness weights for the random effects: 
##  184 weights are ~= 1. The remaining 16 ones are summarized as
##    Min. 1st Qu.  Median    Mean 3rd Qu.    Max. 
##   0.152   0.190   0.257   0.360   0.426   0.773 
## 
## Rho functions used for fitting:
##   Residuals:
##     eff: smoothed Huber (k = 1.345, s = 10) 
##     sig: smoothed Huber, Proposal 2 (k = 1.345, s = 10) 
##   Random Effects, variance component 1 (subject):
##     eff: smoothed Huber (k = 5.91, s = 10) 
##     vcp: smoothed Huber (k = 5.91, s = 10)
```

```
coefs_val50_2f1 <- data.frame(coef(summary(val50_2f1, ddf = "Satterthwaite")))
coefs_val50_r2f1 <- data.frame(coef(summary(val50_r2f1)))
p_values_val50_r2f1 <- 2 * pt(abs(coefs_val50_r2f1[, "t.value"]), coefs_val50_2f1$df, lower = FALSE)
p_values_val50_r2f1
```

```
##  [1] 1.866546e-52 1.947375e-18 1.272447e-01 5.864325e-01 2.409040e-01
##  [6] 6.251762e-02 3.343850e-04 9.259533e-01 2.183764e-01 2.669701e-02
## [11] 6.138848e-01 1.387567e-01
```

```
# AQ:emotion:live is not significant -> model diagnostics
```

```
resid1_val50_2f1 <- hlm_resid(val50_2f1, level = 1, type = "LS", standardize = TRUE)
cooksd1_val50_2f1 <- cooks.distance(val50_2f1)
Q3.cooksd1_val50_2f1 <- quantile(cooksd1_val50_2f1, 0.75, names = TRUE)   
cooksd1.th.val50_2f1 <- Q3.cooksd1_val50_2f1 + 1.5*IQR(cooksd1_val50_2f1)
cooksd2_val50_2f1 <- cooks.distance(val50_2f1, level = "subject")
dotplot_diag(x = cooksd2_val50_2f1, cutoff = "internal",
             name = "cooks.distance") + ylab("Cook's distance") + xlab("trial")
```

```
cooksd2_val50_2f1
```

```
##  [1] 0.002124111 0.028251847 0.018418400 0.006546890 0.034596673 0.007290374
##  [7] 0.002306515 0.007870407 0.012179115 0.007564220 0.044507721 0.030034415
## [13] 0.097173742 0.005411863 0.043756621 0.013320027 0.202975575 0.021056821
## [19] 0.018831867 0.006264062 0.005456420 0.001869294 0.004637222 0.010847919
## [25] 0.001353473 0.001120422 0.005975152 0.045556021 0.011716339 0.015154434
## [31] 0.135426972 0.010312496 0.038781289 0.029473278 0.007407081 0.006144803
## [37] 0.074360970 0.009183873 0.004319424 0.006125276 0.002765791 0.025474280
## [43] 0.006856776 0.018806818 0.017570663 0.024562218 0.002473461 0.015052449
## [49] 0.044655506 0.024494898
```

```
# 13, 17, 31 are highly influential subjects
```

```
Rating50_subject <- unique(Rating50$subject)
Rating50_subject
```

```
##  [1] 1  2  3  4  5  6  7  8  9  10 11 12 13 14 15 16 17 18 19 20 21 22 23 24 25
## [26] 26 27 28 29 30 31 32 33 34 35 36 37 38 39 40 41 42 43 44 45 46 47 48 49 50
## 50 Levels: 1 2 3 4 5 6 7 8 9 10 11 12 13 14 15 16 17 18 19 20 21 22 23 ... 50
```

```
OLindex.val50_2f1 = which(cooksd1_val50_2f1 > cooksd1.th.val50_2f1 | abs(resid1_val50_2f1$.std.resid) > 3
                       | Rating50$subject == Rating50_subject[13]| Rating50$subject == Rating50_subject[17] 
                       | Rating50$subject == Rating50_subject[31])

tbl_val50_2f1 <- Rating50[-OLindex.val50_2f1,]
val50_2f11 <- lmer(VAL~1+emotion*live*IRIEC+emotion*live*AQ+
                  (1+emotion*live|subject), data = tbl_val50_2f1, REML = TRUE,
                control=lmerControl(optimizer="bobyqa",
                                    optCtrl=list(maxfun=2e8)))
summary(val50_2f11)
```

```
## Linear mixed model fit by REML. t-tests use Satterthwaite's method [
## lmerModLmerTest]
## Formula: VAL ~ 1 + emotion * live * IRIEC + emotion * live * AQ + (1 +  
##     emotion * live | subject)
##    Data: tbl_val50_2f1
## Control: lmerControl(optimizer = "bobyqa", optCtrl = list(maxfun = 2e+08))
## 
## REML criterion at convergence: 1535.5
## 
## Scaled residuals: 
##      Min       1Q   Median       3Q      Max 
## -2.47985 -0.64648  0.06009  0.58834  2.34383 
## 
## Random effects:
##  Groups   Name             Variance Std.Dev. Corr             
##  subject  (Intercept)      0.14714  0.3836                    
##           emotion.L        1.20310  1.0969    0.20            
##           live.L           0.09504  0.3083   -0.16 -0.01      
##           emotion.L:live.L 0.11229  0.3351   -0.01 -0.15  0.74
##  Residual                  0.30555  0.5528                    
## Number of obs: 692, groups:  subject, 47
## 
## Fixed effects:
##                         Estimate Std. Error        df t value Pr(>|t|)    
## (Intercept)             5.461592   0.061188 42.656194  89.260  < 2e-16 ***
## emotion.L               2.277540   0.165673 43.709598  13.747  < 2e-16 ***
## live.L                  0.062168   0.055153 42.775779   1.127 0.265943    
## IRIEC                   0.021861   0.012458 44.178129   1.755 0.086239 .  
## AQ                      0.002187   0.010570 46.310361   0.207 0.836982    
## emotion.L:live.L        0.133901   0.066130 43.344027   2.025 0.049074 *  
## emotion.L:IRIEC         0.133309   0.033385 44.324308   3.993 0.000242 ***
## live.L:IRIEC            0.004378   0.011276 45.615498   0.388 0.699633    
## emotion.L:AQ            0.039470   0.027288 46.665465   1.446 0.154742    
## live.L:AQ              -0.014748   0.009205 47.029542  -1.602 0.115830    
## emotion.L:live.L:IRIEC  0.004968   0.013622 47.361269   0.365 0.716954    
## emotion.L:live.L:AQ    -0.013538   0.011141 48.398321  -1.215 0.230229    
## ---
## Signif. codes:  0 '***' 0.001 '**' 0.01 '*' 0.05 '.' 0.1 ' ' 1
## 
## Correlation of Fixed Effects:
##             (Intr) emtn.L live.L IRIEC  AQ     em.L:.L e.L:IR l.L:IR e.L:AQ
## emotion.L    0.176                                                         
## live.L      -0.120 -0.012                                                  
## IRIEC       -0.159 -0.042  0.024                                           
## AQ          -0.052  0.019  0.007 -0.039                                    
## emtn.L:lv.L -0.007 -0.109  0.440  0.003 -0.008                             
## emt.L:IRIEC -0.042 -0.170  0.003  0.164  0.033  0.022                      
## liv.L:IRIEC  0.024  0.003 -0.176 -0.113  0.005 -0.092  -0.018              
## emotin.L:AQ  0.019 -0.026 -0.005  0.034  0.092  0.004   0.002 -0.008       
## live.L:AQ    0.008 -0.005 -0.030  0.005 -0.114  0.012  -0.008  0.002 -0.001
## e.L:.L:IRIE  0.002  0.021 -0.091 -0.022 -0.011 -0.179  -0.103  0.414  0.001
## emt.L:.L:AQ -0.008  0.004  0.012 -0.012  0.006 -0.035   0.001  0.016 -0.102
##             l.L:AQ e.L:.L:I
## emotion.L                  
## live.L                     
## IRIEC                      
## AQ                         
## emtn.L:lv.L                
## emt.L:IRIEC                
## liv.L:IRIEC                
## emotin.L:AQ                
## live.L:AQ                  
## e.L:.L:IRIE  0.016         
## emt.L:.L:AQ  0.349 -0.002
```

```
r2beta.val50_2f11 <- r2beta(val50_2f11, partial = TRUE, method = "nsj")
r2beta.val50_2f11
```

```
##                    Effect   Rsq upper.CL lower.CL
## 1                   Model 0.735    0.762    0.708
## 2               emotion.L 0.686    0.717    0.654
## 7         emotion.L:IRIEC 0.148    0.197    0.103
## 9            emotion.L:AQ 0.021    0.047    0.005
## 4                   IRIEC 0.009    0.029    0.001
## 6        emotion.L:live.L 0.004    0.018    0.000
## 10              live.L:AQ 0.003    0.017    0.000
## 3                  live.L 0.002    0.013    0.000
## 12    emotion.L:live.L:AQ 0.001    0.012    0.000
## 8            live.L:IRIEC 0.000    0.008    0.000
## 5                      AQ 0.000    0.008    0.000
## 11 emotion.L:live.L:IRIEC 0.000    0.008    0.000
```

```
conf.val50_2f11 <- confint(val50_2f11, oldNames = F)
```

```
## Computing profile confidence intervals ...
```

```
conf.val50_2f11
```

```
##                                                 2.5 %      97.5 %
## sd_(Intercept)|subject                    0.292534312 0.472896695
## cor_emotion.L.(Intercept)|subject        -0.110310319 0.481693812
## cor_live.L.(Intercept)|subject           -0.498780024 0.221713519
## cor_emotion.L:live.L.(Intercept)|subject -0.426349172 0.405143111
## sd_emotion.L|subject                      0.868264902 1.326994296
## cor_live.L.emotion.L|subject             -0.356936254 0.332577976
## cor_emotion.L:live.L.emotion.L|subject   -0.529639855 0.241337417
## sd_live.L|subject                         0.210259163 0.396314957
## cor_emotion.L:live.L.live.L|subject       0.345029091 0.883201811
## sd_emotion.L:live.L|subject               0.199095937 0.448641745
## sigma                                     0.520399794 0.588791600
## (Intercept)                               5.342680110 5.579637313
## emotion.L                                 1.956439265 2.598062484
## live.L                                   -0.044466295 0.168938022
## IRIEC                                    -0.002278482 0.045985846
## AQ                                       -0.018120147 0.022858908
## emotion.L:live.L                          0.006281297 0.262353235
## emotion.L:IRIEC                           0.068440408 0.197724542
## live.L:IRIEC                             -0.017392919 0.026208190
## emotion.L:AQ                             -0.013163448 0.092464384
## live.L:AQ                                -0.032576586 0.002993090
## emotion.L:live.L:IRIEC                   -0.021261554 0.031455870
## emotion.L:live.L:AQ                      -0.035142037 0.007921116
```

```
## Aro: AQ, IRIEC model

aro_2f0 <- lmer(ARO~1+emotion*live*IRIEC+emotion*live*AQ+
                  (1+emotion+live|subject), data = Rating, REML = TRUE,
                control=lmerControl(optimizer="bobyqa",
                                    optCtrl=list(maxfun=2e8)))
aro_2f3 <- lmer(ARO~1+emotion*live*IRIEC+emotion*live*AQ+
                  (1+emotion+live|subject)+(1|Type), data = Rating, REML = TRUE,
                control=lmerControl(optimizer="bobyqa",
                                    optCtrl=list(maxfun=2e8)))
```

```
## boundary (singular) fit: see help('isSingular')
```

```
#Singular
anova(aro_2f0, aro_2f3) # p = 1
```

```
## refitting model(s) with ML (instead of REML)
```

```
## Data: Rating
## Models:
## aro_2f0: ARO ~ 1 + emotion * live * IRIEC + emotion * live * AQ + (1 + emotion + live | subject)
## aro_2f3: ARO ~ 1 + emotion * live * IRIEC + emotion * live * AQ + (1 + emotion + live | subject) + (1 | Type)
##         npar    AIC    BIC  logLik deviance Chisq Df Pr(>Chisq)
## aro_2f0   19 4426.4 4527.4 -2194.2   4388.4                    
## aro_2f3   20 4428.4 4534.7 -2194.2   4388.4     0  1          1
```

```
aro_2f1 <- lmer(ARO~1+emotion*live*IRIEC+emotion*live*AQ+
                  (1+emotion*live|subject), data = Rating, REML = TRUE,
                control=lmerControl(optimizer="bobyqa",
                                    optCtrl=list(maxfun=2e8)))
anova(aro_2f0, aro_2f1) # p = 3.135e-05 ***
```

```
## refitting model(s) with ML (instead of REML)
```

```
## Data: Rating
## Models:
## aro_2f0: ARO ~ 1 + emotion * live * IRIEC + emotion * live * AQ + (1 + emotion + live | subject)
## aro_2f1: ARO ~ 1 + emotion * live * IRIEC + emotion * live * AQ + (1 + emotion * live | subject)
##         npar    AIC    BIC  logLik deviance Chisq Df Pr(>Chisq)    
## aro_2f0   19 4426.4 4527.4 -2194.2   4388.4                        
## aro_2f1   23 4408.4 4530.6 -2181.2   4362.4 26.02  4  3.135e-05 ***
## ---
## Signif. codes:  0 '***' 0.001 '**' 0.01 '*' 0.05 '.' 0.1 ' ' 1
```

```
aro_2f2 <- lmer(ARO~1+emotion*live*IRIEC+emotion*live*AQ+
                  (1+emotion*live|subject)+(1|Type), data = Rating, REML = TRUE,
                control=lmerControl(optimizer="bobyqa",
                                    optCtrl=list(maxfun=2e8)))
```

```
## boundary (singular) fit: see help('isSingular')
```

```
# Singular
anova(aro_2f1, aro_2f2) # p = 1, no need to add Type as a random factor
```

```
## refitting model(s) with ML (instead of REML)
```

```
## Data: Rating
## Models:
## aro_2f1: ARO ~ 1 + emotion * live * IRIEC + emotion * live * AQ + (1 + emotion * live | subject)
## aro_2f2: ARO ~ 1 + emotion * live * IRIEC + emotion * live * AQ + (1 + emotion * live | subject) + (1 | Type)
##         npar    AIC    BIC  logLik deviance Chisq Df Pr(>Chisq)
## aro_2f1   23 4408.4 4530.6 -2181.2   4362.4                    
## aro_2f2   24 4410.4 4538.0 -2181.2   4362.4     0  1          1
```

```
summary(aro_2f1, ddf = "Satterthwaite") # Table 3
```

```
## Linear mixed model fit by REML. t-tests use Satterthwaite's method [
## lmerModLmerTest]
## Formula: ARO ~ 1 + emotion * live * IRIEC + emotion * live * AQ + (1 +  
##     emotion * live | subject)
##    Data: Rating
## Control: lmerControl(optimizer = "bobyqa", optCtrl = list(maxfun = 2e+08))
## 
## REML criterion at convergence: 4431.7
## 
## Scaled residuals: 
##     Min      1Q  Median      3Q     Max 
## -4.3612 -0.4621  0.0225  0.5220  3.3136 
## 
## Random effects:
##  Groups   Name             Variance Std.Dev. Corr             
##  subject  (Intercept)      0.4218   0.6495                    
##           emotion.L        1.3720   1.1713   -0.17            
##           live.L           0.2593   0.5093   -0.04 -0.23      
##           emotion.L:live.L 0.1453   0.3812    0.05  0.21  0.39
##  Residual                  0.6915   0.8316                    
## Number of obs: 1504, groups:  subject, 94
## 
## Fixed effects:
##                         Estimate Std. Error        df t value Pr(>|t|)    
## (Intercept)             5.526596   0.070334 90.999995  78.576  < 2e-16 ***
## emotion.L               1.207347   0.124561 90.999961   9.693 1.12e-15 ***
## live.L                  0.293374   0.060651 91.000000   4.837 5.34e-06 ***
## IRIEC                   0.032363   0.013905 90.999994   2.327  0.02216 *  
## AQ                      0.014539   0.010385 90.999994   1.400  0.16493    
## emotion.L:live.L        0.154255   0.058181 91.000016   2.651  0.00946 ** 
## emotion.L:IRIEC         0.077654   0.024626 90.999964   3.153  0.00219 ** 
## live.L:IRIEC            0.003673   0.011991 90.999999   0.306  0.76009    
## emotion.L:AQ            0.012576   0.018392 90.999964   0.684  0.49585    
## live.L:AQ               0.002615   0.008955 90.999999   0.292  0.77095    
## emotion.L:live.L:IRIEC  0.002738   0.011503 91.000016   0.238  0.81241    
## emotion.L:live.L:AQ    -0.003628   0.008591 91.000016  -0.422  0.67375    
## ---
## Signif. codes:  0 '***' 0.001 '**' 0.01 '*' 0.05 '.' 0.1 ' ' 1
## 
## Correlation of Fixed Effects:
##             (Intr) emtn.L live.L IRIEC  AQ     em.L:.L e.L:IR l.L:IR e.L:AQ
## emotion.L   -0.160                                                         
## live.L      -0.029 -0.194                                                  
## IRIEC        0.000  0.000  0.000                                           
## AQ           0.000  0.000  0.000  0.178                                    
## emtn.L:lv.L  0.035  0.135  0.228  0.000  0.000                             
## emt.L:IRIEC  0.000  0.000  0.000 -0.160 -0.029  0.000                      
## liv.L:IRIEC  0.000  0.000  0.000 -0.029 -0.005  0.000  -0.194              
## emotin.L:AQ  0.000  0.000  0.000 -0.029 -0.160  0.000   0.178 -0.035       
## live.L:AQ    0.000  0.000  0.000 -0.005 -0.029  0.000  -0.035  0.178 -0.194
## e.L:.L:IRIE  0.000  0.000  0.000  0.035  0.006  0.000   0.135  0.228  0.024
## emt.L:.L:AQ  0.000  0.000  0.000  0.006  0.035  0.000   0.024  0.041  0.135
##             l.L:AQ e.L:.L:I
## emotion.L                  
## live.L                     
## IRIEC                      
## AQ                         
## emtn.L:lv.L                
## emt.L:IRIEC                
## liv.L:IRIEC                
## emotin.L:AQ                
## live.L:AQ                  
## e.L:.L:IRIE  0.041         
## emt.L:.L:AQ  0.228  0.178
```

```
r2beta.aro_2f1 <- r2beta(aro_2f1, partial = TRUE, method = "nsj")
r2beta.aro_2f1
```

```
##                    Effect   Rsq upper.CL lower.CL
## 1                   Model 0.311    0.350    0.279
## 2               emotion.L 0.271    0.307    0.235
## 7         emotion.L:IRIEC 0.038    0.059    0.021
## 3                  live.L 0.021    0.038    0.009
## 4                   IRIEC 0.013    0.027    0.004
## 5                      AQ 0.005    0.014    0.000
## 6        emotion.L:live.L 0.003    0.011    0.000
## 9            emotion.L:AQ 0.002    0.009    0.000
## 8            live.L:IRIEC 0.000    0.004    0.000
## 10              live.L:AQ 0.000    0.004    0.000
## 12    emotion.L:live.L:AQ 0.000    0.004    0.000
## 11 emotion.L:live.L:IRIEC 0.000    0.003    0.000
```

```
conf.aro_2f1 <- confint(aro_2f1, oldNames = F)
```

```
## Computing profile confidence intervals ...
```

```
conf.aro_2f1
```

```
##                                                 2.5 %      97.5 %
## sd_(Intercept)|subject                    0.547145222 0.751394626
## cor_emotion.L.(Intercept)|subject        -0.376661457 0.044920397
## cor_live.L.(Intercept)|subject           -0.275856060 0.208716058
## cor_emotion.L:live.L.(Intercept)|subject -0.262299434 0.365448704
## sd_emotion.L|subject                      0.994071179 1.349011768
## cor_live.L.emotion.L|subject             -0.449264792 0.007078005
## cor_emotion.L:live.L.emotion.L|subject   -0.104798436 0.505026269
## sd_live.L|subject                         0.409853248 0.604722367
## cor_emotion.L:live.L.live.L|subject       0.052038722 0.709037226
## sd_emotion.L:live.L|subject               0.245039253 0.493739189
## sigma                                     0.798389582 0.867089063
## (Intercept)                               5.389562996 5.663628493
## emotion.L                                 0.964664498 1.450029936
## live.L                                    0.175207236 0.411540944
## IRIEC                                     0.005271158 0.059455160
## AQ                                       -0.005694609 0.034771913
## emotion.L:live.L                          0.040901042 0.267609596
## emotion.L:IRIEC                           0.029674369 0.125633337
## live.L:IRIEC                             -0.019689557 0.027034699
## emotion.L:AQ                             -0.023256920 0.048408618
## live.L:AQ                                -0.014832708 0.020062612
## emotion.L:live.L:IRIEC                   -0.019672923 0.025148395
## emotion.L:live.L:AQ                      -0.020365456 0.013108684
```

```
aro_2f1.em1 <- emmeans(aro_2f1, list(pairwise ~ live|emotion),  adjust = "tukey")
```

```
## NOTE: Results may be misleading due to involvement in interactions
```

```
aro_2f1.em1
```

```
## $`emmeans of live | emotion`
## emotion = Negative:
##  live  emmean    SE df lower.CL upper.CL
##  Video   4.54 0.122 91     4.30     4.79
##  Live    4.80 0.137 91     4.53     5.07
## 
## emotion = Positive:
##  live  emmean    SE df lower.CL upper.CL
##  Video   6.10 0.122 91     5.85     6.34
##  Live    6.66 0.115 91     6.44     6.89
## 
## Degrees-of-freedom method: kenward-roger 
## Confidence level used: 0.95 
## 
## $`pairwise differences of live | emotion`
## emotion = Negative:
##  2            estimate    SE df t.ratio p.value
##  Video - Live   -0.261 0.092 91  -2.833  0.0057
## 
## emotion = Positive:
##  2            estimate    SE df t.ratio p.value
##  Video - Live   -0.569 0.114 91  -4.987  <.0001
## 
## Degrees-of-freedom method: kenward-roger
```

```
aro_2f1.em11 <- emmeans(aro_2f1, list(pairwise ~ emotion|live),  adjust = "tukey")
```

```
## NOTE: Results may be misleading due to involvement in interactions
```

```
aro_2f1.em11
```

```
## $`emmeans of emotion | live`
## live = Video:
##  emotion  emmean    SE df lower.CL upper.CL
##  Negative   4.54 0.122 91     4.30     4.79
##  Positive   6.10 0.122 91     5.85     6.34
## 
## live = Live:
##  emotion  emmean    SE df lower.CL upper.CL
##  Negative   4.80 0.137 91     4.53     5.07
##  Positive   6.66 0.115 91     6.44     6.89
## 
## Degrees-of-freedom method: kenward-roger 
## Confidence level used: 0.95 
## 
## $`pairwise differences of emotion | live`
## live = Video:
##  2                   estimate    SE df t.ratio p.value
##  Negative - Positive    -1.55 0.178 91  -8.729  <.0001
## 
## live = Live:
##  2                   estimate    SE df t.ratio p.value
##  Negative - Positive    -1.86 0.193 91  -9.656  <.0001
## 
## Degrees-of-freedom method: kenward-roger
```

```
aro_2f1.em2 <- emtrends(aro_2f1, list(pairwise ~ emotion), var = "IRIEC", adjust = "tukey")
```

```
## NOTE: Results may be misleading due to involvement in interactions
```

```
aro_2f1.em2
```

```
## $`emmeans of emotion`
##  emotion  IRIEC.trend     SE df lower.CL upper.CL
##  Negative     -0.0225 0.0240 91  -0.0701    0.025
##  Positive      0.0873 0.0205 91   0.0466    0.128
## 
## Results are averaged over the levels of: live 
## Degrees-of-freedom method: kenward-roger 
## Confidence level used: 0.95 
## 
## $`pairwise differences of emotion`
##  1                   estimate     SE df t.ratio p.value
##  Negative - Positive    -0.11 0.0348 91  -3.153  0.0022
## 
## Results are averaged over the levels of: live 
## Degrees-of-freedom method: kenward-roger
```

```
aro_2f1.predict2 <- ggemmeans(aro_2f1, terms = c("IRIEC","emotion"))
```

```
## NOTE: Results may be misleading due to involvement in interactions
```

```
aro_2f1.IRIECemo <- plot(aro_2f1.predict2,  colors = c("steelblue","red"), show.title = F)  +
  labs(x = "Mean-Centered IRIEC", y = "Arousal", colour = "Emotion") +
  guides(colour = guide_legend(reverse = T)) +
  theme(title = element_text(size = 8, face = "bold"), axis.title.x = element_text(size = 8, face = "bold"), 
        axis.title.y = element_text(size = 8, face = "bold"), strip.text = element_text(size = 8, face = "bold"),
        legend.position = "none",
        legend.title = element_text(size = 6, face = "bold"), legend.text = element_text(size = 6), 
        axis.text = element_text(size = 6))
aro_2f1.IRIECemo # Fig. 4B
```

```
aro_2f1.em22 <- emmeans(aro_2f1,  ~ emotion*IRIEC, at=list(IRIEC=c(-15.504, -10.336, -5.168, 0, 5.168, 10.336, 15.504), emotion = c("Positive","Negative")), adjust = "sidak")
```

```
## NOTE: Results may be misleading due to involvement in interactions
```

```
aro_2f1.em22 # -3SD to +3SD
```

```
##  emotion   IRIEC emmean    SE df lower.CL upper.CL
##  Positive -15.50   5.03 0.334 91     4.03     6.02
##  Negative -15.50   5.02 0.391 91     3.86     6.19
##  Positive -10.34   5.48 0.236 91     4.78     6.18
##  Negative -10.34   4.91 0.276 91     4.08     5.73
##  Positive  -5.17   5.93 0.148 91     5.49     6.37
##  Negative  -5.17   4.79 0.173 91     4.27     5.31
##  Positive   0.00   6.38 0.104 91     6.07     6.69
##  Negative   0.00   4.67 0.121 91     4.31     5.03
##  Positive   5.17   6.83 0.148 91     6.39     7.27
##  Negative   5.17   4.56 0.173 91     4.04     5.07
##  Positive  10.34   7.28 0.236 91     6.58     7.99
##  Negative  10.34   4.44 0.276 91     3.62     5.26
##  Positive  15.50   7.73 0.334 91     6.74     8.73
##  Negative  15.50   4.32 0.391 91     3.16     5.49
## 
## Results are averaged over the levels of: live 
## Degrees-of-freedom method: kenward-roger 
## Confidence level used: 0.95 
## Conf-level adjustment: sidak method for 14 estimates
```

```
contrast(aro_2f1.em22, "pairwise", by="IRIEC") # Table 4
```

```
## IRIEC = -15.50:
##  contrast            estimate    SE df t.ratio p.value
##  Positive - Negative  0.00481 0.568 91   0.008  0.9933
## 
## IRIEC = -10.34:
##  contrast            estimate    SE df t.ratio p.value
##  Positive - Negative  0.57236 0.401 91   1.428  0.1567
## 
## IRIEC =  -5.17:
##  contrast            estimate    SE df t.ratio p.value
##  Positive - Negative  1.13990 0.252 91   4.526  <.0001
## 
## IRIEC =   0.00:
##  contrast            estimate    SE df t.ratio p.value
##  Positive - Negative  1.70745 0.176 91   9.693  <.0001
## 
## IRIEC =   5.17:
##  contrast            estimate    SE df t.ratio p.value
##  Positive - Negative  2.27499 0.252 91   9.033  <.0001
## 
## IRIEC =  10.34:
##  contrast            estimate    SE df t.ratio p.value
##  Positive - Negative  2.84254 0.401 91   7.093  <.0001
## 
## IRIEC =  15.50:
##  contrast            estimate    SE df t.ratio p.value
##  Positive - Negative  3.41008 0.568 91   6.004  <.0001
## 
## Results are averaged over the levels of: live 
## Degrees-of-freedom method: kenward-roger
```

```
aro_r2f1 <- rlmer(ARO~1+emotion*live*IRIEC+emotion*live*AQ+
                  (1+emotion*live|subject), data = Rating, REML = TRUE,
                control=lmerControl(optimizer="bobyqa",
                                    optCtrl=list(maxfun=2e8)))
```

```
## Warning in .rlmerInit(lcall, pf, formula, data, method, rho.e, rho.b,
## rho.sigma.e, : Method 'DAStau' does not support blocks of size larger than 2.
## Falling back to method 'DASvar'.
```

```
summary(aro_r2f1)
```

```
## Robust linear mixed model fit by DASvar 
## Formula: ARO ~ 1 + emotion * live * IRIEC + emotion * live * AQ + (1 +      emotion * live | subject) 
##    Data: Rating 
## Control: lmerControl(optimizer = "bobyqa", optCtrl = list(maxfun = 2e+08)) 
## 
## Scaled residuals: 
##     Min      1Q  Median      3Q     Max 
## -6.3274 -0.5467  0.0221  0.5273  4.1859 
## 
## Random effects:
##  Groups   Name             Variance Std.Dev. Corr             
##  subject  (Intercept)      0.3681   0.6067                    
##           emotion.L        1.3778   1.1738   -0.16            
##           live.L           0.2165   0.4653    0.01 -0.27      
##           emotion.L:live.L 0.1265   0.3556    0.10  0.28  0.45
##  Residual                  0.4831   0.6951                    
## Number of obs: 1504, groups: subject, 94
## 
## Fixed effects:
##                         Estimate Std. Error t value
## (Intercept)             5.523100   0.066611   82.92
## emotion.L               1.244059   0.126560    9.83
## live.L                  0.277217   0.055555    4.99
## IRIEC                   0.025295   0.013169    1.92
## AQ                      0.011044   0.009835    1.12
## emotion.L:live.L        0.175166   0.052535    3.33
## emotion.L:IRIEC         0.083096   0.025022    3.32
## live.L:IRIEC            0.003315   0.010984    0.30
## emotion.L:AQ            0.013496   0.018687    0.72
## live.L:AQ               0.002273   0.008203    0.28
## emotion.L:live.L:IRIEC -0.008897   0.010386   -0.86
## emotion.L:live.L:AQ    -0.007986   0.007757   -1.03
## 
## Correlation of Fixed Effects:
##             (Intr) emtn.L live.L IRIEC  AQ     em.L:.L e.L:IR l.L:IR e.L:AQ
## emotion.L   -0.147                                                         
## live.L       0.010 -0.234                                                  
## IRIEC        0.000  0.000  0.000                                           
## AQ           0.000  0.000  0.000  0.178                                    
## emtn.L:lv.L  0.066  0.197  0.282  0.000  0.000                             
## emt.L:IRIEC  0.000  0.000  0.000 -0.147 -0.026  0.000                      
## liv.L:IRIEC  0.000  0.000  0.000  0.010  0.002  0.000  -0.234              
## emotin.L:AQ  0.000  0.000  0.000 -0.026 -0.147  0.000   0.178 -0.042       
## live.L:AQ    0.000  0.000  0.000  0.002  0.010  0.000  -0.042  0.178 -0.234
## e.L:.L:IRIE  0.000  0.000  0.000  0.066  0.012  0.000   0.197  0.282  0.035
## emt.L:.L:AQ  0.000  0.000  0.000  0.012  0.066  0.000   0.035  0.050  0.197
##             l.L:AQ e.L:.L:I
## emotion.L                  
## live.L                     
## IRIEC                      
## AQ                         
## emtn.L:lv.L                
## emt.L:IRIEC                
## liv.L:IRIEC                
## emotin.L:AQ                
## live.L:AQ                  
## e.L:.L:IRIE  0.050         
## emt.L:.L:AQ  0.282  0.178  
## 
## Robustness weights for the residuals: 
##  1214 weights are ~= 1. The remaining 290 ones are summarized as
##    Min. 1st Qu.  Median    Mean 3rd Qu.    Max. 
##   0.213   0.573   0.737   0.722   0.900   0.999 
## 
## Robustness weights for the random effects: 
##  332 weights are ~= 1. The remaining 44 ones are summarized as
##    Min. 1st Qu.  Median    Mean 3rd Qu.    Max. 
##   0.148   0.358   0.517   0.598   0.943   0.979 
## 
## Rho functions used for fitting:
##   Residuals:
##     eff: smoothed Huber (k = 1.345, s = 10) 
##     sig: smoothed Huber, Proposal 2 (k = 1.345, s = 10) 
##   Random Effects, variance component 1 (subject):
##     eff: smoothed Huber (k = 5.91, s = 10) 
##     vcp: smoothed Huber (k = 5.91, s = 10)
```

```
coefs_aro_2f1 <- data.frame(coef(summary(aro_2f1, ddf = "Satterthwaite")))
coefs_aro_r2f1 <- data.frame(coef(summary(aro_r2f1)))
p_values_aro_r2f1 <- 2 * pt(abs(coefs_aro_r2f1[, "t.value"]), coefs_aro_2f1$df, lower = FALSE)
p_values_aro_r2f1
```

```
##  [1] 1.601269e-87 5.791046e-16 2.885248e-06 5.788808e-02 2.644270e-01
##  [6] 1.238521e-03 1.292178e-03 7.634910e-01 4.720291e-01 7.822983e-01
## [11] 3.938964e-01 3.059636e-01
```

```
## Aro: AQ, IRIEC model

aro50_2f0 <- lmer(ARO~1+emotion*live*IRIEC+emotion*live*AQ+
                  (1+emotion+live|subject), data = Rating50, REML = TRUE,
                control=lmerControl(optimizer="bobyqa",
                                    optCtrl=list(maxfun=2e8)))

aro50_2f1 <- lmer(ARO~1+emotion*live*IRIEC+emotion*live*AQ+
                  (1+emotion*live|subject), data = Rating50, REML = TRUE,
                control=lmerControl(optimizer="bobyqa",
                                    optCtrl=list(maxfun=2e8)))
anova(aro50_2f0, aro50_2f1) # p = 0.009396 ***
```

```
## refitting model(s) with ML (instead of REML)
```

```
## Data: Rating50
## Models:
## aro50_2f0: ARO ~ 1 + emotion * live * IRIEC + emotion * live * AQ + (1 + emotion + live | subject)
## aro50_2f1: ARO ~ 1 + emotion * live * IRIEC + emotion * live * AQ + (1 + emotion * live | subject)
##           npar    AIC    BIC  logLik deviance Chisq Df Pr(>Chisq)   
## aro50_2f0   19 2386.6 2475.6 -1174.3   2348.6                       
## aro50_2f1   23 2381.1 2488.9 -1167.6   2335.1 13.42  4   0.009396 **
## ---
## Signif. codes:  0 '***' 0.001 '**' 0.01 '*' 0.05 '.' 0.1 ' ' 1
```

```
summary(aro50_2f1, ddf = "Satterthwaite")
```

```
## Linear mixed model fit by REML. t-tests use Satterthwaite's method [
## lmerModLmerTest]
## Formula: ARO ~ 1 + emotion * live * IRIEC + emotion * live * AQ + (1 +  
##     emotion * live | subject)
##    Data: Rating50
## Control: lmerControl(optimizer = "bobyqa", optCtrl = list(maxfun = 2e+08))
## 
## REML criterion at convergence: 2396.3
## 
## Scaled residuals: 
##     Min      1Q  Median      3Q     Max 
## -3.3649 -0.5500  0.0812  0.5182  3.3199 
## 
## Random effects:
##  Groups   Name             Variance Std.Dev. Corr             
##  subject  (Intercept)      0.52927  0.7275                    
##           emotion.L        1.50522  1.2269   -0.10            
##           live.L           0.28382  0.5327   -0.19 -0.25      
##           emotion.L:live.L 0.08535  0.2921    0.32  0.23  0.60
##  Residual                  0.71292  0.8443                    
## Number of obs: 800, groups:  subject, 50
## 
## Fixed effects:
##                         Estimate Std. Error        df t value Pr(>|t|)    
## (Intercept)             5.489420   0.108748 46.999988  50.478  < 2e-16 ***
## emotion.L               1.366062   0.181268 47.000007   7.536 1.26e-09 ***
## live.L                  0.165102   0.087669 46.999990   1.883  0.06586 .  
## IRIEC                   0.034173   0.021563 46.999988   1.585  0.11971    
## AQ                      0.032476   0.016572 46.999988   1.960  0.05598 .  
## emotion.L:live.L        0.046090   0.073703 47.000072   0.625  0.53477    
## emotion.L:IRIEC         0.118424   0.035942 47.000008   3.295  0.00188 ** 
## live.L:IRIEC           -0.008433   0.017383 46.999990  -0.485  0.62986    
## emotion.L:AQ            0.005082   0.027623 47.000007   0.184  0.85483    
## live.L:AQ              -0.002458   0.013360 46.999990  -0.184  0.85482    
## emotion.L:live.L:IRIEC  0.004373   0.014614 47.000072   0.299  0.76606    
## emotion.L:live.L:AQ    -0.003320   0.011232 47.000072  -0.296  0.76884    
## ---
## Signif. codes:  0 '***' 0.001 '**' 0.01 '*' 0.05 '.' 0.1 ' ' 1
## 
## Correlation of Fixed Effects:
##             (Intr) emtn.L live.L IRIEC  AQ     em.L:.L e.L:IR l.L:IR e.L:AQ
## emotion.L   -0.095                                                         
## live.L      -0.159 -0.215                                                  
## IRIEC       -0.148  0.014  0.024                                           
## AQ          -0.084  0.008  0.013 -0.016                                    
## emtn.L:lv.L  0.173  0.128  0.300 -0.026 -0.015                             
## emt.L:IRIEC  0.014 -0.148  0.032 -0.095  0.002 -0.019                      
## liv.L:IRIEC  0.024  0.032 -0.148 -0.159  0.003 -0.044  -0.215              
## emotin.L:AQ  0.008 -0.084  0.018  0.002 -0.095 -0.011  -0.016  0.003       
## live.L:AQ    0.013  0.018 -0.084  0.003 -0.159 -0.025   0.003 -0.016 -0.215
## e.L:.L:IRIE -0.026 -0.019 -0.044  0.173 -0.003 -0.148   0.128  0.300 -0.002
## emt.L:.L:AQ -0.015 -0.011 -0.025 -0.003  0.173 -0.084  -0.002 -0.005  0.128
##             l.L:AQ e.L:.L:I
## emotion.L                  
## live.L                     
## IRIEC                      
## AQ                         
## emtn.L:lv.L                
## emt.L:IRIEC                
## liv.L:IRIEC                
## emotin.L:AQ                
## live.L:AQ                  
## e.L:.L:IRIE -0.005         
## emt.L:.L:AQ  0.300 -0.016
```

```
aro50_r2f1 <- rlmer(ARO~1+emotion*live*IRIEC+emotion*live*AQ+
                  (1+emotion*live|subject), data = Rating50, REML = TRUE,
                control=lmerControl(optimizer="bobyqa",
                                    optCtrl=list(maxfun=2e8)))
```

```
## Warning in .rlmerInit(lcall, pf, formula, data, method, rho.e, rho.b,
## rho.sigma.e, : Method 'DAStau' does not support blocks of size larger than 2.
## Falling back to method 'DASvar'.
```

```
summary(aro50_r2f1)
```

```
## Robust linear mixed model fit by DASvar 
## Formula: ARO ~ 1 + emotion * live * IRIEC + emotion * live * AQ + (1 +      emotion * live | subject) 
##    Data: Rating50 
## Control: lmerControl(optimizer = "bobyqa", optCtrl = list(maxfun = 2e+08)) 
## 
## Scaled residuals: 
##     Min      1Q  Median      3Q     Max 
## -4.0631 -0.5865  0.0638  0.5242  3.7026 
## 
## Random effects:
##  Groups   Name             Variance Std.Dev. Corr             
##  subject  (Intercept)      0.47471  0.6890                    
##           emotion.L        1.41681  1.1903   -0.01            
##           live.L           0.18326  0.4281   -0.07 -0.07      
##           emotion.L:live.L 0.07425  0.2725    0.47  0.47  0.67
##  Residual                  0.57621  0.7591                    
## Number of obs: 800, groups: subject, 50
## 
## Fixed effects:
##                         Estimate Std. Error t value
## (Intercept)             5.494281   0.104980   52.34
## emotion.L               1.398591   0.179231    7.80
## live.L                  0.110928   0.074260    1.49
## IRIEC                   0.021892   0.020816    1.05
## AQ                      0.024346   0.015998    1.52
## emotion.L:live.L        0.064646   0.068736    0.94
## emotion.L:IRIEC         0.130860   0.035538    3.68
## live.L:IRIEC           -0.004361   0.014724   -0.30
## emotion.L:AQ            0.010818   0.027313    0.40
## live.L:AQ              -0.009658   0.011316   -0.85
## emotion.L:live.L:IRIEC -0.002296   0.013629   -0.17
## emotion.L:live.L:AQ    -0.007414   0.010475   -0.71
## 
## Correlation of Fixed Effects:
##             (Intr) emtn.L live.L IRIEC  AQ     em.L:.L e.L:IR l.L:IR e.L:AQ
## emotion.L   -0.006                                                         
## live.L      -0.058 -0.059                                                  
## IRIEC       -0.148  0.001  0.009                                           
## AQ          -0.084  0.001  0.005 -0.016                                    
## emtn.L:lv.L  0.265  0.269  0.330 -0.039 -0.022                             
## emt.L:IRIEC  0.001 -0.148  0.009 -0.006  0.000 -0.040                      
## liv.L:IRIEC  0.009  0.009 -0.148 -0.058  0.001 -0.049  -0.059              
## emotin.L:AQ  0.001 -0.084  0.005  0.000 -0.006 -0.023  -0.016  0.001       
## live.L:AQ    0.005  0.005 -0.084  0.001 -0.058 -0.028   0.001 -0.016 -0.059
## e.L:.L:IRIE -0.039 -0.040 -0.049  0.265 -0.004 -0.148   0.269  0.330 -0.004
## emt.L:.L:AQ -0.022 -0.023 -0.028 -0.004  0.265 -0.084  -0.004 -0.005  0.269
##             l.L:AQ e.L:.L:I
## emotion.L                  
## live.L                     
## IRIEC                      
## AQ                         
## emtn.L:lv.L                
## emt.L:IRIEC                
## liv.L:IRIEC                
## emotin.L:AQ                
## live.L:AQ                  
## e.L:.L:IRIE -0.005         
## emt.L:.L:AQ  0.330 -0.016  
## 
## Robustness weights for the residuals: 
##  641 weights are ~= 1. The remaining 159 ones are summarized as
##    Min. 1st Qu.  Median    Mean 3rd Qu.    Max. 
##   0.331   0.602   0.823   0.774   0.934   0.999 
## 
## Robustness weights for the random effects: 
##  176 weights are ~= 1. The remaining 24 ones are summarized as
##    Min. 1st Qu.  Median    Mean 3rd Qu.    Max. 
##   0.280   0.306   0.527   0.543   0.657   0.961 
## 
## Rho functions used for fitting:
##   Residuals:
##     eff: smoothed Huber (k = 1.345, s = 10) 
##     sig: smoothed Huber, Proposal 2 (k = 1.345, s = 10) 
##   Random Effects, variance component 1 (subject):
##     eff: smoothed Huber (k = 5.91, s = 10) 
##     vcp: smoothed Huber (k = 5.91, s = 10)
```

```
coefs_aro50_2f1 <- data.frame(coef(summary(aro50_2f1, ddf = "Satterthwaite")))
coefs_aro50_r2f1 <- data.frame(coef(summary(aro50_r2f1)))
p_values_aro50_r2f1 <- 2 * pt(abs(coefs_aro50_r2f1[, "t.value"]), coefs_aro50_2f1$df, lower = FALSE)
p_values_aro50_r2f1
```

```
##  [1] 2.535033e-43 5.015485e-10 1.419177e-01 2.983095e-01 1.347461e-01
##  [6] 3.517738e-01 5.956196e-04 7.683979e-01 6.938491e-01 3.977174e-01
## [11] 8.669681e-01 4.825614e-01
```

```
CStbl <- read.delim("Live_EMGTrait_94_complete.txt", sep = "\t")
CStbl$trial <- factor(CStbl$trial)
CStbl$emotion[CStbl$emotion == 1] <- "Positive"
CStbl$emotion[CStbl$emotion == 2] <- "Negative"
CStbl$live[CStbl$live == 1] <- "Video"
CStbl$live[CStbl$live == 2] <- "Live"
CStbl$emotion <- factor(CStbl$emotion, levels=c('Negative','Positive'),ordered=TRUE)
CStbl$live <- factor(CStbl$live, levels=c('Video','Live'),ordered=TRUE)
CStbl$Type <- factor(CStbl$Type)

CS50idx <- which(CStbl$subject<=50)
CStbl50 <- CStbl[CS50idx,]

CStbl$subject <- factor(CStbl$subject)
CStbl50$subject <- factor(CStbl50$subject)


ZMtbl <- read.delim("Live_EMGTrait_94_complete.txt", sep = "\t")
ZMtbl$trial <- factor(ZMtbl$trial)
ZMtbl$emotion[ZMtbl$emotion == 1] <- "Positive"
ZMtbl$emotion[ZMtbl$emotion == 2] <- "Negative"
ZMtbl$live[ZMtbl$live == 1] <- "Video"
ZMtbl$live[ZMtbl$live == 2] <- "Live"
ZMtbl$emotion <- factor(ZMtbl$emotion, levels=c('Negative','Positive'),ordered=TRUE)
ZMtbl$live <- factor(ZMtbl$live, levels=c('Video','Live'),ordered=TRUE)
ZMtbl$Type <- factor(ZMtbl$Type)

ZM50idx <- which(ZMtbl$subject<=50)
ZMtbl50 <- ZMtbl[ZM50idx,]
ZMtbl$subject <- factor(ZMtbl$subject)
ZMtbl50$subject <- factor(ZMtbl50$subject)
```

```
ZM_2f1 <- lmer(ZM_neut_2500~1+emotion*live*IRIEC+emotion*live*AQ+
                 (1+emotion|subject), data = ZMtbl, REML = TRUE,
               control=lmerControl(optimizer="bobyqa",
                                   optCtrl=list(maxfun=2e8)))

ZM_2f2 <- lmer(ZM_neut_2500~1+emotion*live*IRIEC+emotion*live*AQ+(1+emotion+live|subject),
               data = ZMtbl, REML = TRUE,
               control=lmerControl(optimizer="bobyqa",
                                   optCtrl=list(maxfun=2e8)))
anova(ZM_2f1, ZM_2f2) # p = 0.0001313  *** # winning model
```

```
## refitting model(s) with ML (instead of REML)
```

```
## Data: ZMtbl
## Models:
## ZM_2f1: ZM_neut_2500 ~ 1 + emotion * live * IRIEC + emotion * live * AQ + (1 + emotion | subject)
## ZM_2f2: ZM_neut_2500 ~ 1 + emotion * live * IRIEC + emotion * live * AQ + (1 + emotion + live | subject)
##        npar     AIC     BIC logLik deviance  Chisq Df Pr(>Chisq)    
## ZM_2f1   16 -2034.4 -1917.8 1033.2  -2066.4                         
## ZM_2f2   19 -2048.9 -1910.5 1043.5  -2086.9 20.538  3  0.0001313 ***
## ---
## Signif. codes:  0 '***' 0.001 '**' 0.01 '*' 0.05 '.' 0.1 ' ' 1
```

```
ZM_2f3 <- lmer(ZM_neut_2500~1+emotion*live*IRIEC+emotion*live*AQ+(1+emotion*live|subject),
               data = ZMtbl, REML = TRUE,
               control=lmerControl(optimizer="bobyqa",
                                   optCtrl=list(maxfun=2e8)))
anova(ZM_2f2, ZM_2f3) # p = 0.09036, no need to add random slope for emotion:live|subject
```

```
## refitting model(s) with ML (instead of REML)
```

```
## Data: ZMtbl
## Models:
## ZM_2f2: ZM_neut_2500 ~ 1 + emotion * live * IRIEC + emotion * live * AQ + (1 + emotion + live | subject)
## ZM_2f3: ZM_neut_2500 ~ 1 + emotion * live * IRIEC + emotion * live * AQ + (1 + emotion * live | subject)
##        npar     AIC     BIC logLik deviance  Chisq Df Pr(>Chisq)  
## ZM_2f2   19 -2048.9 -1910.5 1043.5  -2086.9                       
## ZM_2f3   23 -2048.9 -1881.4 1047.5  -2094.9 8.0335  4    0.09036 .
## ---
## Signif. codes:  0 '***' 0.001 '**' 0.01 '*' 0.05 '.' 0.1 ' ' 1
```

```
ZM_2f4 <- lmer(ZM_neut_2500~1+emotion*live*IRIEC+emotion*live*AQ+(1+emotion+live|subject)+(1|Type),
               data = ZMtbl, REML = TRUE,
               control=lmerControl(optimizer="bobyqa",
                                   optCtrl=list(maxfun=2e8)))
anova(ZM_2f2, ZM_2f4) # p = 0.9242
```

```
## refitting model(s) with ML (instead of REML)
```

```
## Data: ZMtbl
## Models:
## ZM_2f2: ZM_neut_2500 ~ 1 + emotion * live * IRIEC + emotion * live * AQ + (1 + emotion + live | subject)
## ZM_2f4: ZM_neut_2500 ~ 1 + emotion * live * IRIEC + emotion * live * AQ + (1 + emotion + live | subject) + (1 | Type)
##        npar     AIC     BIC logLik deviance  Chisq Df Pr(>Chisq)
## ZM_2f2   19 -2048.9 -1910.5 1043.5  -2086.9                     
## ZM_2f4   20 -2046.9 -1901.2 1043.5  -2086.9 0.0091  1     0.9242
```

```
summary(ZM_2f4) # # Table 5
```

```
## Linear mixed model fit by REML. t-tests use Satterthwaite's method [
## lmerModLmerTest]
## Formula: ZM_neut_2500 ~ 1 + emotion * live * IRIEC + emotion * live *  
##     AQ + (1 + emotion + live | subject) + (1 | Type)
##    Data: ZMtbl
## Control: lmerControl(optimizer = "bobyqa", optCtrl = list(maxfun = 2e+08))
## 
## REML criterion at convergence: -1956.8
## 
## Scaled residuals: 
##      Min       1Q   Median       3Q      Max 
## -11.2802  -0.3726  -0.0082   0.3631   8.5890 
## 
## Random effects:
##  Groups   Name        Variance  Std.Dev. Corr     
##  subject  (Intercept) 9.968e-03 0.099842          
##           emotion.L   1.675e-02 0.129422 0.91     
##           live.L      4.490e-04 0.021188 0.55 0.76
##  Type     (Intercept) 8.541e-05 0.009242          
##  Residual             4.629e-02 0.215160          
## Number of obs: 10787, groups:  subject, 93; Type, 2
## 
## Fixed effects:
##                          Estimate Std. Error         df t value Pr(>|t|)    
## (Intercept)             3.195e-02  1.247e-02  4.047e+00   2.563 0.061744 .  
## emotion.L               4.242e-02  1.383e-02  8.549e+01   3.067 0.002894 ** 
## live.L                  2.294e-03  3.743e-03  7.410e+01   0.613 0.541872    
## IRIEC                   6.399e-04  2.100e-03  8.501e+01   0.305 0.761329    
## AQ                      1.166e-03  1.571e-03  8.479e+01   0.742 0.459885    
## emotion.L:live.L        1.380e-02  4.166e-03  1.054e+04   3.312 0.000928 ***
## emotion.L:IRIEC         6.131e-04  2.728e-03  8.559e+01   0.225 0.822732    
## live.L:IRIEC            6.346e-05  7.518e-04  8.081e+01   0.084 0.932940    
## emotion.L:AQ           -1.511e-03  2.043e-03  8.528e+01  -0.740 0.461632    
## live.L:AQ              -3.320e-04  5.562e-04  7.641e+01  -0.597 0.552350    
## emotion.L:live.L:IRIEC -3.264e-04  8.485e-04  1.054e+04  -0.385 0.700466    
## emotion.L:live.L:AQ    -9.896e-06  6.226e-04  1.054e+04  -0.016 0.987318    
## ---
## Signif. codes:  0 '***' 0.001 '**' 0.01 '*' 0.05 '.' 0.1 ' ' 1
## 
## Correlation of Fixed Effects:
##             (Intr) emtn.L live.L IRIEC  AQ     em.L:.L e.L:IR l.L:IR e.L:AQ
## emotion.L    0.734                                                         
## live.L       0.268  0.439                                                  
## IRIEC       -0.003 -0.004 -0.005                                           
## AQ          -0.008 -0.009 -0.005  0.192                                    
## emtn.L:lv.L -0.001  0.000 -0.005  0.000  0.000                             
## emt.L:IRIEC -0.006 -0.003 -0.003  0.859  0.160  0.000                      
## liv.L:IRIEC -0.002 -0.003  0.057  0.308  0.057  0.000   0.432              
## emotin.L:AQ -0.009 -0.008 -0.005  0.159  0.861  0.000   0.191  0.081       
## live.L:AQ   -0.003 -0.005  0.041  0.058  0.313  0.000   0.082  0.251  0.437
## e.L:.L:IRIE  0.000  0.000  0.000 -0.001  0.000  0.091   0.000 -0.004  0.000
## emt.L:.L:AQ  0.000  0.000  0.000  0.000 -0.001  0.072   0.000  0.000  0.000
##             l.L:AQ e.L:.L:I
## emotion.L                  
## live.L                     
## IRIEC                      
## AQ                         
## emtn.L:lv.L                
## emt.L:IRIEC                
## liv.L:IRIEC                
## emotin.L:AQ                
## live.L:AQ                  
## e.L:.L:IRIE  0.000         
## emt.L:.L:AQ -0.007  0.283
```

```
r2beta.ZM_2f4 <- r2beta(ZM_2f4, partial = TRUE, method = 'nsj')
r2beta.ZM_2f4
```

```
##                    Effect   Rsq upper.CL lower.CL
## 1                   Model 0.017    0.023    0.013
## 2               emotion.L 0.013    0.018    0.010
## 5                      AQ 0.001    0.002    0.000
## 9            emotion.L:AQ 0.001    0.002    0.000
## 6        emotion.L:live.L 0.001    0.002    0.000
## 4                   IRIEC 0.000    0.001    0.000
## 7         emotion.L:IRIEC 0.000    0.001    0.000
## 3                  live.L 0.000    0.001    0.000
## 10              live.L:AQ 0.000    0.001    0.000
## 11 emotion.L:live.L:IRIEC 0.000    0.001    0.000
## 8            live.L:IRIEC 0.000    0.000    0.000
## 12    emotion.L:live.L:AQ 0.000    0.000    0.000
```

```
conf.ZM_2f4 <- confint(ZM_2f4, oldNames = F)
```

```
## Computing profile confidence intervals ...
```

```
## Warning in FUN(X[[i]], ...): non-monotonic profile for sd_(Intercept)|subject
```

```
## Warning in FUN(X[[i]], ...): non-monotonic profile for
## cor_live.L.(Intercept)|subject
```

```
## Warning in FUN(X[[i]], ...): non-monotonic profile for sd_emotion.L|subject
```

```
## Warning in nextpar(mat, cc, i, delta, lowcut, upcut): unexpected decrease in
## profile: using minstep

## Warning in nextpar(mat, cc, i, delta, lowcut, upcut): unexpected decrease in
## profile: using minstep

## Warning in nextpar(mat, cc, i, delta, lowcut, upcut): unexpected decrease in
## profile: using minstep

## Warning in nextpar(mat, cc, i, delta, lowcut, upcut): unexpected decrease in
## profile: using minstep

## Warning in nextpar(mat, cc, i, delta, lowcut, upcut): unexpected decrease in
## profile: using minstep

## Warning in nextpar(mat, cc, i, delta, lowcut, upcut): unexpected decrease in
## profile: using minstep

## Warning in nextpar(mat, cc, i, delta, lowcut, upcut): unexpected decrease in
## profile: using minstep

## Warning in nextpar(mat, cc, i, delta, lowcut, upcut): unexpected decrease in
## profile: using minstep

## Warning in nextpar(mat, cc, i, delta, lowcut, upcut): unexpected decrease in
## profile: using minstep

## Warning in nextpar(mat, cc, i, delta, lowcut, upcut): unexpected decrease in
## profile: using minstep

## Warning in nextpar(mat, cc, i, delta, lowcut, upcut): unexpected decrease in
## profile: using minstep
```

```
## Warning in FUN(X[[i]], ...): non-monotonic profile for
## cor_live.L.emotion.L|subject
```

```
## Warning in nextpar(mat, cc, i, delta, lowcut, upcut): unexpected decrease in
## profile: using minstep

## Warning in nextpar(mat, cc, i, delta, lowcut, upcut): unexpected decrease in
## profile: using minstep

## Warning in nextpar(mat, cc, i, delta, lowcut, upcut): unexpected decrease in
## profile: using minstep

## Warning in nextpar(mat, cc, i, delta, lowcut, upcut): unexpected decrease in
## profile: using minstep

## Warning in nextpar(mat, cc, i, delta, lowcut, upcut): unexpected decrease in
## profile: using minstep

## Warning in nextpar(mat, cc, i, delta, lowcut, upcut): unexpected decrease in
## profile: using minstep

## Warning in nextpar(mat, cc, i, delta, lowcut, upcut): unexpected decrease in
## profile: using minstep

## Warning in nextpar(mat, cc, i, delta, lowcut, upcut): unexpected decrease in
## profile: using minstep

## Warning in nextpar(mat, cc, i, delta, lowcut, upcut): unexpected decrease in
## profile: using minstep
```

```
## Warning in FUN(X[[i]], ...): non-monotonic profile for sd_(Intercept)|Type
```

```
## Warning in FUN(X[[i]], ...): non-monotonic profile for sigma
```

```
## Warning in optwrap(optimizer, par = thopt, fn = mkdevfun(rho, 0L), lower =
## fitted@lower): convergence code 1 from bobyqa: bobyqa -- maximum number of
## function evaluations exceeded

## Warning in optwrap(optimizer, par = thopt, fn = mkdevfun(rho, 0L), lower =
## fitted@lower): convergence code 1 from bobyqa: bobyqa -- maximum number of
## function evaluations exceeded

## Warning in optwrap(optimizer, par = thopt, fn = mkdevfun(rho, 0L), lower =
## fitted@lower): convergence code 1 from bobyqa: bobyqa -- maximum number of
## function evaluations exceeded

## Warning in optwrap(optimizer, par = thopt, fn = mkdevfun(rho, 0L), lower =
## fitted@lower): convergence code 1 from bobyqa: bobyqa -- maximum number of
## function evaluations exceeded

## Warning in optwrap(optimizer, par = thopt, fn = mkdevfun(rho, 0L), lower =
## fitted@lower): convergence code 1 from bobyqa: bobyqa -- maximum number of
## function evaluations exceeded

## Warning in optwrap(optimizer, par = thopt, fn = mkdevfun(rho, 0L), lower =
## fitted@lower): convergence code 1 from bobyqa: bobyqa -- maximum number of
## function evaluations exceeded
```

```
## Warning in confint.thpr(pp, level = level, zeta = zeta): bad spline fit for
## sd_(Intercept)|subject: falling back to linear interpolation
```

```
## Warning in confint.thpr(pp, level = level, zeta = zeta): bad spline fit for
## cor_live.L.(Intercept)|subject: falling back to linear interpolation
```

```
## Warning in confint.thpr(pp, level = level, zeta = zeta): bad spline fit for
## sd_emotion.L|subject: falling back to linear interpolation
```

```
## Warning in confint.thpr(pp, level = level, zeta = zeta): bad spline fit for
## cor_live.L.emotion.L|subject: falling back to linear interpolation
```

```
## Warning in confint.thpr(pp, level = level, zeta = zeta): bad spline fit for
## sd_(Intercept)|Type: falling back to linear interpolation
```

```
## Warning in confint.thpr(pp, level = level, zeta = zeta): bad spline fit for
## sigma: falling back to linear interpolation
```

```
conf.ZM_2f4
```

```
##                                          2.5 %       97.5 %
## sd_(Intercept)|subject             0.097437130 0.0976532854
## cor_emotion.L.(Intercept)|subject  0.882466588 0.9460362296
## cor_live.L.(Intercept)|subject     0.556375722 0.5581630493
## sd_emotion.L|subject               0.127167214 0.1275590606
## cor_live.L.emotion.L|subject       0.426648038 0.8962920193
## sd_live.L|subject                  0.019774681 0.0295054224
## sd_(Intercept)|Type                0.002509612 0.0025279349
## sigma                              0.215094805 0.2154381851
## (Intercept)                        0.008117530 0.0557589155
## emotion.L                          0.014922074 0.0689388173
## live.L                            -0.004858819 0.0098806511
## IRIEC                             -0.003575742 0.0046047164
## AQ                                -0.001947079 0.0041549283
## emotion.L:live.L                   0.005641901 0.0219701985
## emotion.L:IRIEC                   -0.004701685 0.0059355952
## live.L:IRIEC                      -0.001397018 0.0015386168
## emotion.L:AQ                      -0.005494617 0.0024728095
## live.L:AQ                         -0.001420857 0.0007537284
## emotion.L:live.L:IRIEC            -0.001989120 0.0013365577
## emotion.L:live.L:AQ               -0.001229600 0.0012105768
```

```
emm_options(lmerTest.limit = 20000)
ZM_2f4.em1 <- emmeans(ZM_2f4, list(pairwise ~ emotion|live), adjust = "tukey")
```

```
## Note: D.f. calculations have been disabled because the number of observations exceeds 3000.
## To enable adjustments, add the argument 'pbkrtest.limit = 10787' (or larger)
## [or, globally, 'set emm_options(pbkrtest.limit = 10787)' or larger];
## but be warned that this may result in large computation time and memory use.
```

```
## NOTE: Results may be misleading due to involvement in interactions
```

```
ZM_2f4.em1
```

```
## $`emmeans of emotion | live`
## live = Video:
##  emotion    emmean      SE    df lower.CL upper.CL
##  Negative  0.00641 0.00942  1.42  -0.0548   0.0676
##  Positive  0.05286 0.02011 21.87   0.0111   0.0946
## 
## live = Live:
##  emotion    emmean      SE    df lower.CL upper.CL
##  Negative -0.00415 0.00887  1.10  -0.0961   0.0878
##  Positive  0.07016 0.02205 28.26   0.0250   0.1153
## 
## Degrees-of-freedom method: satterthwaite 
## Confidence level used: 0.95 
## 
## $`pairwise differences of emotion | live`
## live = Video:
##  2                   estimate     SE   df t.ratio p.value
##  Negative - Positive  -0.0465 0.0201 93.0  -2.312  0.0230
## 
## live = Live:
##  2                   estimate     SE   df t.ratio p.value
##  Negative - Positive  -0.0743 0.0201 93.1  -3.698  0.0004
## 
## Degrees-of-freedom method: satterthwaite
```

```
ZM_2f4.em11 <- emmeans(ZM_2f4, list(pairwise ~ live|emotion), adjust = "tukey")
```

```
## Note: D.f. calculations have been disabled because the number of observations exceeds 3000.
## To enable adjustments, add the argument 'pbkrtest.limit = 10787' (or larger)
## [or, globally, 'set emm_options(pbkrtest.limit = 10787)' or larger];
## but be warned that this may result in large computation time and memory use.
## NOTE: Results may be misleading due to involvement in interactions
```

```
ZM_2f4.em11
```

```
## $`emmeans of live | emotion`
## emotion = Negative:
##  live    emmean      SE    df lower.CL upper.CL
##  Video  0.00641 0.00942  1.42  -0.0548   0.0676
##  Live  -0.00415 0.00887  1.10  -0.0961   0.0878
## 
## emotion = Positive:
##  live    emmean      SE    df lower.CL upper.CL
##  Video  0.05286 0.02011 21.87   0.0111   0.0946
##  Live   0.07016 0.02205 28.26   0.0250   0.1153
## 
## Degrees-of-freedom method: satterthwaite 
## Confidence level used: 0.95 
## 
## $`pairwise differences of live | emotion`
## emotion = Negative:
##  2            estimate      SE  df t.ratio p.value
##  Video - Live   0.0106 0.00673 190   1.568  0.1186
## 
## emotion = Positive:
##  2            estimate      SE  df t.ratio p.value
##  Video - Live  -0.0173 0.00670 185  -2.581  0.0106
## 
## Degrees-of-freedom method: satterthwaite
```

```
ZM_r2f4 <- rlmer(ZM_neut_2500~1+emotion*live*IRIEC+emotion*live*AQ+(1+emotion+live|subject)+(1|Type),
               data = ZMtbl, REML = TRUE,
               control=lmerControl(optimizer="bobyqa",
                                   optCtrl=list(maxfun=2e8)))
```

```
## Warning in .rlmerInit(lcall, pf, formula, data, method, rho.e, rho.b,
## rho.sigma.e, : Method 'DAStau' does not support blocks of size larger than 2.
## Falling back to method 'DASvar'.
```

```
summary(ZM_r2f4)
```

```
## Robust linear mixed model fit by DASvar 
## Formula: ZM_neut_2500 ~ 1 + emotion * live * IRIEC + emotion * live *      AQ + (1 + emotion + live | subject) + (1 | Type) 
##    Data: ZMtbl 
## Control: lmerControl(optimizer = "bobyqa", optCtrl = list(maxfun = 2e+08)) 
## 
## Scaled residuals: 
##      Min       1Q   Median       3Q      Max 
## -17.8450  -0.5395  -0.0149   0.5537  13.3490 
## 
## Random effects:
##  Groups   Name        Variance  Std.Dev. Corr       
##  subject  (Intercept) 8.979e-04 0.029965            
##           emotion.L   6.740e-04 0.025962  0.03      
##           live.L      5.284e-05 0.007269 -0.52  0.84
##  Type     (Intercept) 0.000e+00 0.000000            
##  Residual             1.951e-02 0.139695            
## Number of obs: 10787, groups: subject, 93; Type, 2
## 
## Fixed effects:
##                          Estimate Std. Error t value
## (Intercept)             8.840e-03  3.529e-03   2.505
## emotion.L               1.493e-02  3.483e-03   4.287
## live.L                 -3.453e-04  2.119e-03  -0.163
## IRIEC                   3.704e-04  6.982e-04   0.531
## AQ                      6.285e-04  5.218e-04   1.204
## emotion.L:live.L        1.101e-02  2.774e-03   3.970
## emotion.L:IRIEC        -1.620e-04  6.924e-04  -0.234
## live.L:IRIEC           -2.537e-04  4.295e-04  -0.591
## emotion.L:AQ           -3.087e-04  5.157e-04  -0.599
## live.L:AQ               2.801e-05  3.161e-04   0.089
## emotion.L:live.L:IRIEC -1.165e-04  5.650e-04  -0.206
## emotion.L:live.L:AQ     2.730e-04  4.146e-04   0.659
## 
## Correlation of Fixed Effects:
##             (Intr) emtn.L live.L IRIEC  AQ     em.L:.L e.L:IR l.L:IR e.L:AQ
## emotion.L    0.021                                                         
## live.L      -0.176  0.262                                                  
## IRIEC        0.009  0.000  0.001                                           
## AQ           0.001  0.000  0.002  0.204                                    
## emtn.L:lv.L -0.002  0.000 -0.007  0.000  0.000                             
## emt.L:IRIEC  0.000  0.028  0.000  0.021  0.004  0.000                      
## liv.L:IRIEC  0.001  0.000  0.078 -0.170 -0.032  0.000   0.252              
## emotin.L:AQ  0.000  0.015 -0.003  0.004  0.020  0.000   0.223  0.050       
## live.L:AQ    0.002 -0.003  0.060 -0.033 -0.173  0.000   0.051  0.271  0.257
## e.L:.L:IRIE  0.000  0.000  0.000 -0.001  0.000  0.091   0.000 -0.005  0.000
## emt.L:.L:AQ  0.000  0.000  0.000  0.001 -0.002  0.072   0.000  0.000  0.001
##             l.L:AQ e.L:.L:I
## emotion.L                  
## live.L                     
## IRIEC                      
## AQ                         
## emtn.L:lv.L                
## emt.L:IRIEC                
## liv.L:IRIEC                
## emotin.L:AQ                
## live.L:AQ                  
## e.L:.L:IRIE  0.000         
## emt.L:.L:AQ -0.008  0.283  
## 
## Robustness weights for the residuals: 
##  8229 weights are ~= 1. The remaining 2558 ones are summarized as
##    Min. 1st Qu.  Median    Mean 3rd Qu.    Max. 
##  0.0754  0.4620  0.6720  0.6490  0.8610  0.9990 
## 
## Robustness weights for the random effects: 
##  239 weights are ~= 1. The remaining 42 ones are summarized as
##    Min. 1st Qu.  Median    Mean 3rd Qu.    Max. 
## 0.00268 0.12100 0.38100 0.40900 0.74100 0.93900 
## 
## Rho functions used for fitting:
##   Residuals:
##     eff: smoothed Huber (k = 1.345, s = 10) 
##     sig: smoothed Huber, Proposal 2 (k = 1.345, s = 10) 
##   Random Effects, variance component 1 (subject):
##     eff: smoothed Huber (k = 5.55, s = 10) 
##     vcp: smoothed Huber (k = 5.55, s = 10) 
##   Random Effects, variance component 2 (Type):
##     eff: smoothed Huber (k = 1.345, s = 10) 
##     vcp: smoothed Huber, Proposal 2 (k = 1.345, s = 10)
```

```
coefs_ZM_2f4 <- data.frame(coef(summary(ZM_2f4, ddf = "Satterthwaite")))
coefs_ZM_r2f4 <- data.frame(coef(summary(ZM_r2f4)))
p_values_ZM_r2f4 <- 2 * pt(abs(coefs_ZM_r2f4[, "t.value"]), coefs_ZM_2f4$df, lower = FALSE)
p_values_ZM_r2f4
```

```
##  [1] 6.567614e-02 4.733986e-05 8.710344e-01 5.971361e-01 2.317671e-01
##  [6] 7.227581e-05 8.155753e-01 5.563653e-01 5.510696e-01 9.296369e-01
## [11] 8.366244e-01 5.102041e-01
```

```
ZM50_2f1 <- lmer(ZM_neut_2500~1+emotion*live*IRIEC+emotion*live*AQ+
                 (1+emotion|subject), data = ZMtbl50, REML = TRUE,
               control=lmerControl(optimizer="bobyqa",
                                   optCtrl=list(maxfun=2e8)))

ZM50_2f2 <- lmer(ZM_neut_2500~1+emotion*live*IRIEC+emotion*live*AQ+(1+emotion+live|subject),
               data = ZMtbl50, REML = TRUE,
               control=lmerControl(optimizer="bobyqa",
                                   optCtrl=list(maxfun=2e8)))
```

```
## boundary (singular) fit: see help('isSingular')
```

```
# Singular
anova(ZM50_2f1, ZM50_2f2) # p = 0.0005635 ***
```

```
## refitting model(s) with ML (instead of REML)
```

```
## Data: ZMtbl50
## Models:
## ZM50_2f1: ZM_neut_2500 ~ 1 + emotion * live * IRIEC + emotion * live * AQ + (1 + emotion | subject)
## ZM50_2f2: ZM_neut_2500 ~ 1 + emotion * live * IRIEC + emotion * live * AQ + (1 + emotion + live | subject)
##          npar     AIC     BIC logLik deviance  Chisq Df Pr(>Chisq)    
## ZM50_2f1   16 -792.86 -696.78 412.43  -824.86                         
## ZM50_2f2   19 -804.33 -690.24 421.17  -842.33 17.478  3  0.0005635 ***
## ---
## Signif. codes:  0 '***' 0.001 '**' 0.01 '*' 0.05 '.' 0.1 ' ' 1
```

```
summary(ZM50_2f2, ddf = "Satterthwaite") # # Table 5
```

```
## Linear mixed model fit by REML. t-tests use Satterthwaite's method [
## lmerModLmerTest]
## Formula: ZM_neut_2500 ~ 1 + emotion * live * IRIEC + emotion * live *  
##     AQ + (1 + emotion + live | subject)
##    Data: ZMtbl50
## Control: lmerControl(optimizer = "bobyqa", optCtrl = list(maxfun = 2e+08))
## 
## REML criterion at convergence: -724.6
## 
## Scaled residuals: 
##      Min       1Q   Median       3Q      Max 
## -12.0184  -0.2460   0.0008   0.2638   6.8021 
## 
## Random effects:
##  Groups   Name        Variance  Std.Dev. Corr     
##  subject  (Intercept) 0.0166839 0.12917           
##           emotion.L   0.0312339 0.17673  0.94     
##           live.L      0.0005209 0.02282  0.97 0.99
##  Residual             0.0410098 0.20251           
## Number of obs: 2996, groups:  subject, 50
## 
## Fixed effects:
##                          Estimate Std. Error         df t value Pr(>|t|)  
## (Intercept)             4.024e-02  1.892e-02  4.699e+01   2.127   0.0387 *
## emotion.L               6.946e-02  2.592e-02  4.699e+01   2.680   0.0101 *
## live.L                  1.000e-02  6.241e-03  9.555e+01   1.603   0.1123  
## IRIEC                   1.849e-03  3.751e-03  4.699e+01   0.493   0.6244  
## AQ                     -6.667e-04  2.883e-03  4.699e+01  -0.231   0.8181  
## emotion.L:live.L        1.589e-02  7.511e-03  2.890e+03   2.116   0.0345 *
## emotion.L:IRIEC         1.982e-03  5.140e-03  4.699e+01   0.386   0.7015  
## live.L:IRIEC           -2.323e-04  1.237e-03  9.551e+01  -0.188   0.8515  
## emotion.L:AQ           -3.505e-03  3.950e-03  4.700e+01  -0.887   0.3795  
## live.L:AQ              -1.174e-03  9.518e-04  9.586e+01  -1.234   0.2204  
## emotion.L:live.L:IRIEC  2.460e-04  1.489e-03  2.890e+03   0.165   0.8688  
## emotion.L:live.L:AQ    -4.366e-04  1.146e-03  2.890e+03  -0.381   0.7032  
## ---
## Signif. codes:  0 '***' 0.001 '**' 0.01 '*' 0.05 '.' 0.1 ' ' 1
## 
## Correlation of Fixed Effects:
##             (Intr) emtn.L live.L IRIEC  AQ     em.L:.L e.L:IR l.L:IR e.L:AQ
## emotion.L    0.902                                                         
## live.L       0.501  0.511                                                  
## IRIEC       -0.148 -0.134 -0.074                                           
## AQ          -0.084 -0.076 -0.042 -0.016                                    
## emtn.L:lv.L  0.000  0.000 -0.001  0.000  0.000                             
## emt.L:IRIEC -0.134 -0.148 -0.076  0.902 -0.015  0.000                      
## liv.L:IRIEC -0.074 -0.076 -0.148  0.501 -0.008  0.000   0.510              
## emotin.L:AQ -0.076 -0.084 -0.043 -0.015  0.902  0.000  -0.016 -0.008       
## live.L:AQ   -0.042 -0.043 -0.085 -0.008  0.500  0.001  -0.008 -0.017  0.510
## e.L:.L:IRIE  0.000  0.000  0.000  0.000  0.000 -0.148   0.000 -0.001  0.000
## emt.L:.L:AQ  0.000  0.000  0.001  0.000  0.000 -0.085   0.000  0.001  0.000
##             l.L:AQ e.L:.L:I
## emotion.L                  
## live.L                     
## IRIEC                      
## AQ                         
## emtn.L:lv.L                
## emt.L:IRIEC                
## liv.L:IRIEC                
## emotin.L:AQ                
## live.L:AQ                  
## e.L:.L:IRIE  0.001         
## emt.L:.L:AQ -0.003 -0.017  
## optimizer (bobyqa) convergence code: 0 (OK)
## boundary (singular) fit: see help('isSingular')
```

```
ZM50_r2f2 <- rlmer(ZM_neut_2500~1+emotion*live*IRIEC+emotion*live*AQ+(1+emotion+live|subject),
               data = ZMtbl50, REML = TRUE,
               control=lmerControl(optimizer="bobyqa",
                                   optCtrl=list(maxfun=2e8)))
```

```
## boundary (singular) fit: see help('isSingular')
```

```
## Warning in .rlmerInit(lcall, pf, formula, data, method, rho.e, rho.b,
## rho.sigma.e, : Method 'DAStau' does not support blocks of size larger than 2.
## Falling back to method 'DASvar'.
```

```
summary(ZM50_r2f2)
```

```
## Robust linear mixed model fit by DASvar 
## Formula: ZM_neut_2500 ~ 1 + emotion * live * IRIEC + emotion * live *      AQ + (1 + emotion + live | subject) 
##    Data: ZMtbl50 
## Control: lmerControl(optimizer = "bobyqa", optCtrl = list(maxfun = 2e+08)) 
## 
## Scaled residuals: 
##      Min       1Q   Median       3Q      Max 
## -27.3150  -0.5087  -0.0114   0.5248  15.7873 
## 
## Random effects:
##  Groups   Name        Variance  Std.Dev. Corr       
##  subject  (Intercept) 8.743e-04 0.029569            
##           emotion.L   1.669e-03 0.040850  0.00      
##           live.L      3.593e-05 0.005994 -0.05  1.00
##  Residual             8.365e-03 0.091458            
## Number of obs: 2996, groups: subject, 50
## 
## Fixed effects:
##                          Estimate Std. Error t value
## (Intercept)             6.870e-03  4.660e-03   1.474
## emotion.L               2.127e-02  6.459e-03   3.294
## live.L                  2.204e-03  2.612e-03   0.844
## IRIEC                   1.519e-03  9.239e-04   1.644
## AQ                      5.594e-04  7.102e-04   0.788
## emotion.L:live.L        1.041e-02  3.479e-03   2.991
## emotion.L:IRIEC         1.078e-03  1.281e-03   0.842
## live.L:IRIEC           -6.469e-05  5.178e-04  -0.125
## emotion.L:AQ           -1.025e-04  9.844e-04  -0.104
## live.L:AQ              -5.349e-04  3.984e-04  -1.343
## emotion.L:live.L:IRIEC  6.164e-04  6.898e-04   0.894
## emotion.L:live.L:AQ    -7.667e-04  5.308e-04  -1.444
## 
## Correlation of Fixed Effects:
##             (Intr) emtn.L live.L IRIEC  AQ     em.L:.L e.L:IR l.L:IR e.L:AQ
## emotion.L    0.000                                                         
## live.L      -0.015  0.310                                                  
## IRIEC       -0.148  0.000  0.002                                           
## AQ          -0.085  0.000  0.001 -0.016                                    
## emtn.L:lv.L  0.000  0.000 -0.001  0.000  0.001                             
## emt.L:IRIEC  0.000 -0.148 -0.046  0.000  0.000  0.000                      
## liv.L:IRIEC  0.002 -0.046 -0.148 -0.015  0.000  0.000   0.310              
## emotin.L:AQ  0.000 -0.085 -0.026  0.000  0.000 -0.001  -0.016 -0.005       
## live.L:AQ    0.001 -0.026 -0.085  0.000 -0.014  0.001  -0.005 -0.017  0.309
## e.L:.L:IRIE  0.000  0.000  0.000  0.000  0.000 -0.148   0.000 -0.001  0.000
## emt.L:.L:AQ  0.001 -0.001  0.001  0.000 -0.001 -0.085   0.000  0.001  0.001
##             l.L:AQ e.L:.L:I
## emotion.L                  
## live.L                     
## IRIEC                      
## AQ                         
## emtn.L:lv.L                
## emt.L:IRIEC                
## liv.L:IRIEC                
## emotin.L:AQ                
## live.L:AQ                  
## e.L:.L:IRIE  0.001         
## emt.L:.L:AQ -0.003 -0.017  
## 
## Robustness weights for the residuals: 
##  2283 weights are ~= 1. The remaining 713 ones are summarized as
##    Min. 1st Qu.  Median    Mean 3rd Qu.    Max. 
##  0.0492  0.3250  0.5810  0.5670  0.7980  0.9990 
## 
## Robustness weights for the random effects: 
##  135 weights are ~= 1. The remaining 15 ones are summarized as
##    Min. 1st Qu.  Median    Mean 3rd Qu.    Max. 
## 0.00437 0.01440 0.02340 0.14400 0.25500 0.42500 
## 
## Rho functions used for fitting:
##   Residuals:
##     eff: smoothed Huber (k = 1.345, s = 10) 
##     sig: smoothed Huber, Proposal 2 (k = 1.345, s = 10) 
##   Random Effects, variance component 1 (subject):
##     eff: smoothed Huber (k = 5.55, s = 10) 
##     vcp: smoothed Huber (k = 5.55, s = 10)
```

```
coefs_ZM50_2f2 <- data.frame(coef(summary(ZM50_2f2, ddf = "Satterthwaite")))
coefs_ZM50_r2f2 <- data.frame(coef(summary(ZM50_r2f2)))
p_values_ZM50_r2f2 <- 2 * pt(abs(coefs_ZM50_r2f2[, "t.value"]), coefs_ZM50_2f2$df, lower = FALSE)
p_values_ZM50_r2f2
```

```
##  [1] 0.147050001 0.001884824 0.400759990 0.106854772 0.434862588 0.002801058
##  [7] 0.404060690 0.900833006 0.917540377 0.182532901 0.371581968 0.148719858
```

```
CS_2f1 <- lmer(CF_neut_2500~1+emotion*live*IRIEC+emotion*live*AQ+
                 (1+emotion+live|subject), data = CStbl, REML = TRUE,
               control=lmerControl(optimizer="bobyqa",
                                   optCtrl=list(maxfun=2e8)))

CS_2f2 <- lmer(CF_neut_2500~1+emotion*live*IRIEC+emotion*live*AQ+
                 (1+emotion*live|subject), data = CStbl, REML = TRUE,
               control=lmerControl(optimizer="bobyqa",
                                   optCtrl=list(maxfun=2e8)))
anova(CS_2f1, CS_2f2) # p = 3.356e-11 ***
```

```
## refitting model(s) with ML (instead of REML)
```

```
## Data: CStbl
## Models:
## CS_2f1: CF_neut_2500 ~ 1 + emotion * live * IRIEC + emotion * live * AQ + (1 + emotion + live | subject)
## CS_2f2: CF_neut_2500 ~ 1 + emotion * live * IRIEC + emotion * live * AQ + (1 + emotion * live | subject)
##        npar     AIC     BIC logLik deviance  Chisq Df Pr(>Chisq)    
## CS_2f1   19 -3758.6 -3620.1 1898.3  -3796.6                         
## CS_2f2   23 -3805.5 -3637.9 1925.8  -3851.5 54.933  4  3.356e-11 ***
## ---
## Signif. codes:  0 '***' 0.001 '**' 0.01 '*' 0.05 '.' 0.1 ' ' 1
```

```
# Singular after model diagnostics
CS_2f3 <- lmer(CF_neut_2500~1+emotion*live*IRIEC+emotion*live*AQ+
                 (1+emotion+live|subject)+(1|Type), data = CStbl, REML = TRUE,
               control=lmerControl(optimizer="bobyqa",
                                   optCtrl=list(maxfun=2e8)))

anova(CS_2f3, CS_2f1) # p = 1 *** actually no need to add Type as a random factor
```

```
## refitting model(s) with ML (instead of REML)
```

```
## Data: CStbl
## Models:
## CS_2f1: CF_neut_2500 ~ 1 + emotion * live * IRIEC + emotion * live * AQ + (1 + emotion + live | subject)
## CS_2f3: CF_neut_2500 ~ 1 + emotion * live * IRIEC + emotion * live * AQ + (1 + emotion + live | subject) + (1 | Type)
##        npar     AIC     BIC logLik deviance Chisq Df Pr(>Chisq)
## CS_2f1   19 -3758.6 -3620.1 1898.3  -3796.6                    
## CS_2f3   20 -3756.6 -3610.9 1898.3  -3796.6     0  1          1
```

```
summary(CS_2f3, ddf = "Satterthwaite")
```

```
## Linear mixed model fit by REML. t-tests use Satterthwaite's method [
## lmerModLmerTest]
## Formula: CF_neut_2500 ~ 1 + emotion * live * IRIEC + emotion * live *  
##     AQ + (1 + emotion + live | subject) + (1 | Type)
##    Data: CStbl
## Control: lmerControl(optimizer = "bobyqa", optCtrl = list(maxfun = 2e+08))
## 
## REML criterion at convergence: -3662.6
## 
## Scaled residuals: 
##      Min       1Q   Median       3Q      Max 
## -22.8598  -0.3982  -0.0071   0.3728  15.1416 
## 
## Random effects:
##  Groups   Name        Variance  Std.Dev. Corr     
##  subject  (Intercept) 1.924e-03 0.043861          
##           emotion.L   5.934e-03 0.077033 0.46     
##           live.L      8.369e-04 0.028929 0.66 0.26
##  Type     (Intercept) 2.731e-05 0.005226          
##  Residual             3.972e-02 0.199296          
## Number of obs: 10787, groups:  subject, 93; Type, 2
## 
## Fixed effects:
##                          Estimate Std. Error         df t value Pr(>|t|)    
## (Intercept)            -1.391e-02  6.246e-03  1.346e+00  -2.227 0.214244    
## emotion.L              -3.410e-02  8.567e-03  8.966e+01  -3.981 0.000139 ***
## live.L                 -6.838e-03  4.186e-03  6.373e+01  -1.634 0.107266    
## IRIEC                  -2.791e-04  1.001e-03  8.267e+01  -0.279 0.780964    
## AQ                      3.303e-05  7.460e-04  7.936e+01   0.044 0.964801    
## emotion.L:live.L       -1.566e-02  3.859e-03  1.050e+04  -4.059 4.96e-05 ***
## emotion.L:IRIEC        -1.462e-03  1.693e-03  9.046e+01  -0.863 0.390163    
## live.L:IRIEC           -1.453e-03  8.332e-04  6.729e+01  -1.744 0.085772 .  
## emotion.L:AQ            1.097e-03  1.266e-03  8.973e+01   0.866 0.388641    
## live.L:AQ              -7.852e-04  6.191e-04  6.475e+01  -1.268 0.209200    
## emotion.L:live.L:IRIEC  3.044e-04  7.859e-04  1.050e+04   0.387 0.698507    
## emotion.L:live.L:AQ     3.851e-04  5.767e-04  1.050e+04   0.668 0.504261    
## ---
## Signif. codes:  0 '***' 0.001 '**' 0.01 '*' 0.05 '.' 0.1 ' ' 1
## 
## Correlation of Fixed Effects:
##             (Intr) emtn.L live.L IRIEC  AQ     em.L:.L e.L:IR l.L:IR e.L:AQ
## emotion.L    0.312                                                         
## live.L       0.354  0.171                                                  
## IRIEC        0.005 -0.002  0.004                                           
## AQ          -0.001 -0.004 -0.001  0.208                                    
## emtn.L:lv.L -0.001  0.000 -0.004  0.000  0.000                             
## emt.L:IRIEC -0.002  0.003 -0.001  0.383  0.072  0.000                      
## liv.L:IRIEC -0.003 -0.001  0.039  0.433  0.081  0.000   0.168              
## emotin.L:AQ -0.004 -0.004 -0.002  0.071  0.386  0.000   0.198  0.032       
## live.L:AQ   -0.005 -0.002  0.025  0.081  0.439  0.000   0.032  0.234  0.170
## e.L:.L:IRIE  0.000  0.000  0.000 -0.001  0.001  0.091   0.000 -0.003  0.000
## emt.L:.L:AQ  0.000  0.000  0.000  0.001 -0.002  0.072   0.000  0.000  0.000
##             l.L:AQ e.L:.L:I
## emotion.L                  
## live.L                     
## IRIEC                      
## AQ                         
## emtn.L:lv.L                
## emt.L:IRIEC                
## liv.L:IRIEC                
## emotin.L:AQ                
## live.L:AQ                  
## e.L:.L:IRIE  0.000         
## emt.L:.L:AQ -0.005  0.283
```

```
CS_r2f3 <- rlmer(CF_neut_2500~1+emotion*live*IRIEC+emotion*live*AQ+
                 (1+emotion+live|subject)+(1|Type), data = CStbl, REML = TRUE,
               control=lmerControl(optimizer="bobyqa",
                                   optCtrl=list(maxfun=2e8)))
```

```
## Warning in .rlmerInit(lcall, pf, formula, data, method, rho.e, rho.b,
## rho.sigma.e, : Method 'DAStau' does not support blocks of size larger than 2.
## Falling back to method 'DASvar'.
```

```
summary(CS_r2f3)
```

```
## Robust linear mixed model fit by DASvar 
## Formula: CF_neut_2500 ~ 1 + emotion * live * IRIEC + emotion * live *      AQ + (1 + emotion + live | subject) + (1 | Type) 
##    Data: CStbl 
## Control: lmerControl(optimizer = "bobyqa", optCtrl = list(maxfun = 2e+08)) 
## 
## Scaled residuals: 
##     Min      1Q  Median      3Q     Max 
## -38.336  -0.596   0.004   0.589  24.288 
## 
## Random effects:
##  Groups   Name        Variance  Std.Dev. Corr       
##  subject  (Intercept) 4.201e-04 0.020495            
##           emotion.L   5.191e-04 0.022783  0.02      
##           live.L      5.943e-05 0.007709  0.97 -0.21
##  Type     (Intercept) 6.236e-05 0.007897            
##  Residual             1.591e-02 0.126129            
## Number of obs: 10787, groups: subject, 93; Type, 2
## 
## Fixed effects:
##                          Estimate Std. Error t value
## (Intercept)            -1.125e-02  6.280e-03  -1.791
## emotion.L              -1.527e-02  3.092e-03  -4.938
## live.L                 -1.824e-03  1.983e-03  -0.920
## IRIEC                  -3.759e-05  5.157e-04  -0.073
## AQ                      3.753e-04  3.823e-04   0.982
## emotion.L:live.L       -1.134e-02  2.505e-03  -4.530
## emotion.L:IRIEC        -1.566e-03  6.147e-04  -2.548
## live.L:IRIEC           -3.048e-04  3.971e-04  -0.768
## emotion.L:AQ           -2.850e-04  4.577e-04  -0.623
## live.L:AQ              -1.893e-04  2.925e-04  -0.647
## emotion.L:live.L:IRIEC  5.724e-04  5.101e-04   1.122
## emotion.L:live.L:AQ     5.522e-04  3.743e-04   1.475
## 
## Correlation of Fixed Effects:
##             (Intr) emtn.L live.L IRIEC  AQ     em.L:.L e.L:IR l.L:IR e.L:AQ
## emotion.L    0.005                                                         
## live.L       0.144 -0.075                                                  
## IRIEC        0.003  0.001  0.020                                           
## AQ           0.000  0.001  0.010  0.225                                    
## emtn.L:lv.L -0.001  0.000 -0.006  0.000  0.000                             
## emt.L:IRIEC  0.000  0.029  0.001  0.012  0.003  0.000                      
## liv.L:IRIEC -0.001  0.001  0.074  0.350  0.068  0.000  -0.073              
## emotin.L:AQ  0.000  0.016  0.001  0.003  0.012  0.000   0.224 -0.013       
## live.L:AQ   -0.002  0.001  0.056  0.068  0.360  0.000  -0.013  0.266 -0.076
## e.L:.L:IRIE  0.000  0.000  0.000 -0.002  0.001  0.091   0.000 -0.005  0.000
## emt.L:.L:AQ  0.000  0.000  0.000  0.001 -0.003  0.072   0.000  0.000  0.000
##             l.L:AQ e.L:.L:I
## emotion.L                  
## live.L                     
## IRIEC                      
## AQ                         
## emtn.L:lv.L                
## emt.L:IRIEC                
## liv.L:IRIEC                
## emotin.L:AQ                
## live.L:AQ                  
## e.L:.L:IRIE  0.000         
## emt.L:.L:AQ -0.008  0.283  
## 
## Robustness weights for the residuals: 
##  8344 weights are ~= 1. The remaining 2443 ones are summarized as
##    Min. 1st Qu.  Median    Mean 3rd Qu.    Max. 
##  0.0351  0.5150  0.7290  0.6880  0.8950  0.9990 
## 
## Robustness weights for the random effects: 
##  251 weights are ~= 1. The remaining 30 ones are summarized as
##    Min. 1st Qu.  Median    Mean 3rd Qu.    Max. 
##  0.0131  0.0222  0.1950  0.3020  0.5900  0.9700 
## 
## Rho functions used for fitting:
##   Residuals:
##     eff: smoothed Huber (k = 1.345, s = 10) 
##     sig: smoothed Huber, Proposal 2 (k = 1.345, s = 10) 
##   Random Effects, variance component 1 (subject):
##     eff: smoothed Huber (k = 5.55, s = 10) 
##     vcp: smoothed Huber (k = 5.55, s = 10) 
##   Random Effects, variance component 2 (Type):
##     eff: smoothed Huber (k = 1.345, s = 10) 
##     vcp: smoothed Huber, Proposal 2 (k = 1.345, s = 10)
```

```
coefs_CS_2f3 <- data.frame(coef(summary(CS_2f3)))
coefs_CS_r2f3 <- data.frame(coef(summary(CS_r2f3)))
p_values_CS_r2f3 <- 2 * pt(abs(coefs_CS_r2f3[, "t.value"]), coefs_CS_2f3$df, lower = FALSE)
p_values_CS_r2f3
```

```
##  [1] 2.722136e-01 3.630104e-06 3.611139e-01 9.420586e-01 3.292760e-01
##  [6] 5.976103e-06 1.253174e-02 4.454687e-01 5.350880e-01 5.198432e-01
## [11] 2.618714e-01 1.401680e-01
```

```
# CS_2f3: model diagnostics -> CS_2f11
resid1_CS_2f3 <- hlm_resid(CS_2f3, level = 1, type = "LS", standardize = TRUE)
cooksd1_CS_2f3 <- cooks.distance(CS_2f3)
Q3.cooksd1_CS_2f3 <- quantile(cooksd1_CS_2f3, 0.75, names = TRUE)   
cooksd1.th.CS_2f3 <- Q3.cooksd1_CS_2f3 + 1.5*IQR(cooksd1_CS_2f3)
cooksd2_CS_2f3 <- cooks.distance(CS_2f3, level = "subject")
dotplot_diag(x = cooksd2_CS_2f3, cutoff = "internal",
             name = "cooks.distance") + ylab("Cook's distance") + xlab("trial")
```

```
cooksd2_CS_2f3
```

```
##  [1] 0.0007548721 0.0037694845 0.0037919709 0.0037395899 0.0165307571
##  [6] 0.0004403210 0.0008190674 0.0009659270 0.0058126486 0.0081474456
## [11] 0.0018987787 0.0011833160 0.0280951167 0.0355557061 0.0031992455
## [16] 0.0008912771 0.0627472257 0.0031719563 0.0021785623 0.0011407064
## [21] 0.0002243720 0.0005059310 0.0007402904 0.0022919604 0.0006276311
## [26] 0.0006889542 0.0007095606 0.0046500526 0.0113888720 0.0021739938
## [31] 0.1233607316 0.0108451589 0.0007721649 0.0011813909 0.0049762168
## [36] 0.0002015244 0.0011834805 0.0005753613 0.0001724350 0.0009694489
## [41] 0.0790244961 0.0018610459 0.0011746657 0.0035574624 0.0015408433
## [46] 0.0025311516 0.0349461379 0.0001840431 0.0014407831 0.0016377868
## [51] 0.0042133071 0.0005650968 0.0128120105 0.0039598961 0.0051881119
## [56] 0.0846412880 0.0158225947 0.0005606664 0.0018484472 0.0010591616
## [61] 0.0020868334 0.0039753584 0.0009435345 0.0158061125 0.6458659199
## [66] 0.0072734054 0.0427725569 0.0011615494 0.0008677267 0.0033937329
## [71] 0.0000218250 0.0031098759 0.0038675831 0.0259122917 0.0189262084
## [76] 0.0108572156 0.0029074607 0.0054266153 0.0017616694 0.0083188323
## [81] 0.0033524334 0.0253947660 0.0150798212 0.0010590635 0.0010610865
## [86] 0.0019066285 0.0026910175 0.0064244712 0.0374849318 0.0024326324
## [91] 0.0103250390 0.0014186408 0.0036863050
```

```
# 17, 31, 41, 56, 65 are highly influential subjects
# 89, 47, 67, 14
```

```
CStbl_subject <- unique(CStbl$subject)
CStbl_subject
```

```
##  [1] 1   2   3   4   5   6   7   8   9   10  11  12  13  14  15  16  17  18  19 
## [20] 20  21  22  23  24  25  26  27  28  29  30  31  32  33  34  35  36  37  38 
## [39] 39  40  41  42  43  44  45  46  47  48  49  50  101 102 103 104 105 106 107
## [58] 108 109 110 111 112 113 114 115 116 117 118 119 120 121 122 123 124 125 126
## [77] 127 128 129 130 131 132 133 134 135 136 137 138 139 140 141 142 143
## 93 Levels: 1 2 3 4 5 6 7 8 9 10 11 12 13 14 15 16 17 18 19 20 21 22 23 ... 143
```

```
OLindex.CS_2f3 = which(cooksd1_CS_2f3 > cooksd1.th.CS_2f3 | abs(resid1_CS_2f3$.std.resid) > 3
                       | CStbl$subject == CStbl_subject[14]| CStbl$subject == CStbl_subject[17] 
                       | CStbl$subject == CStbl_subject[31] | CStbl$subject == CStbl_subject[41] 
                       | CStbl$subject == CStbl_subject[47]| CStbl$subject == CStbl_subject[56] 
                       | CStbl$subject == CStbl_subject[65]| CStbl$subject == CStbl_subject[67]
                       | CStbl$subject == CStbl_subject[89])

tbl_CS_2f3 <- CStbl[-OLindex.CS_2f3,]
CS_2f31 <- lmer(CF_neut_2500~1+emotion*live*IRIEC+emotion*live*AQ+
                  (1+emotion+live|subject)+(1|Type), data = tbl_CS_2f3, REML = TRUE,
                control=lmerControl(optimizer="bobyqa",
                                    optCtrl=list(maxfun=2e8)))
summary(CS_2f31)
```

```
## Linear mixed model fit by REML. t-tests use Satterthwaite's method [
## lmerModLmerTest]
## Formula: CF_neut_2500 ~ 1 + emotion * live * IRIEC + emotion * live *  
##     AQ + (1 + emotion + live | subject) + (1 | Type)
##    Data: tbl_CS_2f3
## Control: lmerControl(optimizer = "bobyqa", optCtrl = list(maxfun = 2e+08))
## 
## REML criterion at convergence: -14830.2
## 
## Scaled residuals: 
##     Min      1Q  Median      3Q     Max 
## -4.0765 -0.6139  0.0039  0.6168  4.0715 
## 
## Random effects:
##  Groups   Name        Variance  Std.Dev. Corr     
##  subject  (Intercept) 5.944e-04 0.024381          
##           emotion.L   5.663e-04 0.023797 0.30     
##           live.L      1.093e-04 0.010454 0.50 0.12
##  Type     (Intercept) 9.809e-05 0.009904          
##  Residual             1.047e-02 0.102338          
## Number of obs: 8862, groups:  subject, 84; Type, 2
## 
## Fixed effects:
##                          Estimate Std. Error         df t value Pr(>|t|)    
## (Intercept)            -1.102e-02  7.596e-03  1.016e+00  -1.451   0.3815    
## emotion.L              -1.718e-02  3.125e-03  6.731e+01  -5.496 6.45e-07 ***
## live.L                 -2.984e-03  1.988e-03  7.367e+01  -1.501   0.1376    
## IRIEC                  -1.841e-05  6.217e-04  8.377e+01  -0.030   0.9764    
## AQ                      6.089e-04  4.795e-04  7.982e+01   1.270   0.2078    
## emotion.L:live.L       -7.882e-03  2.179e-03  8.641e+03  -3.617   0.0003 ***
## emotion.L:IRIEC        -1.465e-03  6.671e-04  7.600e+01  -2.196   0.0311 *  
## live.L:IRIEC           -2.334e-05  4.417e-04  9.805e+01  -0.053   0.9580    
## emotion.L:AQ            1.035e-04  5.108e-04  7.033e+01   0.203   0.8400    
## live.L:AQ              -3.860e-04  3.294e-04  8.237e+01  -1.172   0.2446    
## emotion.L:live.L:IRIEC  1.337e-04  5.063e-04  8.665e+03   0.264   0.7918    
## emotion.L:live.L:AQ     3.541e-04  3.690e-04  8.642e+03   0.960   0.3373    
## ---
## Signif. codes:  0 '***' 0.001 '**' 0.01 '*' 0.05 '.' 0.1 ' ' 1
## 
## Correlation of Fixed Effects:
##             (Intr) emtn.L live.L IRIEC  AQ     em.L:.L e.L:IR l.L:IR e.L:AQ
## emotion.L    0.087                                                         
## live.L       0.104  0.059                                                  
## IRIEC       -0.013 -0.007 -0.005                                           
## AQ           0.005  0.004  0.009  0.173                                    
## emtn.L:lv.L -0.001 -0.003 -0.015  0.005  0.001                             
## emt.L:IRIEC -0.003 -0.024  0.003  0.210  0.032 -0.001                      
## liv.L:IRIEC -0.004  0.003  0.002  0.249  0.036 -0.002   0.050              
## emotin.L:AQ  0.001  0.017  0.001  0.033  0.222  0.000   0.182  0.006       
## live.L:AQ    0.001  0.001  0.028  0.038  0.261 -0.002   0.006  0.228  0.057
## e.L:.L:IRIE  0.002 -0.001 -0.001 -0.002 -0.002  0.029  -0.002 -0.022 -0.002
## emt.L:.L:AQ  0.000  0.000 -0.002 -0.002  0.001  0.040  -0.002 -0.006 -0.005
##             l.L:AQ e.L:.L:I
## emotion.L                  
## live.L                     
## IRIEC                      
## AQ                         
## emtn.L:lv.L                
## emt.L:IRIEC                
## liv.L:IRIEC                
## emotin.L:AQ                
## live.L:AQ                  
## e.L:.L:IRIE -0.006         
## emt.L:.L:AQ -0.018  0.271
```

```
r2beta.CS_2f31 <- r2beta(CS_2f31, partial = TRUE, method = "nsj")
r2beta.CS_2f31
```

```
##                    Effect   Rsq upper.CL lower.CL
## 1                   Model 0.018    0.025    0.014
## 2               emotion.L 0.013    0.018    0.008
## 7         emotion.L:IRIEC 0.002    0.004    0.000
## 6        emotion.L:live.L 0.001    0.003    0.000
## 5                      AQ 0.001    0.003    0.000
## 3                  live.L 0.000    0.002    0.000
## 10              live.L:AQ 0.000    0.001    0.000
## 12    emotion.L:live.L:AQ 0.000    0.001    0.000
## 9            emotion.L:AQ 0.000    0.001    0.000
## 11 emotion.L:live.L:IRIEC 0.000    0.001    0.000
## 4                   IRIEC 0.000    0.001    0.000
## 8            live.L:IRIEC 0.000    0.001    0.000
```

```
conf.CS_2f31 <- confint(CS_2f31, oldNames = F)
```

```
## Computing profile confidence intervals ...
```

```
## Warning in nextpar(mat, cc, i, delta, lowcut, upcut): unexpected decrease in
## profile: using minstep
```

```
## Warning in FUN(X[[i]], ...): non-monotonic profile for
## cor_live.L.(Intercept)|subject
```

```
## Warning in zetafun(np, ns): NAs detected in profiling

## Warning in zetafun(np, ns): NAs detected in profiling
```

```
## Warning in nextpar(mat, cc, i, delta, lowcut, upcut): Last two rows have
## identical or NA .zeta values: using minstep
```

```
## Warning in zetafun(np, ns): NAs detected in profiling
```

```
## Warning in nextpar(mat, cc, i, delta, lowcut, upcut): Last two rows have
## identical or NA .zeta values: using minstep
```

```
## Warning in zetafun(np, ns): NAs detected in profiling
```

```
## Warning in nextpar(mat, cc, i, delta, lowcut, upcut): Last two rows have
## identical or NA .zeta values: using minstep
```

```
## Warning in zetafun(np, ns): NAs detected in profiling
```

```
## Warning in nextpar(mat, cc, i, delta, lowcut, upcut): Last two rows have
## identical or NA .zeta values: using minstep
```

```
## Warning in zetafun(np, ns): NAs detected in profiling
```

```
## Warning in nextpar(mat, cc, i, delta, lowcut, upcut): Last two rows have
## identical or NA .zeta values: using minstep
```

```
## Warning in zetafun(np, ns): NAs detected in profiling
```

```
## Warning in nextpar(mat, cc, i, delta, lowcut, upcut): Last two rows have
## identical or NA .zeta values: using minstep
```

```
## Warning in zetafun(np, ns): NAs detected in profiling
```

```
## Warning in nextpar(mat, cc, i, delta, lowcut, upcut): Last two rows have
## identical or NA .zeta values: using minstep
```

```
## Warning in zetafun(np, ns): NAs detected in profiling
```

```
## Warning in nextpar(mat, cc, i, delta, lowcut, upcut): Last two rows have
## identical or NA .zeta values: using minstep
```

```
## Warning in zetafun(np, ns): NAs detected in profiling
```

```
## Warning in nextpar(mat, cc, i, delta, lowcut, upcut): Last two rows have
## identical or NA .zeta values: using minstep
```

```
## Warning in zetafun(np, ns): NAs detected in profiling
```

```
## Warning in nextpar(mat, cc, i, delta, lowcut, upcut): Last two rows have
## identical or NA .zeta values: using minstep
```

```
## Warning in zetafun(np, ns): NAs detected in profiling
```

```
## Warning in nextpar(mat, cc, i, delta, lowcut, upcut): Last two rows have
## identical or NA .zeta values: using minstep
```

```
## Warning in zetafun(np, ns): NAs detected in profiling
```

```
## Warning in nextpar(mat, cc, i, delta, lowcut, upcut): Last two rows have
## identical or NA .zeta values: using minstep
```

```
## Warning in zetafun(np, ns): NAs detected in profiling
```

```
## Warning in nextpar(mat, cc, i, delta, lowcut, upcut): Last two rows have
## identical or NA .zeta values: using minstep
```

```
## Warning in zetafun(np, ns): NAs detected in profiling
```

```
## Warning in nextpar(mat, cc, i, delta, lowcut, upcut): Last two rows have
## identical or NA .zeta values: using minstep
```

```
## Warning in zetafun(np, ns): NAs detected in profiling
```

```
## Warning in nextpar(mat, cc, i, delta, lowcut, upcut): Last two rows have
## identical or NA .zeta values: using minstep
```

```
## Warning in zetafun(np, ns): NAs detected in profiling
```

```
## Warning in nextpar(mat, cc, i, delta, lowcut, upcut): Last two rows have
## identical or NA .zeta values: using minstep
```

```
## Warning in zetafun(np, ns): NAs detected in profiling
```

```
## Warning in nextpar(mat, cc, i, delta, lowcut, upcut): Last two rows have
## identical or NA .zeta values: using minstep
```

```
## Warning in zetafun(np, ns): NAs detected in profiling
```

```
## Warning in nextpar(mat, cc, i, delta, lowcut, upcut): Last two rows have
## identical or NA .zeta values: using minstep
```

```
## Warning in zetafun(np, ns): NAs detected in profiling
```

```
## Warning in nextpar(mat, cc, i, delta, lowcut, upcut): Last two rows have
## identical or NA .zeta values: using minstep
```

```
## Warning in zetafun(np, ns): NAs detected in profiling
```

```
## Warning in nextpar(mat, cc, i, delta, lowcut, upcut): Last two rows have
## identical or NA .zeta values: using minstep
```

```
## Warning in zetafun(np, ns): NAs detected in profiling
```

```
## Warning in nextpar(mat, cc, i, delta, lowcut, upcut): Last two rows have
## identical or NA .zeta values: using minstep
```

```
## Warning in zetafun(np, ns): NAs detected in profiling
```

```
## Warning in nextpar(mat, cc, i, delta, lowcut, upcut): Last two rows have
## identical or NA .zeta values: using minstep
```

```
## Warning in zetafun(np, ns): NAs detected in profiling
```

```
## Warning in nextpar(mat, cc, i, delta, lowcut, upcut): Last two rows have
## identical or NA .zeta values: using minstep
```

```
## Warning in zetafun(np, ns): NAs detected in profiling
```

```
## Warning in nextpar(mat, cc, i, delta, lowcut, upcut): Last two rows have
## identical or NA .zeta values: using minstep
```

```
## Warning in zetafun(np, ns): NAs detected in profiling
```

```
## Warning in nextpar(mat, cc, i, delta, lowcut, upcut): Last two rows have
## identical or NA .zeta values: using minstep
```

```
## Warning in zetafun(np, ns): NAs detected in profiling
```

```
## Warning in nextpar(mat, cc, i, delta, lowcut, upcut): Last two rows have
## identical or NA .zeta values: using minstep
```

```
## Warning in zetafun(np, ns): NAs detected in profiling
```

```
## Warning in nextpar(mat, cc, i, delta, lowcut, upcut): Last two rows have
## identical or NA .zeta values: using minstep
```

```
## Warning in zetafun(np, ns): NAs detected in profiling
```

```
## Warning in nextpar(mat, cc, i, delta, lowcut, upcut): Last two rows have
## identical or NA .zeta values: using minstep
```

```
## Warning in zetafun(np, ns): NAs detected in profiling
```

```
## Warning in nextpar(mat, cc, i, delta, lowcut, upcut): Last two rows have
## identical or NA .zeta values: using minstep
```

```
## Warning in zetafun(np, ns): NAs detected in profiling
```

```
## Warning in nextpar(mat, cc, i, delta, lowcut, upcut): Last two rows have
## identical or NA .zeta values: using minstep
```

```
## Warning in zetafun(np, ns): NAs detected in profiling
```

```
## Warning in nextpar(mat, cc, i, delta, lowcut, upcut): Last two rows have
## identical or NA .zeta values: using minstep
```

```
## Warning in zetafun(np, ns): NAs detected in profiling
```

```
## Warning in nextpar(mat, cc, i, delta, lowcut, upcut): Last two rows have
## identical or NA .zeta values: using minstep
```

```
## Warning in zetafun(np, ns): NAs detected in profiling
```

```
## Warning in nextpar(mat, cc, i, delta, lowcut, upcut): Last two rows have
## identical or NA .zeta values: using minstep
```

```
## Warning in zetafun(np, ns): NAs detected in profiling
```

```
## Warning in nextpar(mat, cc, i, delta, lowcut, upcut): Last two rows have
## identical or NA .zeta values: using minstep
```

```
## Warning in zetafun(np, ns): NAs detected in profiling
```

```
## Warning in nextpar(mat, cc, i, delta, lowcut, upcut): Last two rows have
## identical or NA .zeta values: using minstep
```

```
## Warning in zetafun(np, ns): NAs detected in profiling
```

```
## Warning in nextpar(mat, cc, i, delta, lowcut, upcut): Last two rows have
## identical or NA .zeta values: using minstep
```

```
## Warning in zetafun(np, ns): NAs detected in profiling
```

```
## Warning in nextpar(mat, cc, i, delta, lowcut, upcut): Last two rows have
## identical or NA .zeta values: using minstep
```

```
## Warning in zetafun(np, ns): NAs detected in profiling
```

```
## Warning in nextpar(mat, cc, i, delta, lowcut, upcut): Last two rows have
## identical or NA .zeta values: using minstep
```

```
## Warning in zetafun(np, ns): NAs detected in profiling
```

```
## Warning in nextpar(mat, cc, i, delta, lowcut, upcut): Last two rows have
## identical or NA .zeta values: using minstep
```

```
## Warning in zetafun(np, ns): NAs detected in profiling
```

```
## Warning in nextpar(mat, cc, i, delta, lowcut, upcut): Last two rows have
## identical or NA .zeta values: using minstep
```

```
## Warning in zetafun(np, ns): NAs detected in profiling
```

```
## Warning in nextpar(mat, cc, i, delta, lowcut, upcut): Last two rows have
## identical or NA .zeta values: using minstep
```

```
## Warning in zetafun(np, ns): NAs detected in profiling
```

```
## Warning in nextpar(mat, cc, i, delta, lowcut, upcut): Last two rows have
## identical or NA .zeta values: using minstep
```

```
## Warning in zetafun(np, ns): NAs detected in profiling
```

```
## Warning in nextpar(mat, cc, i, delta, lowcut, upcut): Last two rows have
## identical or NA .zeta values: using minstep
```

```
## Warning in zetafun(np, ns): NAs detected in profiling
```

```
## Warning in nextpar(mat, cc, i, delta, lowcut, upcut): Last two rows have
## identical or NA .zeta values: using minstep
```

```
## Warning in zetafun(np, ns): NAs detected in profiling
```

```
## Warning in nextpar(mat, cc, i, delta, lowcut, upcut): Last two rows have
## identical or NA .zeta values: using minstep
```

```
## Warning in zetafun(np, ns): NAs detected in profiling
```

```
## Warning in nextpar(mat, cc, i, delta, lowcut, upcut): Last two rows have
## identical or NA .zeta values: using minstep
```

```
## Warning in zetafun(np, ns): NAs detected in profiling
```

```
## Warning in nextpar(mat, cc, i, delta, lowcut, upcut): Last two rows have
## identical or NA .zeta values: using minstep
```

```
## Warning in zetafun(np, ns): NAs detected in profiling
```

```
## Warning in nextpar(mat, cc, i, delta, lowcut, upcut): Last two rows have
## identical or NA .zeta values: using minstep
```

```
## Warning in zetafun(np, ns): NAs detected in profiling
```

```
## Warning in nextpar(mat, cc, i, delta, lowcut, upcut): Last two rows have
## identical or NA .zeta values: using minstep
```

```
## Warning in zetafun(np, ns): NAs detected in profiling
```

```
## Warning in nextpar(mat, cc, i, delta, lowcut, upcut): Last two rows have
## identical or NA .zeta values: using minstep
```

```
## Warning in zetafun(np, ns): NAs detected in profiling
```

```
## Warning in nextpar(mat, cc, i, delta, lowcut, upcut): Last two rows have
## identical or NA .zeta values: using minstep
```

```
## Warning in zetafun(np, ns): NAs detected in profiling
```

```
## Warning in nextpar(mat, cc, i, delta, lowcut, upcut): Last two rows have
## identical or NA .zeta values: using minstep
```

```
## Warning in zetafun(np, ns): NAs detected in profiling
```

```
## Warning in nextpar(mat, cc, i, delta, lowcut, upcut): Last two rows have
## identical or NA .zeta values: using minstep
```

```
## Warning in zetafun(np, ns): NAs detected in profiling
```

```
## Warning in nextpar(mat, cc, i, delta, lowcut, upcut): Last two rows have
## identical or NA .zeta values: using minstep
```

```
## Warning in zetafun(np, ns): NAs detected in profiling
```

```
## Warning in nextpar(mat, cc, i, delta, lowcut, upcut): Last two rows have
## identical or NA .zeta values: using minstep
```

```
## Warning in zetafun(np, ns): NAs detected in profiling
```

```
## Warning in nextpar(mat, cc, i, delta, lowcut, upcut): Last two rows have
## identical or NA .zeta values: using minstep
```

```
## Warning in zetafun(np, ns): NAs detected in profiling
```

```
## Warning in nextpar(mat, cc, i, delta, lowcut, upcut): Last two rows have
## identical or NA .zeta values: using minstep
```

```
## Warning in zetafun(np, ns): NAs detected in profiling
```

```
## Warning in nextpar(mat, cc, i, delta, lowcut, upcut): Last two rows have
## identical or NA .zeta values: using minstep
```

```
## Warning in zetafun(np, ns): NAs detected in profiling
```

```
## Warning in nextpar(mat, cc, i, delta, lowcut, upcut): Last two rows have
## identical or NA .zeta values: using minstep
```

```
## Warning in zetafun(np, ns): NAs detected in profiling
```

```
## Warning in nextpar(mat, cc, i, delta, lowcut, upcut): Last two rows have
## identical or NA .zeta values: using minstep
```

```
## Warning in zetafun(np, ns): NAs detected in profiling
```

```
## Warning in nextpar(mat, cc, i, delta, lowcut, upcut): Last two rows have
## identical or NA .zeta values: using minstep
```

```
## Warning in zetafun(np, ns): NAs detected in profiling
```

```
## Warning in nextpar(mat, cc, i, delta, lowcut, upcut): Last two rows have
## identical or NA .zeta values: using minstep
```

```
## Warning in zetafun(np, ns): NAs detected in profiling
```

```
## Warning in nextpar(mat, cc, i, delta, lowcut, upcut): Last two rows have
## identical or NA .zeta values: using minstep
```

```
## Warning in zetafun(np, ns): NAs detected in profiling
```

```
## Warning in nextpar(mat, cc, i, delta, lowcut, upcut): Last two rows have
## identical or NA .zeta values: using minstep
```

```
## Warning in zetafun(np, ns): NAs detected in profiling
```

```
## Warning in nextpar(mat, cc, i, delta, lowcut, upcut): Last two rows have
## identical or NA .zeta values: using minstep
```

```
## Warning in zetafun(np, ns): NAs detected in profiling
```

```
## Warning in nextpar(mat, cc, i, delta, lowcut, upcut): Last two rows have
## identical or NA .zeta values: using minstep
```

```
## Warning in zetafun(np, ns): NAs detected in profiling
```

```
## Warning in nextpar(mat, cc, i, delta, lowcut, upcut): Last two rows have
## identical or NA .zeta values: using minstep
```

```
## Warning in zetafun(np, ns): NAs detected in profiling
```

```
## Warning in nextpar(mat, cc, i, delta, lowcut, upcut): Last two rows have
## identical or NA .zeta values: using minstep
```

```
## Warning in zetafun(np, ns): NAs detected in profiling
```

```
## Warning in nextpar(mat, cc, i, delta, lowcut, upcut): Last two rows have
## identical or NA .zeta values: using minstep
```

```
## Warning in zetafun(np, ns): NAs detected in profiling
```

```
## Warning in nextpar(mat, cc, i, delta, lowcut, upcut): Last two rows have
## identical or NA .zeta values: using minstep
```

```
## Warning in zetafun(np, ns): NAs detected in profiling
```

```
## Warning in nextpar(mat, cc, i, delta, lowcut, upcut): Last two rows have
## identical or NA .zeta values: using minstep
```

```
## Warning in zetafun(np, ns): NAs detected in profiling
```

```
## Warning in nextpar(mat, cc, i, delta, lowcut, upcut): Last two rows have
## identical or NA .zeta values: using minstep
```

```
## Warning in zetafun(np, ns): NAs detected in profiling
```

```
## Warning in nextpar(mat, cc, i, delta, lowcut, upcut): Last two rows have
## identical or NA .zeta values: using minstep
```

```
## Warning in zetafun(np, ns): NAs detected in profiling
```

```
## Warning in nextpar(mat, cc, i, delta, lowcut, upcut): Last two rows have
## identical or NA .zeta values: using minstep
```

```
## Warning in zetafun(np, ns): NAs detected in profiling
```

```
## Warning in nextpar(mat, cc, i, delta, lowcut, upcut): Last two rows have
## identical or NA .zeta values: using minstep
```

```
## Warning in zetafun(np, ns): NAs detected in profiling
```

```
## Warning in nextpar(mat, cc, i, delta, lowcut, upcut): Last two rows have
## identical or NA .zeta values: using minstep
```

```
## Warning in zetafun(np, ns): NAs detected in profiling
```

```
## Warning in nextpar(mat, cc, i, delta, lowcut, upcut): Last two rows have
## identical or NA .zeta values: using minstep
```

```
## Warning in zetafun(np, ns): NAs detected in profiling
```

```
## Warning in nextpar(mat, cc, i, delta, lowcut, upcut): Last two rows have
## identical or NA .zeta values: using minstep
```

```
## Warning in zetafun(np, ns): NAs detected in profiling
```

```
## Warning in nextpar(mat, cc, i, delta, lowcut, upcut): Last two rows have
## identical or NA .zeta values: using minstep
```

```
## Warning in zetafun(np, ns): NAs detected in profiling
```

```
## Warning in nextpar(mat, cc, i, delta, lowcut, upcut): Last two rows have
## identical or NA .zeta values: using minstep
```

```
## Warning in zetafun(np, ns): NAs detected in profiling
```

```
## Warning in nextpar(mat, cc, i, delta, lowcut, upcut): Last two rows have
## identical or NA .zeta values: using minstep
```

```
## Warning in zetafun(np, ns): NAs detected in profiling
```

```
## Warning in nextpar(mat, cc, i, delta, lowcut, upcut): Last two rows have
## identical or NA .zeta values: using minstep
```

```
## Warning in zetafun(np, ns): NAs detected in profiling
```

```
## Warning in nextpar(mat, cc, i, delta, lowcut, upcut): Last two rows have
## identical or NA .zeta values: using minstep
```

```
## Warning in zetafun(np, ns): NAs detected in profiling
```

```
## Warning in optwrap(optimizer, par = thopt, fn = mkdevfun(rho, 0L), lower =
## fitted@lower): convergence code 1 from bobyqa: bobyqa -- maximum number of
## function evaluations exceeded

## Warning in optwrap(optimizer, par = thopt, fn = mkdevfun(rho, 0L), lower =
## fitted@lower): convergence code 1 from bobyqa: bobyqa -- maximum number of
## function evaluations exceeded

## Warning in optwrap(optimizer, par = thopt, fn = mkdevfun(rho, 0L), lower =
## fitted@lower): convergence code 1 from bobyqa: bobyqa -- maximum number of
## function evaluations exceeded

## Warning in optwrap(optimizer, par = thopt, fn = mkdevfun(rho, 0L), lower =
## fitted@lower): convergence code 1 from bobyqa: bobyqa -- maximum number of
## function evaluations exceeded

## Warning in optwrap(optimizer, par = thopt, fn = mkdevfun(rho, 0L), lower =
## fitted@lower): convergence code 1 from bobyqa: bobyqa -- maximum number of
## function evaluations exceeded
```

```
## Warning in cov2sdcor(tcrossprod(m) * s^2): NA values in sdcor matrix converted
## to 0
```

```
## Warning in confint.thpr(pp, level = level, zeta = zeta): bad spline fit for
## cor_live.L.(Intercept)|subject: falling back to linear interpolation
```

```
conf.CS_2f31
```

```
##                                           2.5 %        97.5 %
## sd_(Intercept)|subject             0.0199896806  0.0292298438
## cor_emotion.L.(Intercept)|subject  0.0209179723  0.5606067411
## cor_live.L.(Intercept)|subject     0.1312059185  0.8594267870
## sd_emotion.L|subject               0.0180258596  0.0291485720
## cor_live.L.emotion.L|subject      -0.3704996230  0.2802165451
## sd_live.L|subject                  0.0053462390  0.0145447722
## sd_(Intercept)|Type                0.0000000000  0.0311764792
## sigma                              0.1008118582  0.1038666317
## (Intercept)                       -0.0291723879  0.0070982895
## emotion.L                         -0.0233358507 -0.0111414291
## live.L                            -0.0069753234  0.0008475370
## IRIEC                             -0.0012632855  0.0011813826
## AQ                                -0.0003468844  0.0015376383
## emotion.L:live.L                  -0.0121575105 -0.0036156599
## emotion.L:IRIEC                   -0.0027622521 -0.0001675139
## live.L:IRIEC                      -0.0008851970  0.0008383735
## emotion.L:AQ                      -0.0008887395  0.0010983448
## live.L:AQ                         -0.0010273086  0.0002565883
## emotion.L:live.L:IRIEC            -0.0008580442  0.0011265654
## emotion.L:live.L:AQ               -0.0003686664  0.0010777063
```

```
CS_2f31.em1 <- emmeans(CS_2f31, list(pairwise ~ emotion|live), adjust = "tukey")
```

```
## Note: D.f. calculations have been disabled because the number of observations exceeds 3000.
## To enable adjustments, add the argument 'pbkrtest.limit = 8862' (or larger)
## [or, globally, 'set emm_options(pbkrtest.limit = 8862)' or larger];
## but be warned that this may result in large computation time and memory use.
```

```
## NOTE: Results may be misleading due to involvement in interactions
```

```
CS_2f31.em1
```

```
## $`emmeans of emotion | live`
## live = Video:
##  emotion     emmean      SE   df lower.CL upper.CL
##  Negative -0.000998 0.00781 1.13  -0.0770   0.0750
##  Positive -0.017183 0.00813 1.34  -0.0754   0.0411
## 
## live = Live:
##  emotion     emmean      SE   df lower.CL upper.CL
##  Negative  0.002860 0.00805 1.27  -0.0597   0.0654
##  Positive -0.029254 0.00844 1.55  -0.0777   0.0192
## 
## Degrees-of-freedom method: satterthwaite 
## Confidence level used: 0.95 
## 
## $`pairwise differences of emotion | live`
## live = Video:
##  2                   estimate      SE  df t.ratio p.value
##  Negative - Positive   0.0162 0.00494 104   3.279  0.0014
## 
## live = Live:
##  2                   estimate      SE  df t.ratio p.value
##  Negative - Positive   0.0321 0.00492 103   6.523  <.0001
## 
## Degrees-of-freedom method: satterthwaite
```

```
CS_2f31.em11 <- emmeans(CS_2f31, list(pairwise ~ live|emotion), adjust = "tukey")
```

```
## Note: D.f. calculations have been disabled because the number of observations exceeds 3000.
## To enable adjustments, add the argument 'pbkrtest.limit = 8862' (or larger)
## [or, globally, 'set emm_options(pbkrtest.limit = 8862)' or larger];
## but be warned that this may result in large computation time and memory use.
## NOTE: Results may be misleading due to involvement in interactions
```

```
CS_2f31.em11
```

```
## $`emmeans of live | emotion`
## emotion = Negative:
##  live     emmean      SE   df lower.CL upper.CL
##  Video -0.000998 0.00781 1.13  -0.0770   0.0750
##  Live   0.002860 0.00805 1.27  -0.0597   0.0654
## 
## emotion = Positive:
##  live     emmean      SE   df lower.CL upper.CL
##  Video -0.017183 0.00813 1.34  -0.0754   0.0411
##  Live  -0.029254 0.00844 1.55  -0.0777   0.0192
## 
## Degrees-of-freedom method: satterthwaite 
## Confidence level used: 0.95 
## 
## $`pairwise differences of live | emotion`
## emotion = Negative:
##  2            estimate      SE  df t.ratio p.value
##  Video - Live -0.00386 0.00358 193  -1.077  0.2828
## 
## emotion = Positive:
##  2            estimate      SE  df t.ratio p.value
##  Video - Live  0.01207 0.00353 181   3.420  0.0008
## 
## Degrees-of-freedom method: satterthwaite
```

```
CS_2f31.em2 <- emtrends(CS_2f31, list(pairwise ~ emotion), var = "IRIEC", adjust = "tukey")
```

```
## Note: D.f. calculations have been disabled because the number of observations exceeds 3000.
## To enable adjustments, add the argument 'pbkrtest.limit = 8862' (or larger)
## [or, globally, 'set emm_options(pbkrtest.limit = 8862)' or larger];
## but be warned that this may result in large computation time and memory use.
## NOTE: Results may be misleading due to involvement in interactions
```

```
CS_2f31.em2
```

```
## $`emmeans of emotion`
##  emotion  IRIEC.trend       SE   df  lower.CL upper.CL
##  Negative     0.00102 0.000697 87.0 -0.000368 0.002403
##  Positive    -0.00105 0.000856 78.3 -0.002758 0.000649
## 
## Results are averaged over the levels of: live 
## Degrees-of-freedom method: satterthwaite 
## Confidence level used: 0.95 
## 
## $`pairwise differences of emotion`
##  1                   estimate       SE df t.ratio p.value
##  Negative - Positive  0.00207 0.000943 76   2.196  0.0311
## 
## Results are averaged over the levels of: live 
## Degrees-of-freedom method: satterthwaite
```

```
CS_2f31.em22 <- emmeans(CS_2f31,  ~ emotion*IRIEC, at=list(IRIEC=c(-15.504, -10.336, -5.168, 0, 5.168, 10.336, 15.504), emotion = c("Positive","Negative")), adjust = "sidak")
```

```
## Note: D.f. calculations have been disabled because the number of observations exceeds 3000.
## To enable adjustments, add the argument 'pbkrtest.limit = 8862' (or larger)
## [or, globally, 'set emm_options(pbkrtest.limit = 8862)' or larger];
## but be warned that this may result in large computation time and memory use.
## NOTE: Results may be misleading due to involvement in interactions
```

```
CS_2f31.em22
```

```
##  emotion   IRIEC   emmean      SE    df lower.CL upper.CL
##  Positive -15.50 -0.00696 0.01567 16.93  -0.0598   0.0458
##  Negative -15.50 -0.01476 0.01337  8.92  -0.0670   0.0375
##  Positive -10.34 -0.01241 0.01210  6.50  -0.0660   0.0411
##  Negative -10.34 -0.00950 0.01064  3.78  -0.0778   0.0588
##  Positive  -5.17 -0.01786 0.00929  2.29  -0.1348   0.0991
##  Negative  -5.17 -0.00424 0.00857  1.63  -0.2388   0.2304
##  Positive   0.00 -0.02331 0.00809  1.31  -0.4631   0.4165
##  Negative   0.00  0.00102 0.00772  1.08  -0.9280   0.9300
##  Positive   5.17 -0.02875 0.00915  2.15  -0.1583   0.1008
##  Negative   5.17  0.00627 0.00847  1.56  -0.2562   0.2687
##  Positive  10.34 -0.03420 0.01188  6.06  -0.0887   0.0203
##  Negative  10.34  0.01153 0.01048  3.58  -0.0594   0.0825
##  Positive  15.50 -0.03965 0.01541 16.02  -0.0921   0.0128
##  Negative  15.50  0.01679 0.01319  8.51  -0.0355   0.0691
## 
## Results are averaged over the levels of: live 
## Degrees-of-freedom method: satterthwaite 
## Confidence level used: 0.95 
## Conf-level adjustment: sidak method for 14 estimates
```

```
contrast(CS_2f31.em22, "pairwise", by="IRIEC")
```

```
## IRIEC = -15.50:
##  contrast            estimate      SE   df t.ratio p.value
##  Positive - Negative  0.00780 0.01541 73.8   0.506  0.6143
## 
## IRIEC = -10.34:
##  contrast            estimate      SE   df t.ratio p.value
##  Positive - Negative -0.00291 0.01083 72.5  -0.269  0.7889
## 
## IRIEC =  -5.17:
##  contrast            estimate      SE   df t.ratio p.value
##  Positive - Negative -0.01361 0.00668 69.6  -2.039  0.0453
## 
## IRIEC =   0.00:
##  contrast            estimate      SE   df t.ratio p.value
##  Positive - Negative -0.02432 0.00442 67.3  -5.503  <.0001
## 
## IRIEC =   5.17:
##  contrast            estimate      SE   df t.ratio p.value
##  Positive - Negative -0.03503 0.00648 74.4  -5.404  <.0001
## 
## IRIEC =  10.34:
##  contrast            estimate      SE   df t.ratio p.value
##  Positive - Negative -0.04573 0.01058 76.5  -4.321  <.0001
## 
## IRIEC =  15.50:
##  contrast            estimate      SE   df t.ratio p.value
##  Positive - Negative -0.05644 0.01515 76.7  -3.725  0.0004
## 
## Results are averaged over the levels of: live 
## Degrees-of-freedom method: satterthwaite
```

```
CS_2f31.predict2 <- ggemmeans(CS_2f31, terms = c("IRIEC", "emotion"))
```

```
## NOTE: Results may be misleading due to involvement in interactions
```

```
CS_2f31_IRIECemo <- plot(CS_2f31.predict2, colors = c("steelblue","red"), show.title = F) +
  labs(x = "Mean-Centered IRIEC", y = "Corrugator Responses", colour = "Emotion") +
  guides(colour = guide_legend(reverse = T)) +
  theme(title = element_text(size = 8, face = "bold"), axis.title.x = element_text(size = 8, face = "bold"), 
        axis.title.y = element_text(size = 8, face = "bold"), strip.text = element_text(size = 8, face = "bold"),
        legend.title = element_text(size = 6, face = "bold"), legend.text = element_text(size = 6), 
        axis.text = element_text(size = 6))
CS_2f31_IRIECemo # Fig
```

```
Fig4 <- plot_grid(val_2f2.IRIECemo, aro_2f1.IRIECemo, CS_2f31_IRIECemo, rel_widths = c(1, 1, 1.5), labels = 'AUTO',
                      ncol = 3, nrow = 1, align = "h")
Fig4
```

```
ggsave(Fig4, filename='Fig4_2f94.pdf', bg = "white", dpi=300, scale = 1, width = 19.05, height = 9.55, unit = "cm")
```

```
CS50_2f0 <- lmer(CF_neut_2500~1+emotion*live*IRIEC+emotion*live*AQ+
                 (1+emotion|subject), data = CStbl50, REML = TRUE,
               control=lmerControl(optimizer="bobyqa",
                                   optCtrl=list(maxfun=2e8)))
CS50_2f1 <- lmer(CF_neut_2500~1+emotion*live*IRIEC+emotion*live*AQ+
                 (1+emotion+live|subject), data = CStbl50, REML = TRUE,
               control=lmerControl(optimizer="bobyqa",
                                   optCtrl=list(maxfun=2e8)))
```

```
## boundary (singular) fit: see help('isSingular')
```

```
#Singular
anova(CS50_2f0, CS50_2f1)
```

```
## refitting model(s) with ML (instead of REML)
```

```
## Data: CStbl50
## Models:
## CS50_2f0: CF_neut_2500 ~ 1 + emotion * live * IRIEC + emotion * live * AQ + (1 + emotion | subject)
## CS50_2f1: CF_neut_2500 ~ 1 + emotion * live * IRIEC + emotion * live * AQ + (1 + emotion + live | subject)
##          npar     AIC    BIC logLik deviance  Chisq Df Pr(>Chisq)    
## CS50_2f0   16 -44.307 51.773 38.154  -76.307                         
## CS50_2f1   19 -73.241 40.855 55.620 -111.241 34.933  3  1.258e-07 ***
## ---
## Signif. codes:  0 '***' 0.001 '**' 0.01 '*' 0.05 '.' 0.1 ' ' 1
```

```
summary(CS50_2f1, ddf = "Satterthwaite")
```

```
## Linear mixed model fit by REML. t-tests use Satterthwaite's method [
## lmerModLmerTest]
## Formula: CF_neut_2500 ~ 1 + emotion * live * IRIEC + emotion * live *  
##     AQ + (1 + emotion + live | subject)
##    Data: CStbl50
## Control: lmerControl(optimizer = "bobyqa", optCtrl = list(maxfun = 2e+08))
## 
## REML criterion at convergence: 8.7
## 
## Scaled residuals: 
##      Min       1Q   Median       3Q      Max 
## -19.4888  -0.2629   0.0034   0.2642  13.0547 
## 
## Random effects:
##  Groups   Name        Variance Std.Dev. Corr     
##  subject  (Intercept) 0.002467 0.04967           
##           emotion.L   0.005722 0.07565  0.27     
##           live.L      0.001656 0.04070  0.98 0.45
##  Residual             0.053962 0.23230           
## Number of obs: 2996, groups:  subject, 50
## 
## Fixed effects:
##                          Estimate Std. Error         df t value Pr(>|t|)   
## (Intercept)            -2.458e-02  8.331e-03  4.755e+01  -2.950  0.00492 **
## emotion.L              -4.316e-02  1.245e-02  4.704e+01  -3.466  0.00114 **
## live.L                 -1.680e-02  8.441e-03  5.787e+01  -1.991  0.05125 . 
## IRIEC                  -8.559e-04  1.652e-03  4.754e+01  -0.518  0.60675   
## AQ                     -6.147e-04  1.270e-03  4.761e+01  -0.484  0.63059   
## emotion.L:live.L       -2.322e-02  8.616e-03  2.890e+03  -2.695  0.00709 **
## emotion.L:IRIEC        -2.814e-03  2.469e-03  4.703e+01  -1.140  0.26018   
## live.L:IRIEC           -1.767e-03  1.674e-03  5.785e+01  -1.056  0.29542   
## emotion.L:AQ           -6.375e-04  1.898e-03  4.709e+01  -0.336  0.73847   
## live.L:AQ              -8.484e-04  1.287e-03  5.800e+01  -0.659  0.51239   
## emotion.L:live.L:IRIEC -1.164e-03  1.708e-03  2.890e+03  -0.681  0.49565   
## emotion.L:live.L:AQ    -1.764e-03  1.315e-03  2.890e+03  -1.342  0.17976   
## ---
## Signif. codes:  0 '***' 0.001 '**' 0.01 '*' 0.05 '.' 0.1 ' ' 1
## 
## Correlation of Fixed Effects:
##             (Intr) emtn.L live.L IRIEC  AQ     em.L:.L e.L:IR l.L:IR e.L:AQ
## emotion.L    0.200                                                         
## live.L       0.581  0.273                                                  
## IRIEC       -0.148 -0.030 -0.086                                           
## AQ          -0.085 -0.017 -0.050 -0.016                                    
## emtn.L:lv.L  0.000  0.000 -0.001  0.000  0.001                             
## emt.L:IRIEC -0.030 -0.148 -0.040  0.200 -0.003  0.000                      
## liv.L:IRIEC -0.086 -0.040 -0.148  0.581 -0.010  0.000   0.273              
## emotin.L:AQ -0.017 -0.085 -0.022 -0.003  0.199 -0.001  -0.016 -0.004       
## live.L:AQ   -0.050 -0.022 -0.085 -0.010  0.581  0.001  -0.004 -0.017  0.272
## e.L:.L:IRIE  0.000  0.000  0.000 -0.001  0.001 -0.148   0.001 -0.001 -0.001
## emt.L:.L:AQ  0.001 -0.001  0.001  0.001 -0.001 -0.085  -0.001  0.001  0.001
##             l.L:AQ e.L:.L:I
## emotion.L                  
## live.L                     
## IRIEC                      
## AQ                         
## emtn.L:lv.L                
## emt.L:IRIEC                
## liv.L:IRIEC                
## emotin.L:AQ                
## live.L:AQ                  
## e.L:.L:IRIE  0.001         
## emt.L:.L:AQ -0.002 -0.017  
## optimizer (bobyqa) convergence code: 0 (OK)
## boundary (singular) fit: see help('isSingular')
```

```
CS50_r2f1 <- rlmer(CF_neut_2500~1+emotion*live*IRIEC+emotion*live*AQ+
                 (1+emotion+live|subject), data = CStbl50, REML = TRUE,
               control=lmerControl(optimizer="bobyqa",
                                   optCtrl=list(maxfun=2e8)))
```

```
## boundary (singular) fit: see help('isSingular')
```

```
## Warning in .rlmerInit(lcall, pf, formula, data, method, rho.e, rho.b,
## rho.sigma.e, : Method 'DAStau' does not support blocks of size larger than 2.
## Falling back to method 'DASvar'.
```

```
summary(CS50_r2f1)
```

```
## Robust linear mixed model fit by DASvar 
## Formula: CF_neut_2500 ~ 1 + emotion * live * IRIEC + emotion * live *      AQ + (1 + emotion + live | subject) 
##    Data: CStbl50 
## Control: lmerControl(optimizer = "bobyqa", optCtrl = list(maxfun = 2e+08)) 
## 
## Scaled residuals: 
##     Min      1Q  Median      3Q     Max 
## -48.475  -0.577   0.005   0.571  30.855 
## 
## Random effects:
##  Groups   Name        Variance  Std.Dev. Corr       
##  subject  (Intercept) 0.0006739 0.02596             
##           emotion.L   0.0012539 0.03541   0.00      
##           live.L      0.0001787 0.01337   1.00 -0.01
##  Residual             0.0098919 0.09946             
## Number of obs: 2996, groups: subject, 50
## 
## Fixed effects:
##                          Estimate Std. Error t value
## (Intercept)            -0.0196968  0.0042405  -4.645
## emotion.L              -0.0228952  0.0058274  -3.929
## live.L                 -0.0068986  0.0033132  -2.082
## IRIEC                  -0.0006767  0.0008408  -0.805
## AQ                      0.0004076  0.0006463   0.631
## emotion.L:live.L       -0.0142490  0.0037837  -3.766
## emotion.L:IRIEC        -0.0014414  0.0011554  -1.248
## live.L:IRIEC           -0.0003439  0.0006569  -0.524
## emotion.L:AQ           -0.0006011  0.0008882  -0.677
## live.L:AQ              -0.0001097  0.0005053  -0.217
## emotion.L:live.L:IRIEC -0.0004271  0.0007501  -0.569
## emotion.L:live.L:AQ    -0.0001997  0.0005773  -0.346
## 
## Correlation of Fixed Effects:
##             (Intr) emtn.L live.L IRIEC  AQ     em.L:.L e.L:IR l.L:IR e.L:AQ
## emotion.L    0.001                                                         
## live.L       0.528 -0.008                                                  
## IRIEC       -0.148  0.000 -0.078                                           
## AQ          -0.085  0.000 -0.045 -0.016                                    
## emtn.L:lv.L  0.000  0.000 -0.001  0.000  0.001                             
## emt.L:IRIEC  0.000 -0.148  0.001  0.001  0.000  0.000                      
## liv.L:IRIEC -0.078  0.001 -0.148  0.528 -0.009  0.000  -0.008              
## emotin.L:AQ  0.000 -0.085  0.001  0.000  0.001 -0.001  -0.016  0.001       
## live.L:AQ   -0.045  0.001 -0.085 -0.009  0.528  0.001   0.001 -0.017 -0.008
## e.L:.L:IRIE  0.000  0.000  0.000  0.000  0.001 -0.148   0.000 -0.001 -0.001
## emt.L:.L:AQ  0.001 -0.001  0.001  0.001 -0.001 -0.085  -0.001  0.001  0.001
##             l.L:AQ e.L:.L:I
## emotion.L                  
## live.L                     
## IRIEC                      
## AQ                         
## emtn.L:lv.L                
## emt.L:IRIEC                
## liv.L:IRIEC                
## emotin.L:AQ                
## live.L:AQ                  
## e.L:.L:IRIE  0.001         
## emt.L:.L:AQ -0.003 -0.017  
## 
## Robustness weights for the residuals: 
##  2311 weights are ~= 1. The remaining 685 ones are summarized as
##    Min. 1st Qu.  Median    Mean 3rd Qu.    Max. 
##  0.0277  0.4880  0.6980  0.6670  0.8910  0.9990 
## 
## Robustness weights for the random effects: 
##  135 weights are ~= 1. The remaining 15 ones are summarized as
##    Min. 1st Qu.  Median    Mean 3rd Qu.    Max. 
##  0.0312  0.0461  0.2590  0.2250  0.3590  0.4270 
## 
## Rho functions used for fitting:
##   Residuals:
##     eff: smoothed Huber (k = 1.345, s = 10) 
##     sig: smoothed Huber, Proposal 2 (k = 1.345, s = 10) 
##   Random Effects, variance component 1 (subject):
##     eff: smoothed Huber (k = 5.55, s = 10) 
##     vcp: smoothed Huber (k = 5.55, s = 10)
```

```
coefs_CS50_2f1 <- data.frame(coef(summary(CS50_2f1)))
coefs_CS50_r2f1 <- data.frame(coef(summary(CS50_r2f1)))
p_values_CS50_r2f1 <- 2 * pt(abs(coefs_CS50_r2f1[, "t.value"]), coefs_CS50_2f1$df, lower = FALSE)
p_values_CS50_r2f1
```

```
##  [1] 2.711214e-05 2.782212e-04 4.176061e-02 4.249193e-01 5.312933e-01
##  [6] 1.692562e-04 2.183880e-01 6.026104e-01 5.018778e-01 8.288115e-01
## [11] 5.691541e-01 7.294526e-01
```
