# Supplementary figures and images for: An investigation of the modulatory effects of empathic and autistic traits on emotional and facial motor responses during live social interactions
Source: PLoS One. 2024 Jan 9;19(1):e0290765. doi: 10.1371/journal.pone.0290765 (PMC10775989; doi:10.1371/journal.pone.0290765)

**A**

Valence

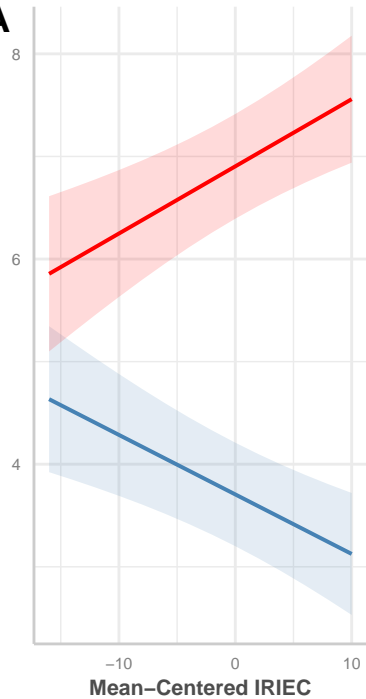**B**

Arousal

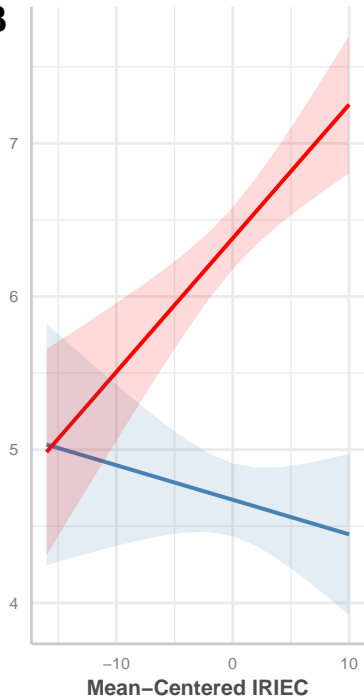**C**

Corrugator Responses

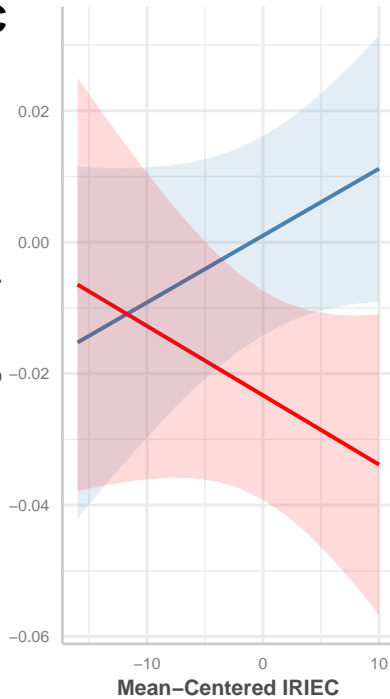**Emotion**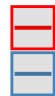

Positive

Negative

Supplement: S1 File — (ZIP) [file pone.0290765.s001.zip › Data_Code/Fig4_2f94.pdf]
