# Supplementary material for: An investigation of the modulatory effects of empathic and autistic traits on emotional and facial motor responses during live social interactions
Source: PLoS One. 2024 Jan 9;19(1):e0290765. doi: 10.1371/journal.pone.0290765 (PMC10775989; doi:10.1371/journal.pone.0290765)
Supplement: S1 Table — (DOCX) [file pone.0290765.s002.docx]

#### S1 Table. Statistical Summary of Valence Ratings with Robust Estimation

**Fixed Effects**

| **Effect** | **Beta** | **SE** | **df** | **t-value** | **Pr(>\|t\|)** |
| --- | --- | --- | --- | --- | --- |
| Intercept | 5.280 | 0.284 | 0.990 | 18.604 | 0.034* |
| Emotion | 2.256 | 0.101 | 91.000 | 22.402 | < 0.001* |
| Presentation | 0.147 | 0.037 | 91.000 | 3.933 | < 0.001* |
| E * P | 0.373 | 0.060 | 91.000 | 6.248 | < 0.001* |
| IRIEC | 0.002 | 0.008 | 90.651 | 0.231 | 0.818 |
| IRIEC * E | 0.087 | 0.020 | 91.000 | 4.378 | < 0.001* |
| IRIEC * P | 0.002 | 0.007 | 91.000 | 0.281 | 0.779 |
| IRIEC * E * P | -0.006 | 0.012 | 91.000 | -0.549 | 0.584 |
| AQ | 0.007 | 0.006 | 90.398 | 1.080 | 0.282 |
| AQ * E | 0.029 | 0.015 | 91.000 | 1.951 | 0.052 |
| AQ * P | -0.009 | 0.006 | 91.000 | -1.605 | 0.112 |
| AQ * E * P | -0.006 | 0.009 | 91.000 | -0.722 | 0.472 |

**Random Effects**

| **Group** | **Effect** | **Variance** | **SD** | **Corr. I.** | **Corr. E.** | **Corr. P.** |
| --- | --- | --- | --- | --- | --- | --- |
| Subject | Intercept | 0.134 | 0.367 |  |  |  |
|  | E | 0.869 | 0.932 | 0.23 |  |  |
|  | P | 0.083 | 0.289 | -0.07 | 0.03 |  |
|  | E * P | 0.227 | 0.486 | 0.19 | 0.06 | 0.55 |
| Type | Intercept | 0.150 | 0.387 |  |  |  |
| Residual | | 0.328 | 0.573 |  |  |  |

Formula: Valence ~ 1 + emotional_condition * presentation_condition * IRIEC + emotional_condition * presentation_condition * AQ + (1 + emotional_condition * presentation_condition | subject) + (1 | Type). Number of observations: 1,504. Number of subjects: 94. Robustness weights for the residuals of 1,259 data points are ~= 1. Abbreviations: AQ: autism spectrum quotient; CI: 95 % confidence interval; Corr. I.: correlation with the random effect of the intercept; Corr. E.: correlation with the random effect of the emotional conditions; df: Satterthwaite approximations of the degrees of freedom based on the non-robust estimation; IRIEC: empathic concern subscale of the Interpersonal Reactivity Index; SE: standard error; * *p* < 0.05
