# Supplementary material for: An investigation of the modulatory effects of empathic and autistic traits on emotional and facial motor responses during live social interactions
Source: PLoS One. 2024 Jan 9;19(1):e0290765. doi: 10.1371/journal.pone.0290765 (PMC10775989; doi:10.1371/journal.pone.0290765)
Supplement: S2 Table — (DOCX) [file pone.0290765.s003.docx]

#### S2 Table. Statistical Summary of Valence Ratings of 50 Participants with Robust Estimation

**Fixed Effects**

| **Effect** | **Beta** | **SE** | **df** | **t-value** | **Pr(>\|t\|)** |
| --- | --- | --- | --- | --- | --- |
| Intercept | 5.495 | 0.067 | 47.000 | 82.28 | < 0.001* |
| Emotion | 2.295 | 0.163 | 47.000 | 14.04 | < 0.001* |
| Presentation | 0.078 | 0.050 | 47.000 | 1.55 | 0.127 |
| E * P | 0.140 | 0.074 | 47.000 | 1.91 | 0.063 |
| IRIEC | 0.007 | 0.013 | 47.000 | 0.55 | 0.586 |
| IRIEC * E | 0.125 | 0.032 | 47.000 | 3.87 | < 0.001* |
| IRIEC * P | -0.001 | 0.010 | 47.000 | -0.09 | 0.926 |
| IRIEC * E * P | 0.007 | 0.015 | 47.000 | 0.51 | 0.614 |
| AQ | 0.012 | 0.010 | 47.398 | 1.19 | 0.241 |
| AQ * E | 0.031 | 0.024 | 47.000 | 1.25 | 0.218 |
| AQ * P | -0.017 | 0.008 | 47.000 | -2.29 | 0.027* |
| AQ * E * P | -0.017 | 0.011 | 47.000 | -1.51 | 0.139 |

**Random Effects**

| **Group** | **Effect** | **Variance** | **SD** | **Corr. I.** | **Corr. E.** | **Corr. P.** |
| --- | --- | --- | --- | --- | --- | --- |
| Subject | Intercept | 0.182 | 0.427 |  |  |  |
|  | E | 1.189 | 1.090 | 0.27 |  |  |
|  | P | 0.067 | 0.259 | -0.04 | 0.07 |  |
|  | E * P | 0.153 | 0.391 | 0.06 | -0.05 | 0.73 |
| Residual | | 0.391 | 0.626 |  |  |  |

Formula: Valence ~ 1 + emotional_condition * presentation_condition * IRIEC + emotional_condition * presentation_condition * AQ + (1 + emotional_condition * presentation_condition | subject). Number of observations: 800. Number of subjects: 50. Robustness weights for the residuals of 664 data points are ~= 1. Abbreviations: See S1 Table footnotes.
