# Supplementary material for: An investigation of the modulatory effects of empathic and autistic traits on emotional and facial motor responses during live social interactions
Source: PLoS One. 2024 Jan 9;19(1):e0290765. doi: 10.1371/journal.pone.0290765 (PMC10775989; doi:10.1371/journal.pone.0290765)
Supplement: S3 Table — (DOCX) [file pone.0290765.s004.docx]

#### S3 Table. Statistical Summary of Arousal Ratings with Robust Estimation

**Fixed Effects**

| **Effect** | **Beta** | **SE** | **df** | **t-value** | **Pr(>\|t\|)** |
| --- | --- | --- | --- | --- | --- |
| Intercept | 5.523 | 0.067 | 91.000 | 82.916 | < 0.001* |
| Emotion | 1.244 | 0.127 | 91.000 | 9.830 | < 0.001* |
| Presentation | 0.277 | 0.056 | 91.000 | 4.990 | < 0.001* |
| E * P | 0.175 | 0.052 | 91.000 | 3.334 | 0.001* |
| IRIEC | 0.025 | 0.013 | 91.000 | 1.921 | 0.058 |
| IRIEC *E | 0.083 | 0.025 | 91.000 | 3.321 | 0.001* |
| IRIEC * P | 0.003 | 0.011 | 91.000 | 0.302 | 0.763 |
| IRIEC * E * P | -0.009 | 0.010 | 91.000 | -0.857 | 0.394 |
| AQ | 0.011 | 0.010 | 91.000 | 1.123 | 0.264 |
| AQ * E | 0.013 | 0.019 | 91.000 | 0.722 | 0.472 |
| AQ * P | 0.002 | 0.008 | 91.000 | 0.277 | 0.782 |
| AQ * E * P | -0.008 | 0.008 | 91.000 | -1.030 | 0.306 |

**Random Effects**

| **Group** | **Effect** | **Variance** | **SD** | **Corr. I.** | **Corr. E.** | **Corr. P.** |
| --- | --- | --- | --- | --- | --- | --- |
| Subject | Intercept | 0.368 | 0.607 |  |  |  |
|  | E | 1.378 | 1.174 | -0.16 |  |  |
|  | P | 0.217 | 0.465 | 0.01 | -0.27 |  |
|  | E * P | 0.127 | 0.356 | 0.10 | 0.28 | 0.45 |
| Residual | | 0.483 | 0.695 |  |  |  |

Formula: Arousal ~ 1 + emotional_condition * presentation_condition * IRIEC + emotional_condition * presentation_condition * AQ + (1 + emotional_condition * presentation_condition | subject). Number of observations: 1,504. Number of subjects: 94. Robustness weights for the residuals of 1,214 data points are ~= 1. Abbreviations: See S1 Table footnotes.
