# Supplementary material for: An investigation of the modulatory effects of empathic and autistic traits on emotional and facial motor responses during live social interactions
Source: PLoS One. 2024 Jan 9;19(1):e0290765. doi: 10.1371/journal.pone.0290765 (PMC10775989; doi:10.1371/journal.pone.0290765)
Supplement: S4 Table — (DOCX) [file pone.0290765.s005.docx]

#### S4 Table. Statistical Summary of Arousal Ratings of 50 Participants with Robust Estimation

**Fixed Effects**

| **Effect** | **Beta** | **SE** | **df** | **t-value** | **Pr(>\|t\|)** |
| --- | --- | --- | --- | --- | --- |
| Intercept | 5.494 | 0.105 | 47.000 | 52.34 | < 0.001* |
| Emotion | 1.399 | 0.179 | 47.000 | 7.80 | < 0.001* |
| Presentation | 0.111 | 0.074 | 47.000 | 1.49 | 0.142 |
| E * P | 0.065 | 0.069 | 47.000 | 0.94 | 0.352 |
| IRIEC | 0.022 | 0.021 | 47.000 | 1.05 | 0.298 |
| IRIEC *E | 0.131 | 0.036 | 47.000 | 3.68 | <0.001* |
| IRIEC * P | -0.004 | 0.015 | 47.000 | -0.30 | 0.768 |
| IRIEC * E * P | -0.002 | 0.014 | 47.000 | -0.17 | 0.867 |
| AQ | 0.024 | 0.016 | 47.000 | 1.52 | 0.135 |
| AQ * E | 0.011 | 0.027 | 47.000 | 0.40 | 0.694 |
| AQ * P | -0.010 | 0.011 | 47.000 | -0.85 | 0.398 |
| AQ * E * P | -0.007 | 0.010 | 47.000 | -0.71 | 0.483 |

**Random Effects**

| **Group** | **Effect** | **Variance** | **SD** | **Corr. I.** | **Corr. E.** | **Corr. P.** |
| --- | --- | --- | --- | --- | --- | --- |
| Subject | Intercept | 0.475 | 0.689 |  |  |  |
|  | E | 1.417 | 1.190 | -0.01 |  |  |
|  | P | 0.183 | 0.428 | -0.07 | -0.07 |  |
|  | E * P | 0.074 | 0.273 | 0.47 | 0.47 | 0.67 |
| Residual | | 0.576 | 0.759 |  |  |  |

Formula: Arousal ~ 1 + emotional_condition * presentation_condition * IRIEC + emotional_condition * presentation_condition * AQ + (1 + emotional_condition * presentation_condition | subject). Number of observations: 800. Number of subjects: 50. Robustness weights for the residuals of 641 data points are ~= 1. Abbreviations: See S1 Table footnotes.
