# Supplementary material for: An investigation of the modulatory effects of empathic and autistic traits on emotional and facial motor responses during live social interactions
Source: PLoS One. 2024 Jan 9;19(1):e0290765. doi: 10.1371/journal.pone.0290765 (PMC10775989; doi:10.1371/journal.pone.0290765)
Supplement: S5 Table — (DOCX) [file pone.0290765.s006.docx]

#### S5 Table. Statistical Summary of Zygomaticus Responses with Robust Estimation

**Fixed Effects**

| **Effect** | **Beta** | **SE** | **df** | **t-value** | **Pr(>\|t\|)** |
| --- | --- | --- | --- | --- | --- |
| Intercept | 8.840e-03 | 3.529e-03 | 4.047 | 2.505 | 0.066 |
| Emotion | 1.493e-02 | 3.483e-03 | 85.49 | 4.287 | <0.001* |
| Presentation | -3.453e-04 | 2.119e-03 | 74.10 | -0.163 | 0.871 |
| E * P | 1.101e-02 | 2.774e-03 | 10540 | 3.970 | <0.001* |
| IRIEC | 3.704e-04 | 6.982e-04 | 85.01 | 0.531 | 0.597 |
| IRIEC * E | -1.620e-04 | 6.924e-04 | 85.59 | -0.234 | 0.816 |
| IRIEC * P | -2.537e-04 | 4.295e-04 | 80.81 | -0.591 | 0.556 |
| IRIEC * E * P | -1.165e-04 | 5.650e-04 | 10540 | -0.206 | 0.837 |
| AQ | 6.285e-04 | 5.218e-04 | 84.79 | 1.204 | 0.232 |
| AQ * E | -3.087e-04 | 5.157e-04 | 85.28 | -0.599 | 0.551 |
| AQ * P | 2.801e-05 | 3.161e-04 | 76.41 | 0.089 | 0.930 |
| AQ * E * P | 2.730e-04 | 4.146e-04 | 10540 | 0.659 | 0.510 |

**Random Effects**

| **Group** | **Effect** | **Variance** | **SD** | **Corr. I.** | **Corr. E.** |
| --- | --- | --- | --- | --- | --- |
| Subject | Intercept | 0.0009 | 0.030 |  |  |
|  | Emotion | 0.0007 | 0.026 | 0.03 |  |
|  | Presentation | 0.00005 | 0.007 | -0.52 | 0.84 |
| Residual | | 0.020 | 0.140 |  |  |

Formula: ZM ~ 1 + emotional_condition * presentation_condition * IRIEC + emotional_condition * presentation_condition * AQ + (1 + emotional_condition + presentation_condition | subject) + (1 | Type). Number of observations: 10,787. Number of subjects: 93. Robustness weights for the residuals of 8,229 data points are ~= 1. Abbreviations: See S1 Table footnotes.
