# Supplementary material for: An investigation of the modulatory effects of empathic and autistic traits on emotional and facial motor responses during live social interactions
Source: PLoS One. 2024 Jan 9;19(1):e0290765. doi: 10.1371/journal.pone.0290765 (PMC10775989; doi:10.1371/journal.pone.0290765)
Supplement: S6 Table — (DOCX) [file pone.0290765.s007.docx]

#### S6 Table. Statistical Summary of Zygomaticus Responses of 50 Participants with Robust Estimation

**Fixed Effects**

| **Effect** | **Beta** | **SE** | **df** | **t-value** | **Pr(>\|t\|)** |
| --- | --- | --- | --- | --- | --- |
| Intercept | 6.870e-03 | 4.660e-03 | 46.99 | 1.474 | 0.147 |
| Emotion | 2.127e-02 | 6.459e-03 | 46.99 | 3.294 | 0.002* |
| Presentation | 2.204e-04 | 2.612e-03 | 95.55 | 0.844 | 0.401 |
| E * P | 1.041e-02 | 3.479e-03 | 2890 | 2.991 | 0.003* |
| IRIEC | 1.519e-04 | 9.239e-04 | 46.99 | 1.644 | 0.107 |
| IRIEC * E | 1.078e-03 | 1.281e-03 | 46.99 | 0.842 | 0.404 |
| IRIEC * P | -6.469e-05 | 5.178e-04 | 95.51 | -0.125 | 0.901 |
| IRIEC * E * P | 6.164e-04 | 6.898e-04 | 2890 | 0.894 | 0.372 |
| AQ | 5.594e-04 | 7.102e-04 | 46.99 | 0.788 | 0.435 |
| AQ * E | -1.025e-04 | 9.844e-04 | 47.00 | -0.104 | 0.917 |
| AQ * P | -5.349e-04 | 3.984e-04 | 95.86 | -1.343 | 0.183 |
| AQ * E * P | -7.667e-04 | 5.308e-04 | 2890 | -1.444 | 0.149 |

**Random Effects**

| **Group** | **Effect** | **Variance** | **SD** | **Corr. I.** | **Corr. E.** |
| --- | --- | --- | --- | --- | --- |
| Subject | Intercept | 8.743e-04 | 0.030 |  |  |
|  | Emotion | 1.669e-03 | 0.041 | <0.01 |  |
|  | Presentation | 3.593e-05 | 0.006 | -0.05 | 1.00 |
| Residual | | 8.365e-03 | 0.091 |  |  |

Formula: ZM ~ 1 + emotional_condition * presentation_condition * IRIEC + emotional_condition * presentation_condition * AQ + (1 + emotional_condition + presentation_condition | subject). Number of observations: 2,996. Number of subjects: 50. Robustness weights for the residuals of 2,283 data points are ~= 1. Abbreviations: See S1 Table footnotes.
