# Supplementary material for: An investigation of the modulatory effects of empathic and autistic traits on emotional and facial motor responses during live social interactions
Source: PLoS One. 2024 Jan 9;19(1):e0290765. doi: 10.1371/journal.pone.0290765 (PMC10775989; doi:10.1371/journal.pone.0290765)
Supplement: S7 Table — (DOCX) [file pone.0290765.s008.docx]

#### S7 Table. Statistical Summary of Corrugator Responses with Robust Estimation

**Fixed Effects**

| **Effect** | **Beta** | **SE** | **df** | **t-value** | **Pr(>\|t\|)** |
| --- | --- | --- | --- | --- | --- |
| Intercept | -0.0109 | 0.0027 | 1.346 | -1.791 | 0.272 |
| Emotion | -0.0154 | 0.0031 | 89.66 | -4.938 | <0.001* |
| Presentation | -0.0025 | 0.0020 | 63.73 | -0.920 | 0.361 |
| E * P | -0.0114 | 0.0025 | 10500 | -4.530 | <0.001* |
| IRIEC | -0.0001 | 0.0005 | 82.67 | -0.073 | 0.942 |
| IRIEC * E | -0.0016 | 0.0006 | 90.46 | -2.548 | 0.013* |
| IRIEC * P | -0.0003 | 0.0004 | 67.29 | -0.768 | 0.445 |
| IRIEC * E * P | 0.0006 | 0.0005 | 10500 | 1.122 | 0.262 |
| AQ | 0.0004 | 0.0004 | 79.36 | 0.982 | 0.329 |
| AQ * E | -0.0003 | 0.0005 | 89.73 | -0.623 | 0.535 |
| AQ * P | -0.0001 | 0.0003 | 64.75 | -0.647 | 0.520 |
| AQ * E * P | 0.0006 | 0.0004 | 10500 | 1.475 | 0.140 |

**Random Effects**

| **Group** | **Effect** | **Variance** | **SD** | **Corr. I.** | **Corr. P.** |
| --- | --- | --- | --- | --- | --- |
| Subject | Intercept | 4.201e-04 | 0.020 |  |  |
|  | Emotion | 5.191e-04 | 0.023 | 0.02 |  |
|  | Presentation | 5.943e-05 | 0.008 | 0.97 | -0.21 |
| Type | Intercept | 6.236e-05 | 0.008 |  |  |
| Residual | | 1.591e-02 | 0.126 |  |  |

Formula: CS ~ 1 + emotional_condition * presentation_condition * IRIEC + emotional_condition * presentation_condition * AQ + (1 + emotional_condition + presentation_condition | subject) + (1 | Type). Number of observations: 10,787. Number of subjects: 93. Robustness weights for the residuals of 8,344 data points are ~= 1. Abbreviations: See S1 Table footnotes.
