# Supplementary material for: An investigation of the modulatory effects of empathic and autistic traits on emotional and facial motor responses during live social interactions
Source: PLoS One. 2024 Jan 9;19(1):e0290765. doi: 10.1371/journal.pone.0290765 (PMC10775989; doi:10.1371/journal.pone.0290765)
Supplement: S8 Table — (DOCX) [file pone.0290765.s009.docx]

#### S8 Table. Statistical Summary of Corrugator Responses of 50 Participants with Robust Estimation

**Fixed Effects**

| **Effect** | **Beta** | **SE** | **df** | **t-value** | **Pr(>\|t\|)** |
| --- | --- | --- | --- | --- | --- |
| Intercept | -0.0197 | 0.0042 | 47.55 | -4.645 | <0.001* |
| Emotion | -0.0229 | 0.0058 | 47.04 | -3.929 | <0.001* |
| Presentation | -0.0069 | 0.0033 | 57.87 | -2.082 | 0.042* |
| E * P | -0.0142 | 0.0038 | 2890 | -3.766 | <0.001* |
| IRIEC | -0.0007 | 0.0008 | 47.54 | -0.805 | 0.425 |
| IRIEC * E | -0.0014 | 0.0012 | 47.03 | -1.248 | 0.218 |
| IRIEC * P | -0.0003 | 0.0007 | 57.85 | -0.524 | 0.603 |
| IRIEC * E * P | -0.0004 | 0.0008 | 2890 | -0.569 | 0.569 |
| AQ | 0.0004 | 0.0006 | 47.61 | 0.631 | 0.531 |
| AQ * E | -0.0006 | 0.0009 | 47.09 | -0.677 | 0.502 |
| AQ * P | -0.0001 | 0.0005 | 58.00 | -0.217 | 0.829 |
| AQ * E * P | -0.0002 | 0.0006 | 2890 | -0.346 | 0.729 |

**Random Effects**

| **Group** | **Effect** | **Variance** | **SD** | **Corr. I.** | **Corr. P.** |
| --- | --- | --- | --- | --- | --- |
| Subject | Intercept | 0.0007 | 0.026 |  |  |
|  | Emotion | 0.0013 | 0.035 | 0.00 |  |
|  | Presentation | 0.0002 | 0.013 | 1.00 | -0.01 |
| Residual | | 0.0099 | 0.099 |  |  |

Formula: CS ~ 1 + emotional_condition * presentation_condition * IRIEC + emotional_condition * presentation_condition * AQ + (1 + emotional_condition + presentation_condition | subject). Number of observations: 2,996. Number of subjects: 50. Robustness weights for the residuals of 2,311 data points are ~= 1. Abbreviations: See S1 Table footnotes.
