# Supplementary material for: An investigation of the modulatory effects of empathic and autistic traits on emotional and facial motor responses during live social interactions
Source: PLoS One. 2024 Jan 9;19(1):e0290765. doi: 10.1371/journal.pone.0290765 (PMC10775989; doi:10.1371/journal.pone.0290765)
Supplement: Attachment — Submitted filename: Reply_to_Reviewers_Rev1_final.pdf [file pone.0290765.s010.pdf]

Reviewer #1:

Thank you for this opportunity to review this manuscript, which describes a study that examined facial mimicry and subjective ratings of emotional valence and arousal towards pre-recorded and live facial expressions of happiness and anger. Moreover, individual differences in empathic and autistic traits were taken into account. Overall, the authors found that responses to emotional expressions were enhanced in live conditions, compared to pre-recorded conditions, especially for individuals with higher affective empathy. Whilst individuals with higher autistic traits showed stronger zygomaticus responses to pre-recorded conditions than to live conditions. This study used a paradigm that allows for testing in live situations, and provided valuable data about how the (spontaneous) responses towards different types of emotional stimuli may be modulated by different personal traits. However, I do have some concerns about the manuscript in its current form, which dampen my enthusiasm. I will elaborate in greater detail below.

Main concerns:

1. I am afraid the current Introduction and Discussion do not reflect a correct picture of autism. Autistic people were described as having deficits that lead to low empathy and low sensitivity to social-emotional stimuli. However, this is not true. Increasing studies have shown that autistic people have the motivation to make social contacts and need a sense of belonging; and that autistic individuals are as emotionally aroused – and sometimes over-aroused – as non-autistic individuals. It also has to be taken into account that very often empathy of autistic people is evaluated by non-autistic people in the literature. Autistic individuals may be different in how they express and react to emotions, but they do feel it. The authors are suggested to modify the language in this respect (e.g., deficits, lacking) and to review the literature also from a non-medical point of view, to make this study more connected to the lived experiences of autistic individuals, or individuals with high autistic traits.

Authors' response: We are grateful to the reviewer for highlighting this issue. We have updated the Introduction (lines 59–81) thoroughly (particularly the paragraphs that discuss autistic traits and ASD; lines 499–557) for a more neutral discussion of early findings, and have included more contemporary theories concerning social cognition in individuals with ASD or highly autistic traits.

2. The analyses were conducted with rather complex LME models. However, the model complexity was only assessed in terms of random effects, but on of fixed effects. I wonder if the authors have a reason for this decision, and if the authors did an a priori power analysis to know whether the

observations are enough for running such complex models. Moreover, the models seem not well grounded by the research questions and hypotheses. The inclusion of valence and IRI-perspective taking came as a surprise and the authors did not have hypotheses for these analyses. Due to these doubts, I am uncertain to what extent the results can be interpreted in the current form.

Authors' response: We will address the reviewer's concerns via several bullet points.

- The existing literature on the complexity of LME has focused on random effects in models; for n variance components, there will be a maximum of n(n+1)/2 model parameters, while fixed-effect parameters usually do not drastically increase the degree of freedom. There is also a debate as to whether the parsimonious [1] or keep-it-maximal [2] approach is best. We adopted the parsimonious approach for random effects, and the keep-it-maximal approach for fixed effects.

- We considered both valence and arousal ratings (as dependent variables) to be measures of emotional contagion. Note that excluding valence will reduce the number of models, but not model complexity.

- We originally included IRI perspective-taking (IRIPT) in light of Chartrand et al.'s report on the utility of IRIPT, but not IRIEC, for predicting the chameleon effect (spontaneous motor mimicry) [3]. They conducted a between-group comparison based on a median split of IRIPT and IRIEC. As pointed out by Reviewer 1 (comment 14), this appears inconsistent with later studies, which found that individual differences in emotional empathy were more associated with spontaneous facial mimicry. It is also the case that IRIPT yielded no significant results in our study. Therefore, we excluded IRIPT and its interaction terms from the models and updated the results. We have also added a note in the Method section, entitled "Statistical analysis of live interaction data," to the effect that "Note that we initially included the perspective taking subscale of IRI and its interaction terms, but dropped these from the final models because neither the main nor interaction effects involving this subscale were significant" (lines 255–257). Note that after simplifying the models, IRIEC and AQ appeared to have three-way interaction effects on valence ratings.

- We did not perform a priori power analysis, due to a lack of previous reports of effect sizes for interaction effects in the context of the "live effect". However, we have now included power estimates for significant results as a reference for future studies. The data table in the supplementary material may also be used for sample size estimation by future studies. As expected, a different sample size was required to achieve a power of 0.8 for each effect, and at

the time of data acquisition, 50 was the realistic limit of the sample size that we could achieve.

- Regarding the complexity of the statistical models, no standard method of determining whether an LME model includes too many parameters exists. We have relied on "warnings" of singular convergence or convergence failure, i.e., that the number of parameters to be estimated was too large for the dataset or reflected overfitting. In our iterative model comparison process, we did not select models leading to singular convergence or convergence failure (https://rpubs.com/palday/lme4-singular-convergence).

3. The manuscript would benefit from a more in-depth discussion on the findings. Currently, the authors mainly described how the findings are in line with previous studies, and did not really give a holistic picture of the implications and contributions of this study. Also, the autistic traits were only found to have an effect on the ZM reactions, but not on subjectively ratings of emotional valence and arousal. This finding shows that the participants with high autistic traits in this study felt similarly emotional and aroused as those with low autistic traits, echoing the point made earlier in comment 1. This is a result not expected by the authors, and thus worth further discussions.

Authors' response: We are grateful to the reviewer for highlighting this issue. In the revised manuscript, we have updated and extended the discussion, and highlight the implications of our results in the context of contemporary understanding of ASD and autistic traits. Indeed, as the reviewer has pointed out, our results were more compatible with recent findings that individuals with ASD or high AQs do not differ from those with low AQs with respect to emotional contagion and spontaneous facial mimicry. Our findings regarding the effect of AQ appeared to be more specific to the live effect. We now discuss this point in the discussion (lines 499–541).We have also added a paragraph addressing the potential drawback of exclusively examining the participants' autistic traits, and not the dyadic differences (lines 542–557).

Other concerns:

4. Page 9, 2nd paragraph, last line: Better add 'autonomic' before 'facial mimicry' given that voluntary facial mimicry has been reported to be present in autistic individuals.

Authors' response: We have followed the reviewer's suggestion.

5. Page 10, line 3 "than suggested by previous data": This could be confusing. On which basis

were the previous studies compared?

Authors' response: We agree with the reviewer that this expression was vague and have changed it to "than using prerecorded photos or videos."

6. Page 10, 2nd paragraph "we expected that individuals with higher emotional empathy ... would exhibit higher levels of emotional contagion and greater spontaneous facial mimicry of live stimuli": From this hypothesis, it is unclear what 'emotion contagion' is referred to. Is it referring to the subjective ratings, or denoting the latent layer of facial mimicry?

Authors' response: We used subjective ratings as measures of emotional contagion. This information has been added to the paragraph in question, which now reads "Based on previous findings, we expected dynamic live expressions to enhance the modulatory effects of empathic and autistic traits. Specifically, we expected the live conditions to enhance the correlations between emotional empathy, as measured using the empathic concern subscale of the IRI, and emotional contagion, measured by the subjective valence and arousal ratings, as well as the positive correlation between emotional empathy and the level of spontaneous facial mimicry" (line 99-104). In this manuscript, we treat emotional contagion and facial mimicry separately, since it remains unclear whether there is a causal relationship between facial mimicry and emotional contagion, despite their frequent co-occurrence [4].

7. Figure 2: This figure presents only video-recorded stimuli; this should better be specify. If possible, adding examples of live stimuli could be helpful.

Authors' response: We have now included examples of both prerecorded and live stimuli in Figure 2.

8. Figure 3: It appears that participants were presented 'Video' or 'Real Person' before each stimulus was shown to them. I believe this design requires better explanations: Wouldn't such instructions already prime the participants' responses? I wonder whether the authors expect this design to have an effect on the results.

Authors' response: The live effect that this study aimed to investigate is the effect of the knowledge that live images are being transmitted (audience effect, watching eye effect, or "we mode"). In an earlier publication [5], the validating rating study showed prerecorded and live performance clips to naïve participants without such instruction, and the ratings showed no "live effect". This effect was anticipated.

9. Page 12 – autism spectrum quotient: the last sentence is already in Statistical Analyses and not needed in this section.

Authors' response: We have followed the reviewer's suggestion and removed the sentence.

10. Page 13 – paradigm and procedures: Throughout the Method section, there are many different versions of trial numbers. After reading multiple times, I understood that sometimes only part of the task was referred to. Yet it still remains unclear to me how many trials were presented for participants to rate valence and arousal, and whether these ratings were given right after each passive viewing trial, or in different blocks. It would be better if the authors could describe the procedures altogether in one section, rather than separating them in different parts as they are now.

Authors' response: The procedure was conducted sequentially, as follows: informed consent → 3-minute conversation → 8 practice passive viewing trials → 60 passive viewing trials → 8 practice rating trials → 16 rating trials. We recorded EMG data only in the passive viewing trials, because participants made movements (keyboard presses) during rating trials. In rating trials, participants first viewed the dynamic facial expression, exactly as in the passive viewing trials, and then gave valence and arousal ratings (Fig. 1). We have added sentences to the first paragraph (lines 187–190) of the "paradigm and procedures" to clarify the overall procedure sequence.

11. Page 14 – statistical analyses "… and the difference in EMG (ZM and CS) between the neutral (0-1 s after stimulus onset) and maximal phases (2.5-3.5 s after stimulus onset) of the dynamic facial expression": I understand that the baseline had already be deducted, and it comprised of the 3 seconds before the onset and 1 second after the onset, which is different from the description shown here.

Authors' response: The EMG data preprocessing included a "baseline correction" step prior to rectification and log transformation. The baseline for this step was the mean of the EMG values from 3 seconds before to 1 second after stimulus onset (i.e., 2 seconds before and after the onset of the trial). All data points in the trial/epoch (as EEGLAB terms it) were subtracted from this value to correct for low-frequency drifts in continuous recordings, and were then rectified and log-transformed. The dependent variable in the statistical analysis was the difference between the neutral and maximal phases of dynamic facial expression after rectification and log transformation. No conflict emerged between the description of the EMG preprocessing and statistical analyses.

12. Results – questionnaire scores: The total IRI score was reported, but not the IRI-Empathy Concern score, which should be the score reported here as it is part of the research question.

Authors' response: We have modified this paragraph and reported the descriptive statistics of the IRIEC scores, as suggested.

13. Page 23, line 4 "As expected, participants with higher trait emotional empathy (IRIEC) showed larger differences in subjective arousal between positive and negative facial expressions in the live compared to video conditions": However, no hypothesis was made in regard to the difference in valence.

Authors' response: We expected the level of emotional contagion in the live conditions to be positively correlated with the IRIEC. We used subjective ratings of both valence and arousal [6], to measure the level of emotional contagion. In the Results section of the first submission, an interaction effect of trait measures on arousal ratings, but not on valence ratings, was reported; this discrepancy could have been discussed in greater depth. However, in this revised version, we have removed the parameters for IRIPT and their interaction effects from the models, based on comment 3 of Reviewer 1, and a significant three-way interaction effect of IRIEC on valence ratings emerged, similar to that seen on arousal ratings. The results appear to be more coherent in terms of emotion contagion.

14. Page 23, 2nd paragraph: the sudden switch to cognitive empathy seems irrelevant to the topic discussed in this paragraph.

Authors' response: The literature review concerning the relationship between empathy and spontaneous motor mimicry (not limited to facial expressions) revealed Chartrand et al.'s report of the relationship between IRIPT (but not IRIEC) and the chameleon effect, as early evidence [3]. However, although it did not seem relevant to the literature reported in the first version of the manuscript, most other recent studies reported that emotional empathy was more influential with respect to spontaneous facial mimicry, including the reports cited in the current version of the manuscript. Considering Reviewer 1's concern about the model's complexity (comment 2) and non-significance of IRIPT in our data, we have decided to remove IRIPT from the statistical model, and have also removed sentences relating to Chartrand et al.'s study.

15. Page 24, lines 4-5 "watching eye effect and audience effect": A brief explanation on these effects may help more readers understand the information presented here.

Authors' response: We have now included short descriptions of "we mode," the "audience effect," and the "watching eye effect" in the Discussion (lines 492–498).

16. It should be noted and addressed that many effects found in this study were with rather small effect sizes, such as the interaction effect of AQ x Presentation Mode on ZM reactions. This again makes me concerned about the complexity of the models and the interpretation of the results.

Authors' response: We have added this point to the Limitations section (lines 589-591)

Reviewer #2

In the current study, the authors aim to examine whether the "liveliness" of social encounters, i.e. live interactions vs. video presentations, have an effect on the modulation of the subjective emotional experience as well as facial mimicry by individual differences in empathic traits and autistic traits in a healthy female sample. To achieve this, participants were seated in front of a self-designed and previously validated live image relay system and were either presented with live emotional facial expressions (positive vs. negative) of a model seated on the other side, or with video-taped expressions of the same model. Replicating previous results, arousal ratings as well as facial mimicry (indicated by facial muscle (de-)activations) were higher/more pronounced in live interactions compared to video observations. Additionally, higher scores on the Empathic Concern subscale of the IRI were associated a stronger difference in corrugator supercilii between positive and negative expressions responses across presentation conditions, and with a stronger difference in arousal ratings as well as zygomaticus major responses between positive and negative expressions in the live vs. the video condition, which the authors interpreted as a higher emotional engagement with the other, especially in live interactions. Higher autistic traits, however, were associated with higher zygomaticus responses in the video compared to the live condition which was suggested to originate from the decreased sensitivity to social stimuli which is commonly associated with autism. The core strength of this study is its innovative setup which allows for a comparison between live and video-taped 'interactions' while all other experimental parameters are kept constant. It is a great example of how an incremental shift to a 'second-person neuroscience' can be made possible. Further, by measuring both subjective experiences and physiological responses to emotional expressions, different levels of analysis can be described and compared to achieve a more holistic view on modulations in emotion processing associated with different trait dimensions.

Since no page and line numbers were provided, I started numbering the pages, with the title page

being page 1. All remarks can further be mapped by an indication of the paragraph in the respective section.

Major concerns:

1. Main general remarks: The authors claim that they are providing a more ecologically valid and naturalistic approach to study real-life interactions with their setup. The displayed expressions in the interaction were, however, highly posed and controlled rather than naturally occurring. The question therefore arises whether participants would have perceived a difference between live and videotaped expressions if no instructions were given, and, if not, what the additional value of setting up this 'real' live interaction was. While the setup can definitely be useful for research on social interactions, the authors should clarify why they decided for such a controlled design and in how far this decision limits the ecological validity compared to a real, uncontrolled social interaction with bidirectional information exchange. This limitation is briefly mentioned in the discussion but should be elaborated and also kept in mind when making claims about examining "live social interactions" in the current study.

<u>Authors' response</u>: In the 2020 paper, which used some of the data included in the current manuscript, the post-hoc valence and arousal ratings of naïve participants demonstrated that, without instructions, participants perceived no differences between live and videotaped expressions [5]. As Reviewer 2 described above, this allowed for more confident attribution of observed effects to the live effect (we-mode, audience effect, or watching-eye effect). As it was difficult for us to prepare videotaped and live facial expressions that were simultaneously natural and comparable, we decided to opt for the classical paradigm of spontaneous facial mimicry, and asked the models to make reproducible facial expressions, but worked on the facilities to enable the "live" conditions. We agree that real social interactions rarely occurred, such that the present study represented an incremental move toward live interactions. We have further elaborated on this issue in the Limitations section in the Discussion (lines 578–589), and hope to perform future studies with realistic bidirectional interactions using our setup.

2. Further, in the abstract, the introduction, the results and the discussion, the authors phrase their hypotheses/results in a way that a group comparison between individuals with low trait level and high trait levels (both traits) would be expected (e.g. abstract: "female individuals with low AQ scores showed [...], with the opposite pattern seen in high AQ female individuals"). As the authors treat the trait dimensions as continuous variables in their analyses, the phrasing should be

adjusted, e.g. "higher autistic trait levels were associated with …".

Authors' response: We agree with the reviewer that the description should match the analysis performed. Regarding the existing studies described in the Introduction and Discussion, most of them performed between-group comparisons (ASD vs. neurotypical or median/mean split of trait levels), so we retained the description of between-group comparisons. In describing our current results, we deemed it more appropriate to refer to the trait as a continuous variable. We have thoroughly checked and corrected the phrasing as necessary.

3. Introduction: It would be beneficial to clarify the definition of constructs which the authors are using and to be consistent in their usage. For example, that "emotional empathy" would be "reflected in EMG activities" and correlate "with self-reported feelings" in the second paragraph (p.3) seems to suggest that the emotional experience would not be part of emotional empathy. Similarly, a clarification of the operationalization of emotional contagion in the last paragraph of the discussion ("individuals with higher emotional empathy […] would exhibit higher levels of emotional contagion", p.4) would benefit to understand the author's hypotheses. A more precise usage of terms should also be considered in the discussion.

Authors' response: We are grateful to the reviewer for highlighting this issue. The relationship between spontaneous facial mimicry, emotional empathy and emotion contagion is complicated and not without controversy. We have further elaborated our position about this issue in the second paragraph of the Introduction (lines 41–50). We have also added background information about empathy and emotional contagion to the third paragraph. Spontaneous mimicry, in the context of embodied cognition, is proposed as a mechanism underlying emotional empathy. Both spontaneous mimicry and empathy have been proposed as mechanisms of emotional contagion (i.e., the transfer of affective states between people), but whether the relationship is causal remains the subject of debate [4,7,8]. Emotional contagion has also been proposed as a component of empathy. The emotional contagion process itself is also highly complicated, involving multilevel and parallel redundant mechanisms [9,10]. After consideration, we think that it might be clearer to treat these three concepts as co-occurring yet independent processes, despite the possibility that one may be in a causal relationship with another. Specifically, we examined how individual differences in emotional empathy correlated with different patterns of spontaneous mimicry and emotional contagion (as reflected in valence and arousal ratings).

4. Materials and methods: In the caption of Figure 2, it is described that the models were instructed to make "happy" and "angry" expressions whereas, in all other parts of the manuscript, the expressions are only referred to as positive/"smiling" or negative/"frowning". Given the

ambiguous examples in Figure 2 and the fact that "frowning"/activation of the corrugator supercilii is also associated with sad facial expressions, it would be a valuable information to know whether the expressions were indeed interpreted as "happy" and "angry" (and not only rated according to their valence and arousal as described in the Live performance validation section). Given that anger and sadness can have different effects with regard to mimicry and emotional empathy, the interpretation of the expressions seems to be crucial for this study.

Authors' response: We performed a post-hoc rating study of negative video clips (two per presentation condition—prerecorded or live—for each model) representing sadness and anger on a Likert scale of 1–5, with 28 naïve female participants (mean ± SD age = 27.64 ± 3.07 years; range: 20–30 years). Both negative stimuli, i.e., anger (mean ± SD = 3.36 ± 0.61) and sadness (mean ± SD = 3.01 ± 0.85), were rated highly but the paired t-test of participant-wise mean rating values indicated that participants generally experienced more anger than sadness as a result of the negative stimuli ($t = 2.55$, $df = 27$, $p = 0.017$). It is also true that participants detected a certain amount of sadness in our negative stimuli. Our EMG results showed no modulatory effect of the live effect on individual differences in the CS responses, suggesting that the level of perceived anger may have evoked more social top-down control over facial mimicry. The post-hoc rating results correspond well with findings that East Asian facial expressions are less stereotypical and exhibit greater overlap between categories [11]. We instructed the models to make their own angry expressions, and did not ask them to pose/reproduce Ekman's anger expression [12]. We made this decision based on evidence presented in Ekman's study in the 1970s demonstrating that Western angry faces were only recognized by around 67% of Japanese participants [13], and based on more recent investigations from our lab [14].

Table 1
Single-Emotion Judgment Task: Percentage of Subjects Within Each Culture Who Chose the Predicted Emotion

Nation	Happiness	Surprise	Sadness	Fear	Disgust	Anger
Estonia	90	94	86	91	71	67
Germany	93	87	83	86	61	71
Greece	93	91	80	74	77	77
Hong Kong	92	91	91	84	65	73
Italy	97	92	81	82	89	72
Japan	90	94	87	65	60	67
Scotland	98	88	86	86	79	84
Sumatra	69	78	91	70	70	70
Turkey	87	90	76	76	74	79
United States	95	92	92	84	86	81

We have added the post-hoc rating results to the Methods section under the heading "Stimuli validations" (lines 224–233), as well as an additional paragraph to the Discussion (lines 558–577).

5. Results: It was quite striking to me that, when comparing estimated means for interactions including emotion and presentation (and potentially also a trait dimension), only comparisons within [emotion] conditions but never between presentation conditions (e.g. positive-live vs positive-video) were reported, even though these comparisons seemed to be of main interest to the study. By only comparing slope differences between emotions within presentation conditions, it

remains unclear whether the effect might be driven by one emotional expression. This seems especially relevant for the EMG data if meaningful inferences about "mimicry" are aimed to be made as the authors actually do in the second paragraph of the discussion (IRIEC and facial mimicry of ZM). In the Zygomaticus major analysis, the statistics for the comparison between the AQ slopes in the video vs. the live condition are not reported. Here, the interpretation that "Individuals with high AQ scores had stronger ZM reactions to video compared to live stimuli, while individuals with low AQ scores had stronger ZM reactions to live compared to video stimuli" seems inadmissible since the slope in the video condition only seems to be steeper in the video conditions (stronger effect of autistic traits) but no group comparison has been made. The results should therefore also be discussed more carefully in the discussion.

Authors' response: We are grateful to the reviewers for highlighting both of these issues. For the interaction effects involving both emotion and presentation conditions, we have added the simple effects of emotion conditions by presentation condition (positive-live vs. negative-live, positive video vs. negative-video) to the Results section. We have also modified the description of this AQ x presentation condition interaction effect; it is now described merely in terms of there being a more positive correlation between AQ and ZM responses in the video than live conditions.

6. Discussion: As there was no emotion-specific modulatory effect of autistic traits on ZM activity, it should be clarified that the current studies results are actually not directly supporting the cited literature on facial mimicry (not "compatible"). General differences in zygomaticus activity depending on the context should rather be the focus of this discussion. Further, as the slope of the video condition seems to be, at least numerically, more affected by autistic traits than the slope of the live condition, a pure interpretation in terms of less zygomaticus activity with higher AQ traits in a live context appears to be foreshortening.

Authors' response: We agree with the reviewers. Considering the above comment together with comment 1 of Reviewer 1, we have modified the background information and discussion pertaining to autistic traits and ASD. We have also clearly stated in the Discussion that our results revealed no reduction effect of AQ on facial mimicry, and have related the AQ x presentation condition interaction effect on ZM responses to a reduction in social smiling in live conditions.

Minor comments:

7. Introduction: The authors provide a good amount of literature supporting the role of facial mimicry in emotion processing and social behaviour in the first paragraph of the introduction (p.3).

As the role of facial mimicry in emotion recognition is however still under debate (see recent meta-analysis by Holland and colleagues), the introduction would benefit from a more critical/less one-sided description. Further, as the authors only investigate the role of autistic traits and do not compare a clinical population to healthy controls, some information on the relationship between autistic trait levels and facial mimicry/empathy should be included.

Authors' response:

- We agree that the evidence supports a correlational relationship among spontaneous facial mimicry, emotional cognition, and contagion, and that causality remains unclear. We have included this information in the Introduction (lines 41–50).

- We have also included a paragraph providing background information on the relationship between autistic traits and spontaneous facial mimicry in the Introduction (lines 73–81). To the best of our knowledge, the amount of literature pertaining to the effects of autistic traits in neurotypical participants is relatively less than that comparing ASD between neurotypical groups. We have also discussed possible reasons why, contrary to previous findings, we did not find a modulatory effect of AQ on spontaneous facial mimicry.

8. Materials and methods: The motivation for some influential experimental design choices is not clear, namely:

- Pre-recorded videos/paradigm and procedure: Why did the participants have a 3min conversation before the task? Why was it necessary to include practice trials for the passive viewing task at all and a comparably large number of practice trials for the rating task? Why was there a break after 32 trials (and not 30) in the passive viewing task and why did participants only rate such a number of trials (16) in the rating task compared to the passive viewing task (60)?

Authors' response:

- The 3-minute conversation demonstrated to the participants that the prompter system actually transmitted live images of the model, as described (i.e., confirmed that we were not deceiving them). We have added this information under the "Paradigm and procedures" part of the Methods section (lines 187–188).

- The practice trials aimed to give participants an idea of what would happen, and to allow them to

ask questions before we officially commenced data collection. In passive viewing practice trials, we sometimes had to reaffirm to participants that they were required simply to focus on the screen without performing any other task. In the rating practice trials, participants sometimes required further clarification regarding the meaning of valence and arousal, or how the Likert scale should be used. We agree that the passive viewing task was simpler than the rating task, and the number of practice trials was determined arbitrarily as we designed the experiment.

- The passive viewing task was interrupted after 32 trials to ensure a balanced number of trials (n = 8) per condition; we have included this information in the Method section (line 200). The pseudo-random presentation of conditions took this into consideration.

- The relative proportions of EMG trials (passive viewing) and rating trials confirmed our prior assumption about the effect size and signal-to-noise ratio of spontaneous facial mimicry and ratings. The EMG would have a smaller effect size and lower signal-to-noise ratio than ratings, thus requiring significantly more repetitions per participant. However, participants might experience a loss of attention or interest if the passive viewing period persists for too long. Ultimately, 60 EMG trials (15 repetitions) and 16 ratings trials (4 repetitions) allowed us to restrict the session length to within 1 hour, which was the amount of time we remunerated the participants for.

9. - EMG pre-processing: Why is a baseline of 4s selected, incl. very different visual inputs, i.e., a fixation cross, the instructions and part of the stimulus?

- Statistical analyses of live interaction data: Why was a difference between the neutral and maximal phase in the EMG signals calculated if a baseline correction already took place which also, among others, included the neutral phase?

Authors' response: The "baseline correction" at the preprocessing stage aimed to address the issue of low-frequency drift over a long recording period. For the entire epoch/trial, we believe it should be around the level of the trial's beginning (i.e., the onset of the instructions), so we subtracted the average value 2 seconds before and after that from all data points. The rectification and log transformation steps took place after the baseline correction, before we extracted the dependent variable. Different dependent variables were extracted for the different analyses (i.e., analyses of the various phases of dynamic facial expressions).

We are confident that the differences between the mean values of the neutral phase (1 s of data) and

maximal phase (1 s of data) (which represents the experience of looking at the same face with different expressions) are adequate to make inferences regarding facial mimicry. Since the preprocessing baseline correction value would have differed slightly from the mean value for the neutral phase of dynamic facial expressions, we calculated the exact values for the epochs. Using only the mean value of the neutral phase for preprocessing baseline correction may have been sufficient for the present analysis. However, owing to the oscillating nature of the EMG data in terms of amplitude) and the issue that we aimed to address (low-frequency drift), we do not believe that the result would have differed significantly post-rectification and -log transformation if the preprocessing baseline had the same period as the psychological baseline. No current guidelines recommend preprocessing the data differently for each individual analysis.

10. Results: While the authors state that "Autism spectrum disorder (ASD) is characterized by low empathy" in the introduction (second paragraph on p.3) and repeat this again in the discussion, they could not find any significant correlations between the trait dimensions (Questionnaire scores section). How can these results be explained?

Authors' response: As noted in our response to Reviewer 1's comment 1, we have revised our description of ASD and autistic traits in the Introduction (lines 59–81) and Discussion (lines 499–557). Note that after IRIPT is removed from the statistical models, there appears to be an AQ × emotion × presentation condition interaction effect on valence ratings, which also does not support the idea that ASD is characterized by low empathy.

11. Discussion: There are some unclarities in the discussion of the results related to empathic traits (third paragraph of the discussion section, p.17). First, it could be clarified how the current study's results relate to the cited literature on mimicry and cognitive vs. emotional empathy, as well as how the ratings data is in line with Dimberg et al.'s study (only briefly mentioned). Further, the explanation with the role of the amygdala in upper facial feature representation seems a bit far-fetched, also given that the other cited studies (Sonnby-Bergström et al., Dimberg et al.) could find effects (or at least tendencies in Sonnby-Bergström et al.) for the corrugator muscle. Moreover, a restructuring of this section, starting with a comparison to previous studies on autistic traits and mimicry and then the more general discussion of ASD and responses to social stimuli in different contexts would allow for a better readability. In the limitation section, the potential influence of only using female participants could be discussed and the meaning behind the possibility "to more vividly observe the effect of social top-down regulation" should be clarified. It is further not clearly described why future studies should include "interactive activities". Last but not least, the authors close their discussion with a circular argument namely that live interactions are needed to

investigate individual differences and that individual differences should be controlled for in live interactions (similar to their abstract). As their main goal is to promote a "second-person neuroscience" to describe individual differences and they also focus on that throughout their paper, a focus on the benefits of the live context might be more appropriate.

Authors' response:

- We have included a description of how Dimberg et al.'s (2011) rating results related to our IRIEC × emotion × presentation condition interaction effects on valence and arousal ratings (lines 488–490).

- We agree that the role of the amygdala in upper facial expressions is far-fetched and have removed the text in question.

- To address previous comments, we have rewritten the Discussion section according to the autistic trait results. We now emphasize that we found no negative correlation between AQ and the level of mimicry in general, and then focus on the fact that the live effect had different effects on valence ratings and ZM responses across the AQ spectrum.

- We have included the point that the distribution of autistic traits differs considerably between males and females, in relation to the study limitation that only female participants were included.

- We have modified the sentence in the Limitations section as follows: "Spontaneous facial mimicry is regulated by sociodemographic factors such as age, gender, and socioeconomic status. Further studies are required to investigate the top-down regulatory effects of sociodemographic factors on spontaneous facial mimicry" (lines 594–597).

- We agree that the final sentences in the Conclusion are somewhat underwhelming and seemingly circular, so have removed the last two sentences from the Conclusion to focus on the benefit of the second-person approach.

Additional comments:

12. Paragraph 4 of the introduction, p.4: "The participants viewed videotaped and live-relay performances alternatively." This sentence only repeated the information from the previous sentence and can be removed.

Authors' response: We have followed this suggestion and removed this sentence.

13. Paragraph 3 of the introduction, p.4: "more emotional reactions and facial mimicry than suggested by previous data." Which previous data is referred to and in how far was the study design different?

Authors' response: As per our response to comment 5 of Reviewer 1, we agree that this expression is vague. By "previous data", we meant the results obtained using prerecorded stimuli and have changed the text to "than using prerecorded photos or videos".

14. Figure 1 caption: "to the to the prompter"

Authors' response: We are grateful for the reviewers for pointing out this error and have corrected it.

15. Second paragraph of Paradigms and procedure on p.7: As participants rated valence and arousal separately, it is on really an "affective grid rating"

Authors' response: We agree with the reviewers that this is not an affective grid rating, because we separated the valence and arousal ratings. We now refer to the ratings as valence and arousal ratings.

16. Results/Statistical analyses: The inclusion of supplemental analyses without "outlier" removal is a nice addition, especially given the to-be-expected non-normal EMG response distribution. I was personally wondering why the authors decided to cope with this problem by simply removing high EMG values. It might be a nice addition to have the benefits and potential pitfalls discussed in a few sentences so that the results without removal can be contextualized in a better way. Further, it would be helpful for the reader to not only have the number of excluded trials mentioned in the text but also the number of remaining trials for each analysis.

Authors' response:

- We would like to emphasize that, in LME model analysis, the outliers/influential data points do not necessarily denote extreme values (on initial inspection). Such concerns arose from the a priori assumption of conditional normality for regression analysis, including random effects and residuals/error terms. The outliers/influential data points identified in our LME model analysis were based on residual variance. The normality assumption can be fulfilled even if the (joint) marginal distribution of the dependent variable is not normal.

- The question of whether influential data points should be removed is still subject to debate, and we have received contradictory suggestions on this issue. Some authors suggest that all data should be treated according to Bakker and Wicherts' work [15], which only considered the independent-samples t test (which assumes a marginal normal data distribution). I It is also unclear whether we could make reliable inferences based on fixed effects if the normality assumption of the LME models was violated. Essentially, we followed Baayen and Milin's [16] approach to the exclusion of outliers (which they termed "model criticism") but identified such data points using residual diagnostics [17]. We are not in a position to suggest the optimal solution to this issue, and have presented results with and without model diagnostics to allow the reader to determine which fixed effects are most robust to assumption violations. Furthermore, we have provided a data table in the supplementary material so that the data can be reanalyzed in future in the event that concrete guidelines are proposed. We have provided more context regarding this issue in the Method section (lines 272–278).

- We have followed the reviewer's suggestion and report the number of remaining data points and participants in the Results section for the final models.

17. Third paragraph in discussion: The sentence "The level of spontaneous mimicry was related to the individual differences in empathy" seems like an unnecessary repetition.

Authors' response: We agree with the reviewer and have removed the sentence.

18. Discussion first paragraph: The overall summary could be a bit less broad and more to the point/specific to the study.

Authors' response: The first paragraph of the discussion now describes only the fact that the finding in our previous report was maintained; we describe new findings involving individual differences in the following paragraphs.

19. Third paragraph of the discussion on page 18: many concepts like "we-mode", watching eye effect,... are introduced without any explanation

Authors' response: We have followed the reviewers' suggestion and included short descriptions of these concepts (lines 493–498).

20. As there are some small language mistakes in the manuscript, independent editorial help before

publishing should be considered

Authors' response: We are grateful to the reviewers for highlighting this issue. We have sent the revised manuscript for proofreading in advance of resubmission.

Bibliography

1. Bates D, Kliegl R, Vasishth S, Baayen H. Parsimonious Mixed Models. arXiv:150604967 [stat]. 2018 [cited 27 Jul 2020]. Available: http://arxiv.org/abs/1506.04967

2. Barr DJ, Levy R, Scheepers C, Tily HJ. Random effects structure for confirmatory hypothesis testing: Keep it maximal. Journal of Memory and Language. 2013;68: 255–278. doi:10.1016/j.jml.2012.11.001

3. Chartrand TL, Bargh JA. The chameleon effect: the perception-behavior link and social interaction. J Pers Soc Psychol. 1999;76: 893–910.

4. Olszanowski M, Wróbel M, Hess U. Mimicking and sharing emotions: a re-examination of the link between facial mimicry and emotional contagion. Cognition and Emotion. 2020;34: 367–376. doi:10.1080/02699931.2019.1611543

5. Hsu C-T, Sato W, Yoshikawa S. Enhanced emotional and motor responses to live versus videotaped dynamic facial expressions. Scientific Reports. 2020;10: 16825. doi:10.1038/s41598-020-73826-2

6. Russell JA, Mehrabian A. Evidence for a three-factor theory of emotions. Journal of Research in Personality. 1977;11: 273–294. doi:10.1016/0092-6566(77)90037-X

7. Hess U, Blairy S. Facial mimicry and emotional contagion to dynamic emotional facial expressions and their influence on decoding accuracy. Int J Psychophysiol. 2001;40: 129–141. doi:Doi 10.1016/S0167-8760(00)00161-6

8. van der Schalk J, Fischer A, Doosje B, Wigboldus D, Hawk S, Rotteveel M, et al. Convergent and divergent responses to emotional displays of ingroup and outgroup. Emotion. 2011;11: 286–298. doi:10.1037/a0022582

9. Hatfield E, Bensman L, Thornton PD, Rapson RL. New Perspectives on Emotional Contagion: A Review of Classic and Recent Research on Facial Mimicry and Contagion. Interpersona. 2014;8: 159–179. doi:10.5964/ijpr.v8i2.162

10. Elfenbein HA. The many faces of emotional contagion. Organizational Psychology Review. 2014;4: 326–362. doi:10.1177/2041386614542889

11. Jack RE, Garrod OGB, Yu H, Caldara R, Schyns PG. Facial expressions of emotion are not culturally universal. Proceedings of the National Academy of Sciences. 2012;109: 7241–7244. doi:10.1073/pnas.1200155109

12. Ekman P, Friesen WV. Pictures of Facial Affect. Palo Alto, CA: Consulting Psychologists Press; 1976.

13. Ekman P, Friesen WV, O'Sullivan M, Chan A, Diacoyanni-Tarlatzis I. Universals and Cultural Differences in the Judgments of Facial Expressions of Emotion. Nebraska symposium on motivation. Lincoln, NE: University of Nebraska Press; 1972. pp. 207–283.

14. Sato W, Hyniewska S, Minemoto K, Yoshikawa S. Facial Expressions of Basic Emotions in Japanese Laypeople. Front Psychol. 2019;10: 259. doi:10.3389/fpsyg.2019.00259

15. Bakker M, Wicherts JM. Outlier removal, sum scores, and the inflation of the type I error rate in independent samples t tests: The power of alternatives and recommendations. Psychological Methods. 2014;19: 409–427. doi:10.1037/met0000014

16. Baayen RH, Milin P. Analyzing reaction times. Int j psychol res. 2010;3: 12–28. doi:10.21500/20112084.807

17. Loy A, Hofmann H. Diagnostic tools for hierarchical linear models: Diagnostic tools for hierarchical linear models. WIREs Comp Stat. 2013;5: 48–61. doi:10.1002/wics.1238
