# Supplementary material for: An investigation of the modulatory effects of empathic and autistic traits on emotional and facial motor responses during live social interactions
Source: PLoS One. 2024 Jan 9;19(1):e0290765. doi: 10.1371/journal.pone.0290765 (PMC10775989; doi:10.1371/journal.pone.0290765)
Supplement: Attachment — Submitted filename: Reply_to_Reviewers_R2_final.docx [file pone.0290765.s011.docx]

We thank the reviewers for their time and insightful suggestions. During the second revision, when checking the Cronbach’s alpha values of the IRI subscores and the AQ (Reviewer 2, comment 5), we noted that the script reading the IRI did not invert the scores of nine items, resulting in low or negative Cronbach’s alpha values. This error has been corrected. Furthermore, in response to comment 1 of Reviewer 1 on the construct of emotional empathy, we decided to better couple the psychological construct and psychometric measures. We employed the construct of emotional empathy proposed by Davis (Davis, 1980, 1983) and included both the IRI empathic concern and personal distress subscores in the statistical models to evaluate the effect of emotional empathy. Therefore, the results differ from those of the previous version.

***Reviewer #1****: I greatly appreciate the extensive changes the authors made to the manuscript. The study design is now much clearer. However, I still have some lingering concerns regarding the revised manuscript:

Introduction
1. The key terms trait emotional empathy, spontaneous mimicry, and emotional contagion require clearer definitions and should be used consistently throughout the manuscript. First, although the authors referred to “emotional empathy”, what they measured was actually “empathic concerns” i.e., “warmth, compassion, and concern toward others” (line 161-162). This is not entirely the same as the affective/emotional empathy, which involves sharing others’ emotional states, thus emotional contagion; whilst empathic concerns require not only sharing others’ emotions, but also prosocial motivation and sympathy/compassion. Second, as a result of the first point, the hypotheses were not well grounded by the literature reviewed by the authors in Introduction, which was about emotional empathy. Third, line 44-46 “Although both spontaneous mimicry and empathy have been proposed as mechanisms facilitating emotional contagion, it has also been argued that spontaneous mimicry and emotional contagion are more likely to simply co-occur” is confusing and could also be misleading. I would suggest the authors to check the Hatfield and colleagues’ chapter for the role of emotional contagion, and also the Prochazkova & Kret (2017) study for mimicry and emotional contagion.

Hatﬁeld, E., Rapson, R. L., & Le, Y. C. L. (2011). Emotional contagion and empathy. The Social Neuroscience of Empathy, 19.
Prochazkova, E., & Kret, M. E. (2017). Connecting minds and sharing emotions through mimicry: A neurocognitive model of emotional contagion. Neuroscience & Biobehavioral Reviews, 80, 99-114.*

Authors’ response:

- We have reorganized the Introduction to describe spontaneous facial mimicry, emotional contagion, emotional/affective empathy and autistic traits more thoroughly, and their relationships in the theoretical proposals and empirical data. Hatfield, and Prochazkova and Kret, are cited. We agree that some descriptions in the previous version were over-simplified. We would emphasize that the definitions and constructs of emotional empathy and emotional contagion remain very different among researchers; it is not easy to summarize these topics.
- Reviewer 1 distinguished empathic concern from emotional empathy; this view is shared by some empathy researchers. For example, Zaki categorized empathic concern as a separate dimension of motivation (Zaki, 2017). However, there seems to be no widely used trait measurement that separates traits of both emotional and motivational empathy and has been validly translated into Japanese. Returning to the construct of Davis; the cited author used two kinds of reactivity when inferring the ability to feel the emotions of others, thus both the empathic concern (which measures other-oriented empathic responses) and personal distress (which measures the self-oriented empathic responses) (Davis, 1980). We thought it is best to use both the EC and PD subscales to estimate trait emotional empathy in this revision. Thus, we now refer specifically to “trait empathic concern” and “trait personal distress” in the Results.

*2. The study aimed to examine the effects of a) empathic concerns and b) autistic traits on i) emotional contagion and ii) spontaneous facial mimicry. But not all the hypotheses for these relations were clearly outlined. For example, the hypothesis for the relation between autistic traits and emotional contagion is missing.*

Authors’ response:

The last part of the Introduction has been revised. Specifically, as the literature consistently showed that autistic traits do not affect emotional contagion, we now state that we did not expect the autistic traits to modulate emotional contagion under either live or video conditions.

*Methods
3. Some parts of the methodology remain unclear. For example, it was mentioned in line 153-155 that “Fifteen smiling and fifteen frowning clips were used for the passive viewing component; two per condition were used for the practice ratings, and four per condition were used for the actual ratings.” Only until reading later text did I realize that the 15+15 trials described here did not include the practice trials, and that the “condition” refers to the 2 (presentation) x 2 (valence) design.*

Authors’ response:

We have revised the Methods and we have moved the “Pre-recorded videos” section to after “Paradigm and procedures”. We have also revised the “Pre-recorded videos” section to include the information that two smiling and two frowning clips were employed under the positive-video and negative-video conditions of practice passive viewing trials. We hope this improves readability.

*4. Also, in line 195 “they were shown the instructions for either “Video” or “Real Person””, a previous comment on this was not addressed. The participants already knew beforehand whether the expression presented to them was pre-recorded or live. It’d be helpful if the authors can explain why it was designed like this and how this could affect the outcomes.*

Authors’ response:

As we replied to the Reviewer in the last revision, the instruction of “Video” or “Real Person” ensured that the participants knew whether an imminent stimulus was real or pre-recorded. This allowed a very clear audience effect. An effect of “the knowledge of others’ presence or being watched” has been demonstrated in previous studies using a “simulated” live interaction design (Drimalla et al., 2020; Gregory et al., 2015; Jiang et al., 2017; Koike et al., 2019; Myllyneva & Hietanen, 2015). In the absence of such instruction, participants would need to de-ambiguate or infer the live vs. pre-recorded situations by relying on subtle cues such as microexpressions or environmental inconsistencies, which do not guarantee successful detection of live performances. In the absence of instruction, it would have been difficult to validate whether participants were really prepared for live interaction, and it would have been more difficult to argue that an effect (if any) was attributable to low-level processing rather than social cognition. Particularly, when we showed prerecorded and live performance clips to naïve participants lacking such instruction during the validating rating study of our previous publication using the same paradigm, the ratings exhibited no “live effect” at all. It will be interesting to investigate the effect of low-level bottom-up processing in subsequent studies and to compare the results to those of the present study.

We have added this information to the “Paradigm and procedures” section of the Methods part (lines 324-331).

*5. The rating scales for valence and arousal were shown in the figure, but were only very vaguely and confusingly described in the text that a scale of 1 to 9 was used and a question “please rate the emotion that you feel” was asked. Yet this question is rather broad and vague, and how the scale 1 to 9 should be interpreted is unclear. This also applies to line 226.*

Authors’ response:

We agree that our description of the rating procedures was incomplete. Before the practice rating trials, participants read two slides introducing the concepts of pleasure-displeasure (valence) and arousal in reference to the Russel Affect Grid (Russell et al., 1989), and were asked to rate the emotions they experienced after seeing the facial expressions using the sequence of pleasure-displeasure (valence) and arousal. The complete details are now given in the “Paradigm and procedures” section of the Methods (lines 339-353).

We have also corrected the number of practice rating trials from eight to four (one per condition).

*6. Regarding line 251-253 “the mean value difference in EMG (ZM and CS) between the neutral (0-1 s after stimulus onset) and maximal phases (2.5-3.5 s after stimulus onset) of the dynamic facial expression”. This treatment remains unclear to me. It was described earlier [PREPROCESSING] that baseline-corrected values would be use, while here the difference between neutral and maximal phases was said to be used. Taken together, the authors are suggested to carefully check throughout their Methods section for a clearer presentation of their design.*

Authors’ response:

The baseline correction made during preprocessing sought to deal with low-frequency drift, so that the amplitude of EMG signal oscillation could be correctly retrieved. This does not affect the definition of the dependent variable. After preprocessing baseline correction, the trial-wise EMG data (concatenated) of the two EMG channels appeared as follows:

The data were then rectified (the absolute value was calculated for each point) to reflect the amplitude of EMG oscillation. This measure is highly skewed; we thus performed a natural-log transformation. Then, for the dependent variables, we calculated the (natural log-transformed) oscillation amplitude mean differences between the neutral (0-1 s after stimulus onset) and maximal (2.5-3.5 s after stimulus onset) phases.

We have revised the “EMG data pre-processing” section to include all of the abovementioned steps to improve readability.

*7. Reporting observed power is not appropriate, and also not meaningful, as it is basically a reflection of the p values. Instead, the authors are encouraged to provide an indication for how their effect sizes should be interpreted, which would be helpful for understanding the results.*

Authors’ response:

We agree with the editor and reviewers; we have removed the information.

We have added an interpretation of the partial R-squared measure: “The effect sizes for partial R-squared values in regression models are considered small at 0.01, medium at 0.09, and large at 0.25.” to the “Statistical analysis” section of the Methods (lines 442-444). The reference is (Gignac & Szodorai, 2016) and https://imaging.mrc-cbu.cam.ac.uk/statswiki/FAQ/effectSize.

*Results
8. Overall, the results are not very easy to follow. Oftentimes, it was not clearly presented what were being compared, and how one condition differs from the other. Below are some examples.
9. Line 323-324 “During the live stimuli observations, a high IRIEC level was associated with a greater discrepancy in valence ratings for positive vs. negative stimuli”: Better to also describe what is the outcome in the video condition.
10. Line 336 “The AQ slope difference … was more negative”: What does “more negative” mean?
11. Line 398-399 “Although the AQ slopes for ZM reactions were not different from zero in neither live nor video conditions, the slope was significantly more positive in the video”: First, what does "more positive" mean? Second, which interaction effect was referred to?*

Authors’ response:

The comments indicate the difficulty in delineating the nature of a two-way interaction involving one continuous variable and one categorical independent variable, and a three-way interaction involving one continuous variable and two categorical independent variables. There is no definite guideline to do this. When dealing with three-way interactions, our strategy featured visualization and evaluation using separate, two-way simple slope difference testing by categorical variables (Dawson, 2014). We simply sought to reveal the nature of the interaction.

https://biologyforfun.wordpress.com/2017/10/03/three-way-interactions-in-r/

However, the coefficients themselves are “tricky” (difficult to interpret), and we agree with the editor and reviewers that more test statistics are required to render the descriptions appropriate.

As we found no further significant three-way interaction, we needed only to clarify the two-way interactions involving one continuous and one categorical independent variable. In the revised manuscript, we visualized the interactions and reported simple slope analyses by the categorical variables (as before). In addition, we used the “pick-a-point” approach (Hayes & Matthes, 2009) and performed simple effect contrast analysis of estimated value differences between two levels of the categorical variables from the mean minus 3 SD to the mean plus 3 SD in steps of 1 SD of the continuous variable (note that the traits were already mean-centered before entry into the statistical model). This enabled us to better describe at which level of trait distribution we observe (what type of) difference in measurements of emotional contagion and facial muscular responses at two levels of the categorical variable. Indeed, this approach allowed us to correct some previous descriptions and interpretations. We hope this also improves the interpretability of the results. This information has been added to the “Statistical analysis” section of the Methods (lines 445-454).

https://stats.oarc.ucla.edu/r/seminars/interactions-r/#s4c

*12. The term “positive” and “negative” were being used for three purposes in the Results section: for describing valence, for indicating the direction of a slope, and for indicating the direction of a difference. Thus, the text is rather confusing. The authors are suggested to consider using different sentence structures to avoid using the same term for different contexts (e.g., say “higher A was related to higher B” instead of “A and B were positively related”).*

Authors’ response:

We have generally followed this suggestion in the revised manuscript.

*13. Sometimes both fixed and random effects in the final model were listed, while sometimes only random effects were outlined. Consider listing all fixed and random effects when reporting final models, or simply removing such sentences and referring to the tables, for clarity.*

Authors’ response:

All final models featured the same fixed effects but differed in terms of the random effects. We have listed only the complete fixed effects of the valence results. We did not mention (in the later Results sections) that other models featured the same fixed effects. We have now added this information.

*Discussion
14. Discussion on the effect of empathic concerns: I am not sure I am convinced by the authors’ explanation. Results showed that participants with high EC were more sensitive to the differences between different emotional expressions (positive or negative), and a live condition enhanced this effect. This finding possibly shows that the subtle signals conveyed during live interactions and that knowing the interaction partner is real, is helpful for feeling and recognizing the emotion of the partner, especially for those with higher levels of empathic concerns, who are high in sympathy and prosocial motivation. However, note that the results about arousal rating is not entirely in line with the hypotheses (higher EC was related to lower arousal ratings when the expression was negative), and thus also requires further explanations.*

Authors’ response:

After correcting the errors in the IRI readings, we found that the live effect did not modulate the interaction between the trait and emotion conditions. In a sense, we can no longer discuss the issue. However, the interpretation of the Reviewer is attractive had the effect been observed.

The corrected results still revealed a robust two-way interaction between the IRIEC and emotion conditions in terms of arousal ratings. Indeed, a higher trait empathic concern was associated with the perception of negative expressions as less arousing. We considered that negative stimuli were inherently less emotionally contagious in terms of arousal, as revealed by the significant main effect of emotion conditions on the arousal ratings. The event-related design that in a pseudorandomized manner presented participants with positive and negative expressions during all sessions. We think that this enhanced the “contrast” in the arousal contagious ability between emotion conditions; the negative condition was more neutral (or even calming) in terms of arousal contagion, especially for individuals with high trait empathic concern. Addition of a further baseline condition (true neutral facial expressions) would aid exploration of this (non-intuitive) finding in future. We have added this information to the Discussion (line 751-760).

*15. Discussion on the effect of autistic traits: I am not sure that I understand the authors’ argument that a weaker emotion contagion is relate to reduced social attention. In people with higher AQ, positive expressions were rated more positive, and negative ones rated more negative, than people with lower AQ, and this effect was even stronger in video condition than in live condition. Possibly, hypersensitivity, which is often part of the autistic traits, may play a role here, and when in live condition, they can make more "balanced" judgement. Or, it could be that the video condition, i.e., a more controlled condition, helps people with high AQ to differentiate the expressions.
16. The effect of AQ on ZM reaction: Results showed that, first, among people with lower AQ, ZM reaction in live condition was stronger than in video condition; while among people with higher AQ, no differences were between conditions in ZM reaction. Second, in video condition, higher AQ was related to stronger ZM reaction, while this association was weaker in live condition. These suggest that people with lower AQ reacted with more smiles (not necessarily mimicry as they also did so for negative expressions) when they knew the partner is real than in pre-recorded condition. But for people with higher AQ, whether in the video condition or in the live condition, they smiled to the extent similar to people with lower AQ in the live condition. In other words, participants with higher AQ did not reduce smiling in live condition, but actually increased smiling in video condition, compared to people with lower AQ. Therefore, the authors’ interpretation does not seem to hold as participants with higher AQ did not reduce their social smiling in live condition. Also, given that there is no standard for what is adequate ZM reaction, no “reduction” can be assumed.

These two papers could be helpful in interpreting empathic responses in autistic individuals:
Rieffe, C., O’Connor, R. A. G., Bülow, A., Willems. D., Hull, L., Sedgewick, F., Stockmann, L., & Blijd-Hoogewys, E. (2021). Quantity and quality of empathic responding by autistic and non-autistic adolescent girls and boys. Autism, 25(1), 199-209.
O'Connor, R. A. G., Stockmann, L., & Rieffe, C. (2019). Spontaneous helping behavior of autistic and non-autistic (pre-)adolescents: A matter of motivation? Autism Research,12, 1796-1804.*

Authors’ response (comments15 and 16):

The issues of comments 15 and 16 are related to those of comments 8-11 of Reviewer 1, thus the interpretation of two-way interactions involving one continuous variable and one categorical variable. The previous text contained certain misinterpretations, particularly in terms of two two-way interactions involving the AQ. In the current revision, additional simple-effect contrast analyses showed that individuals with high autistic traits did not differ in terms of subjective experiential valence or ZM responses between the live and video conditions. Rather, individuals low in autistic traits exhibited lower subjective valence and weaker zygomaticus responses (irrespective of the emotion condition) under the video condition compared to the live condition. We interpreted the results based on previous findings of the audience effect (in those without ASD diagnosis) on social smiling, and used the facial feedback hypothesis to link this back to the results of subjective valence. Alternatively, it may be that individuals low in autistic traits may pay less attention to social stimuli when they know they are not dealing with a real person, but we do not state this because no previous study offered this interpretation and we thus lack the required evidence.

*Other issues related to autism
17. Line 60 “Facial expressions of individuals with ASD are considered less natural”: The autistic people express emotions in their "natural" way. It is not fair to compare the naturalness of facial expressions, and to use non-autistic people as the standard.*

Authors’ response:

We did not investigate the facial expressions of individuals with ASD diagnosis. Indeed, this information is irrelevant; we have removed it.

*18. Line 62 “Spontaneous facial mimicry was also found to be reduced and delayed in the ASD population”: compared to whom?
19. Line 69 “individuals with ASD showed less social smiling and mimicry of positive facial expressions”: compared to whom?
20. Line 75 “Hermans et al. showed reduced spontaneous facial mimicry of the CS response to static angry faces in females with a high autism spectrum quotient (AQ)”: compared to whom?*

Authors’ response (comments 18 to 20):

In cited studies, individuals with ASD diagnosis were compared to those without ASD diagnosis (“neurotypical populations” in the originals). The study cited in point 20 compared females of high AQ to females of low AQ. This information has been added for clarity.

*Other issues related to clarity and readability
21. Line 34 “Dimberg was the first to demonstrate similar muscle activation patterns when seeing happy faces…”: Similar to what? Also this sentence is not easy to follow.*

Authors’ response:

The sentence was too long. We referred to “static grayscale pictures of happy or angry faces” in the end of that sentence. We have rephrased the sentence.

*22. Line 40 “increased liking for the mimicker, and facilitated prosocial behavior”: the behavior of whom?*

Authors’ response:

We referred to the prosocial behaviors of, and social bonding between, the mimicker and mimickee. The text has been revised for clarity (lines 65-69).

*23. Line 49 “…and investigated the interaction effect of trait differences and live interactions…”: It will be clearer if empathy and autistic trait are mentioned here as part of the aim. Trait differences is a very broad term.*

Authors’ response:

We have followed the suggestion (lines 108-109). Also, please note that the sentence is now followed by a subsection discussing trait emotional empathy and autistic traits.

*24. Line 63 “autonomic” should better be “spontaneous”, which is what was consistently used throughout the text.*

Authors’ response:

We have followed the suggestion.

*25. Line 71-72 “Recent evidence indicated that there is no difference in emotional empathy or emotion contagion between the ASD and neurotypical population”: This sentence does not connect well to the rest of the paragraph. It is in-between the paragraphs on facial mimicry. Therefore, hard to follow and grasp what is the message here. Consider moving it to the beginning of the paragraph, or to the end of the next paragraph.*

Authors’ response:

We have moved the information to the end of the paragraph, , thus after the description of the work of Press et al. (Press et al., 2010) (lines 181-184). The next paragraph discusses previous findings on the effects of the autistic traits in populations without ASD diagnosis.

*26. Line 101-104: This is an important sentence about the hypotheses, but unfortunately very hard to read.*

Authors’ response:

We have divided the overtly long sentence into shorter sentences.

*27. Line 165 “i.e., those with and without autism”: Better change “autism” to “a diagnosis of autism” for clarity.*

Authors’ response:

We have adopted this suggestion (“individual/population with/without a diagnosis of ASD”) throughout the revised manuscript.

*28. Line 247 “Statistical analyses of live interaction data”: why only live data is mentioned here, while both presentation modes are actually in the models?*

Authors’ response:

We have changed the section title to “Statistical analyses” to avoid confusion.

*29. The authors are suggest to ensure consistent use of terms throughout the manuscript. For example, currently “valence”, “emotion”, and “emotion categories” were used interchangeably.*

Authors’ response:

We have thoroughly examined this issue and made adjustments whenever appropriate. For example, we now use “emotion conditions” to describe the categorical variable of the study design.

*30. The manuscript would benefit from English language editing.*

Authors’ response:

Both the previous and the current revised texts and replies to reviewers were sent for English proofreading. However, English-language editors vary in terms of style. We have mentioned the reviewer’s concern to our proofreading service.

The English in the current revised manuscript has been checked by at least two professional editors, both native speakers of English. For a certificate, please see:

<http://www.textcheck.com/certificate/AkoSUv>

***Reviewer #2****: Overall, it is evident that the authors acknowledged and implemented the feedback. There are, however, still some points that require further improvement.

Major concern:
1. Results: It is appreciated that the authors implemented the suggested comparison between presentation conditions (e.g. positive-live vs. positive-video) to their Results section. However, I was puzzled by the interpretations that were drawn from the examination of slope differences. First, even though some slope differences did not reach significance (e.g. IRIEC slope difference in live vs. video for both arousal ratings of positive and negative stimuli, lines 357 – 361), the authors still interpreted this as e.g. more/less arousing perception of the stimuli in the live condition.
Secondly, they described slope differences to be greater in one condition vs. the other (e.g. IRIEC slope difference in positive vs. neutral in the live vs video condition, lines 353-357) even though no statistical test supporting this claim is reported, i.e. they were most probably only referring to numerical values or graphs.
Thirdly, while slope differences might be informative to compare between presentation conditions, they do not yield any information on whether there is indeed a significant association between e.g. the IRIEC and arousal ratings in a specific condition. As the coding of the predictors is not specified in the model description (e.g. whether they are sum coded or which level acts as a reference level), it is impossible to retrieve this information from the model tables. As simplest solution, I would suggest to report whether the individual slopes for each combination of the factor levels, e.g. positive-live, are significantly different from 0 in the three-way interaction with each of the trait variables and, as a next step, to look at slope differences. Only the significant findings should then be further interpreted which should, if necessary, lead to adjustments in the Abstract, the Result section and the Discussion section. The outlined concerns also apply to the reporting of the results in the Supporting Information.*

Authors’ response:

- This comment again reflects the difficulty of interpretating interactions involving both continuous and categorical variables (see comments 8–11 of Reviewer 1). Please see our reply above. We agree that more statistical testing was required to properly describe the nature of the interactions. We now include visualizations, simple slope analyses per categorical level (Dawson, 2014), and the “pick-a-point” approach (Hayes & Matthes, 2009) to perform simple-effect contrast tests at mean-centered continuous variable levels from –3 SD to 3 SD in steps of 1 SD while comparing two levels of the categorical variables; we use the Šidák method to correct for multiple comparisons.

https://stats.oarc.ucla.edu/r/seminars/interactions-r/#s4c

- We thank Reviewer 2 for pointing out that the reference levels of the categorical variables were not given in the previous manuscript. We have added this information to the “Statistical analysis” section of the Methods. The reference level for the emotion condition is “Negative” and that for the presentation condition is “Video” (lines 416-418).

*2. Lastly, the addition of a post-hoc power analysis is highly criticized and I would recommend to exclude it (see: [https://daniellakens.blogspot.com/2014/12/observed-power-and-what-to-do-if-your.html](https://daniellakens.blogspot.com/2014/12/observed-power-and-what-to-do-if-your.html); [https://data.library.virginia.edu/post-hoc-power-calculations-are-not-useful/](https://data.library.virginia.edu/post-hoc-power-calculations-are-not-useful/); [https://link.springer.com/article/10.1007/s12144-018-0018-1](https://link.springer.com/article/10.1007/s12144-018-0018-1)).*

Authors’ response:

We agree with the editor and Reviewers; we have removed this information.

*Minor concerns:
3. Introduction: The introduction benefits from the clarification of concepts and addition of research on autistic traits. The authors might reconsider starting the first paragraph in the introduction with the same words as the abstract.*

Authors’ response:

We have restructured and expanded the Introduction to overview the objective of the present study first, and then more thoroughly summarize the constructs that we cover and the relationships among them. The Abstract has also been rewritten to reflect the corrected results (Reviewer 2 comment 5).

*4. Methods (Stimuli validation) + Discussion: It was great to see that the authors put a lot of effort in examining whether the negative/angry expressions could potentially be confused with sad expressions. As this was not one of the main goals of the study, I was surprised to find a quite long paragraph on it in the Discussion section. Despite the fact that this paragraph contains very interesting information, the discussion might become more concise ad focused if the issue of potential misinterpretations is only briefly touched upon, e.g. in the limitations.*

Authors’ response:

We have shortened the relevant paragraph in the Discussion and moved it to before the limitations. The paragraph explains that the issue of ambiguity in negative expressions is inherent in the culture wherein we conduct our work. It is important for readers to bear this in mind when interpreting our results.

*5. Methods/Results: It would be great to have a measure of reliability of the AQ and the IRIEC scale (e.g. Cronbach’s alpha) in either the Methods section or the Results section (when the scores in the sample are reported), as commonly done in research with questionnaire scores.*

Authors’ response:

We thank the reviewers for pointing out the issue with Cronbach α. We checked the values for the IRI and subscores, and found unusually low or negative values, which prompted us to recheck the questionnaire data pipeline. We discovered that the script reading the IRI and the subscore data did not invert nine of the IRI questions. This issue has been corrected; the results now differ.

After correction of the erroneous IRI inversions, the standardized Cronbach α values were 0.764 for the IRIEC, 0.833 for the IRIPD, and 0.747 for the AQ. This information has been added to the “Questionnaire scores” section of the Results (line 457-460).

*6. Discussion: The readability of the Discussion section would benefit from integrating information regarding specific findings and related literature, which currently seems repetitive in some cases. More specifically, paragraph 2 and 3 in the Discussion section both contain information about all findings related to the IRI. For example, the first sentence of the third paragraph (lines 473 – 474) is a mere repetition of the second sentence of the second paragraph (lines 462 – 465) in the Discussion section. The same applies to the fourth and the third paragraph regarding autistic traits. For example, the correlation between autistic traits and ZM activity, independent of emotion condition, is referred to twice, once in the third paragraph (lines 500 – 502) and the fourth paragraph (lines 520 – 521).*

Authors’ response:

The discussion has been extensively re-organized and revised to accommodate the corrected results (Reviewer 2, comment 5). Care has been taken to avoid repetition.

*Small comments:
7. - Line 30: it is unclear what the authors refer to with “these findings”. They could use our findings” instead.*

Authors’ response:

We have followed the suggestion.

*8.- Lines 37-40: the phrasing of this sentence suggests a causal role of mimicry in all the listed processes which seems a strong claim*

Authors’ response:

Also in response to comment 1 of Reviewer 1, we have revised and expanded the summaries of the effects of spontaneous mimicry and also present alternative theoretical proposals on how other social modulatory factors may affect spontaneous mimicry (line 50-60).

*9. - Figure 4: It is unclear why the results for the AQ and the valence ratings are visualized first (A) whereas the results for the IRIEC and the valence ratings are reported first in the Results section. Further, the figure title in the caption should also include modulatory effects of autistic traits.*

Authors’ response:

The former Fig. 4 would be more attractive if, for both rows, the red-blue legend of the emotion conditions appeared on the right side. However, as the three-way interaction disappeared after correcting the IRI errors, the issue no longer exists.

**Bibliography**

Davis, M. H. (1980). A Multidimensional Approach to Individual Differences in Empathy. *JSAS Catalog of Selected Documents in Psychology*, *10*, 85.

Davis, M. H. (1983). Measuring Individual-Differences in Empathy—Evidence for a Multidimensional Approach. *Journal of Personality and Social Psychology*, *44*, 113–126. https://doi.org/10.1037/0022-3514.44.1.113

Dawson, J. F. (2014). Moderation in Management Research: What, Why, When, and How. *Journal of Business and Psychology*, *29*(1), 1–19. https://doi.org/10.1007/s10869-013-9308-7

Drimalla, H., Scheffer, T., Landwehr, N., Baskow, I., Roepke, S., Behnia, B., & Dziobek, I. (2020). Towards the automatic detection of social biomarkers in autism spectrum disorder: Introducing the simulated interaction task (SIT). *Npj Digital Medicine*, *3*(1), 25. https://doi.org/10.1038/s41746-020-0227-5

Gignac, G. E., & Szodorai, E. T. (2016). Effect size guidelines for individual differences researchers. *Personality and Individual Differences*, *102*, 74–78. https://doi.org/10.1016/j.paid.2016.06.069

Gregory, N. J., Lόpez, B., Graham, G., Marshman, P., Bate, S., & Kargas, N. (2015). Reduced Gaze Following and Attention to Heads when Viewing a “Live” Social Scene. *PLOS ONE*, *10*(4), e0121792. https://doi.org/10.1371/journal.pone.0121792

Hayes, A. F., & Matthes, J. (2009). Computational procedures for probing interactions in OLS and logistic regression: SPSS and SAS implementations. *Behavior Research Methods*, *41*(3), 924–936. https://doi.org/10.3758/BRM.41.3.924

Jiang, J., Borowiak, K., Tudge, L., Otto, C., & von Kriegstein, K. (2017). Neural mechanisms of eye contact when listening to another person talking. *Social Cognitive and Affective Neuroscience*, *12*(2), 319–328. https://doi.org/10.1093/scan/nsw127

Koike, T., Sumiya, M., Nakagawa, E., Okazaki, S., & Sadato, N. (2019). What Makes Eye Contact Special? Neural Substrates of On-Line Mutual Eye-Gaze: A Hyperscanning fMRI Study. *Eneuro*, *6*(1), ENEURO.0284-18.2019. https://doi.org/10.1523/ENEURO.0284-18.2019

Myllyneva, A., & Hietanen, J. K. (2015). There is more to eye contact than meets the eye. *Cognition*, *134*, 100–109. https://doi.org/10.1016/j.cognition.2014.09.011

Press, C., Richardson, D., & Bird, G. (2010). Intact imitation of emotional facial actions in autism spectrum conditions. *Neuropsychologia*, *48*(11), 3291–3297. https://doi.org/10.1016/j.neuropsychologia.2010.07.012

Russell, J. A., Weiss, A., & Mendelsohn, G. A. (1989). Affect Grid: A Single-Item Scale of Pleasure and Arousal. *Journal of Personality and Social Psychology*, *57*(3), 493–502. https://doi.org/10.1037/0022-3514.57.3.493

Zaki, J. (2017). Moving beyond Stereotypes of Empathy. *Trends in Cognitive Sciences*, *21*(2), 59–60. https://doi.org/10.1016/j.tics.2016.12.004
