# Supplementary material for: An investigation of the modulatory effects of empathic and autistic traits on emotional and facial motor responses during live social interactions
Source: PLoS One. 2024 Jan 9;19(1):e0290765. doi: 10.1371/journal.pone.0290765 (PMC10775989; doi:10.1371/journal.pone.0290765)
Supplement: Attachment — Submitted filename: Reply_to_Reviewers_R3_final.docx [file pone.0290765.s012.docx]

*The Editor: I also have concerns about the simple effects contrasts using -3 to 3 SD as stratification levels. Given the overall sample size of this study, what are the cell sizes for such an analysis? If there is only one or two participants in some of the extreme cells, how can the estimates be considered stable?*

Author's response:

The simple effect contrasts were estimated using the emmeans package. The emmeans package uses a reference grid approach to estimate/predict marginal means based on the fitted model [1], and not directly on the data. Therefore, the actual cell size should not be a concern (<https://cran.r-project.org/web/packages/emmeans/vignettes/basics.html>). We have added this information to the Statistical analysis section (L414-417).

*Reviewer #1: I appreciate the authors' effort in revising the manuscript. In this revised version, the analyses and the results were presented with clarity, and I believe the results are interesting to the readers of PLOS ONE. Yet, given that a large proportion of the text has been rewritten, I do still have some concerns regarding the readability of this manuscript. I will give some examples below.

1. Many theories/studies were listed to describe the literature or interpret the results, yet oftentimes only until the last sentence did the authors mention which is the one they follow, yet still without explicitly explained reasons for the choice. Thus, the text became less concise and with contradictory views that are difficult to follow. There are indeed different views on how empathy and emotional contagion can be defined in the literature; it is thus of importance that the authors are clear about which view they take and the reason for their choice. The authors are suggested to focus on the theoretical framework of their study and be clear about how it supports the hypotheses and study design.

For example, in the Intro, only until the last sentence under "Spontaneous facial mimicry and emotional contagion" did the authors mention that they considered facial mimicry and emotion contagion as parallel processes (despite their co-occurrence often observed), without a clear reason for why this was adopted in this study. The authors may already mention the importance of the social context earlier in the text, as this appears to be the support for why the authors chose to see them as two processes rather than one. Similarly in Discussion, these two processes were often discussed in a mixed manner. For example, the "general propensity to emotional contagion" was used to explain the effect of trait personal distress on ZM mimicry, without clear reasoning.

Also, under "Trait emotional empathy and autistic traits" (Intro), task design was mentioned to play a role in the effect of autistic trait. This is an important point, yet unfortunately came too late and not explained clearly. The authors did not mention anything about the task design in the previous description of these studies, so it is unclear how the Press et al study is different from the McIntosh and Drimalla studies, in terms of the attentional requirement in their tasks. Instead, many other features about those studies were described, yet their relevance to the current studied topic remained unexplained.

Moreover, whether the task could induce a clear "audience effect" was a focus in the task design of this study, yet this was not mentioned as part of the argument in the Intro when the hypotheses were defined.*

Author's response:

- Based on the requests of the editor and reviewers, we have shortened the Introduction to include only information directly relevant to the present study and reviewers’ concerns. Conflicting theoretical proposals regarding the mechanisms of emotional mimicry have been presented in a series of recent articles [2–5], and we cite and refer interested readers to these articles for further details. We have stated that spontaneous mimicry involves multilevel appraisal and response generation mechanisms that cannot be entirely accounted for by primitive emotional contagion, as noted by Scherer [5]. Therefore, we consider spontaneous facial mimicry and emotional contagion as separate processes that often co-occur. Despite considering the Social Regulator or Communication Accommodation perspectives, we did not negate the primitive emotional contagion account, according to which spontaneous mimicry is involved in emotional contagion. We have noted that this provides an indirect explanation, where no previous report has indicated that IRIPD modulates facial mimicry (L734-735, 744-745).
- In the Introduction, we have revised and shortened the paragraph that reviews studies investigating ASD and spontaneous mimicry, and moved Press et al.’s explanation of inconsistent findings to an earlier part of the paragraph (L108-111)). The main intention was to show that a clear relationship between ASD diagnosis and levels of spontaneous mimicry has not been demonstrated empirically. Moreover, the task design has been proposed as an influential factor. The present study also involved passive observation of facial stimuli, which should have led to reduced facial mimicry in high-AQ participants, according to Press et al.’s proposal, yet no such effect was found in our results. Therefore, the proposed task effect may not be relevant to the present study, although it remains as a possible explanation for the inconsistency in the previous findings. We have further discussed the absence of reduction in facial mimicry in participants with high AQ scores in the Discussion (L703-713).
- We have added information about the audience effect to the Introduction (L144-150).

*2. Sometimes the text deviates from the main topic, or the topic switched. For example, in the Intro, the authors used one paragraph to describe the IRI, which should better be described in Methods to avoid repetition. In the Intro, the authors are suggested to focus on explaining the constructs. In the last paragraph under "Trait emotional empathy and autistic traits", the focus is about autistic traits and facial mimicry, yet in the concluding sentence the topic switched to trait emotional empathy and emotional contagion and mimicry. Also for example in the Discussion, in a paragraph that is about autistic people or people with high AQ, sometimes the topic switched to non-autistic people in-between the text. The authors may e.g., discuss the patterns of autistic vs. non-autistic (or high vs low AQ) in separate paragraphs to help readers follow the arguments.

Adding sub-headings may help readers follow the different topics discussed in the Discussion.*

Author’s response:

- As suggested, we have moved the description of IRI from the Introduction to the Methods section (L232-241).
- We have removed the part of the concluding sentence in the Introduction about autistic traits and facial mimicry, which referred to emotional empathy.
- We have moved the part discussing “the consistent pattern of AQ × presentation condition interaction in terms of subjective valence and the extent of smiling” to the first paragraph of that sub-section in the discussion (L659-671).
- We have added sub-headings to the Discussion.

*3. There are several repetitions throughout the manuscript. For example, in Intro under "Trait emotional empathy and autistic traits", the distinction between facial mimicry and emotional contagion and the role of the social context repeated what has been mentioned in the previous section. Also, the summary of findings in the 1st paragraph of the Discussion is rather vague. It is suggested to directly tell what the effects are (rather than only saying that there're modularly effects towards different conditions) to avoid repetitions later on and thus be more concise. Another example is in the paragraph in Discussion starting with "We found no reduction in facial mimicry...", which repeated the previous paragraph. It may also be an idea to focus the discussion on the findings that did not meet the expectations, as the ones that met the expectations and were in line with previous studies were already explained in the Intro.*

Author's response:

- As suggested, we have removed repetitious text and shortened the Introduction. specifically, we merged the “Spontaneous facial mimicry and emotional contagion” and “Trait emotional empathy and autistic traits” sub-sections. The merged sub-section started by introducing Davis’ construct of empathy, spontaneous facial mimicry, and how they are associated with emotional contagion.
- We have revised the sentence in the first paragraph of the Discussion regarding the presentation condition × autistic trait interaction accordingly (L647-653).
- We modified the Discussion to clarify the information regarding “participants with high AQ showed the same **social smile** in both presentation conditions” and “no reduction in **facial mimicry** among participants with higher AQ scores”. We specified that the social smile occurs regardless of emotional condition, but facial mimicry refers to consistent muscular (ZM/CS) responses to the models’ facial expressions according to the emotional condition. Therefore, we have discussed these two issues in two consecutive but separate sub-sections, and added sub-headings to avoid confusion. (L692-701, 703-713).

*4. The bridge between sentences was sometimes unclear, or two sentences were bridged in a not very logical way. For example, in the 1st paragraph of the Intro "Trait emotional empathy enhances.... However....", the "However" here should better be changed to "Moreover". In the 1st paragraph under Spontaneous facial mimicry and emotional contagion, it was mentioned that "as most previous studies did not provoke antagonistic responses," which contradicts a previous sentence about CS responses towards angry faces. In Methods, it was mentioned that "because some people might consider that the models' negative expressions...", yet in the text above, it was actually unclear that the models were instructed to express anger, and thus this sentence came as a surprise.

In the 1st paragraph of the Discussion, it was mentioned that "we did not find any evidence... that a live interaction design affected the modularly effects...", which immediately contradicts the next sentence where the effects on ZM responses and subjective valence were described. Also in the Discussion, although one important result was that the participants with high and low AQ did not differ in ZM/CS responses, this finding was not mentioned anywhere, leading to difficulty in understanding how the present study contradicted with the Drimalla study.

These sentences should be reformulated for clarity and a better flow.*

Author's response:

- We have changed the “However” to “Moreover” in the first paragraph according to the reviewer’s suggestion (L42).
- The sentence “as most previous studies did not provoke antagonistic responses…” has been deleted to shorten the Introduction (see response point 1 to Reviewer 1’s point 3). However, participants had a (simulated) antagonistic conflict with the person in the video whom they later observed in Mauersberger et al.’s paradigm [6]. The referred antagonistic context did not involve only the presentation of a frowning picture of an irrelevant person to the participant.
- We have clarified that the models were instructed to make happy and angry faces in the “Pre-recorded videos” section and referred the readers to Fig 2 (L320-322).
- We have clarified our interpretation of the findings in the Discussion. We did not detect a three-way interaction, but identified a two-way AQ×presentation conditions interaction effect on the ZM and valence ratings. The two-way interaction was not considered indicative of modulatory effects of the presentation conditions on facial mimicry or emotional contagion, because this would require the modulation to be consistent with the emotional condition, which would only be the case in a three-way interaction involving the emotional condition (L649-653). We have discussed social smiles (for the two-way interactions, L659-701), and the lack of a modulatory effect of autistic traits on facial mimicry (L703-713).
- We added in the sub-section “Social smile, reputation management, and autistic traits” that “in the simple slope analysis of the AQ × presentation condition interaction, the AQ slopes of the ZM reactions were no different from zero under either live or video conditions, suggesting that participants with high and low AQ did not differ in ZM responses,” which contrast with Drimalla et al. who reported fewer social smiles in participants with an ASD diagnosis (L672-677).

*Other issues:

- Throughout the manuscript, the expression "individuals with ASD diagnosis compared to 'others'" was often used, which should better be revised. The authors are suggested to specify the control group used by the study cited (e.g., changed to "compared to those without an ASD diagnosis" if this was the case for the study cited) for better clarity and for a more inclusive language use.*

Author’s response:

We followed this suggestion on the issue of inclusive language.

*- From Methods and Results, it appears that participant gave their subjective ratings after all the passive-viewing trials, and that passive-viewing and subjective ratings had different numbers of trials (60 vs 16). What's less clear to me is whether this means that the participants had to rate an expression that they had already seen for a second time. The effect of such a design should be discussed.*

Author's response:

- We added to the “Paradigm and procedures” subsection that the relative proportions of passive viewing (EMG) trials and rating trials align with our prior assumption about the effect size and signal-to-noise ratio of spontaneous facial mimicry and ratings. The EMG would have a smaller effect size and lower signal-to-noise ratio than ratings, thus requiring significantly more repetitions per participant. However, participants might experience a loss of attention or interest if the passive viewing period persists for too long. With 60 EMG and 16 rating trials, the total testing time per session was less than 1 hour (L270-276).
- We also added to the “Paradigm and procedures” subsection the information that no rating was requested in the passive viewing trials to detect spontaneous, automatic facial mimicry without top-down emotional processing, as in previous studies [7,8], and to prevent motion-related EMG artifacts associated with head and hand movements (L290-292).
- For the video conditions, the clips (more than 20 per emotional condition) used in the passive viewing trials (15 per emotional condition) and rating trials (4 per emotional condition) were not repeated. We added this information to the “Paradigm and procedures” subsection. The live conditions were always newly performed by the models (L329-331).
- Because the models consistently performed the smiling and frowning, participants might have experienced that they viewed passively the model smiling or frowning 60 times and then asked for ratings for another 16 trials. The sequential effect of “passive viewing 🡪 ratings” might have caused participants to be more adapted and desensitized to the stimuli during rating trials. This might have increased the hurdle of detecting modulatory effects relevant to emotional contagion. We discussed this issue in the subsection “The sequential effect of procedures” as a limitation (L817-824).

*- A brief discussion on the use of model diagnostics and the exclusion of extreme data would further help readers understand and interpret the results.*

Author's response:

We have added a paragraph to the Limitation sub-section to discuss how decisions regarding model diagnostics affected the results (L826-839). As new methods for linear mixed model diagnostics are currently being developed, and concrete guidelines have not been formulated, we encourage readers to consider the reported results both with and without model diagnostics under this context, and to reanalyze the deposited data as new model diagnostic algorithms and model treatment methods emerge.

*Reviewer #2: It is obvious that the authors put a lot of effort in addressing the reviewers’ comments and the manuscript clearly improved. Nevertheless, I still have some concerns which should be addressed before publication:

General concerns:
1. I appreciate that the authors aimed to clarify the relationship between the diagnosis ASD and autistic trait levels. However, the phrasing “the autism spectrum is a continuum that covers the entire population” (line 185 and line 293) seems to suggest that “spectrum” would reflect a continuum whereas it actually reflects the variability (multiple dimensions) within the ASD diagnosis. In these lines, the usage of “spectrum” when referring to variations in IRIEC traits (line 747, “along the spectrum of the IRIEC”) seems rather inappropriate.*

Author's response:

As suggested, we have changed the word “spectrum” to “along the range of the IRIEC” (L717).

*2. The authors already become more consistent in using specific terms for specific concepts. This is, however, not always the case for “emotion contagion” (e.g. line 94, line 107, line 747) and “emotional contagion” (e.g. line 25, line 71, line 771).*

Author's response:

As suggested, we have revised the manuscript thoroughly to ensure that “emotional contagion” is used throughout.

*3. In multiple instances, the authors claim that there was no effect of autistic traits on emotional contagion or spontaneous mimicry (abstract: lines 31-32, discussion: line 738). I assume that they refer to the lack of a significant interaction between emotion condition and autistic traits (and not the lack of a main effect of autistic traits) here, but this should be clarified to the reader.*

Author's response:

As suggested, we have clarified this text by explicitly mentioning that there was “no two-way interaction between AQ and emotional conditions” (L33-34, 703-704).

*Abstract:
4. As valence itself is a neutral word, “higher subjective valence” (line 26) should be defined in its directionality, i.e. more positive.*

Author's response:

We have implemented the reviewer’s suggestion (L27).

*5. I would recommend the authors to already clarify in the abstract that emotional contagion is measured via subjective experiential ratings, i.e. by mentioning it in parentheses in line 21.*

Author's response:

As suggested, we have described in the Abstract that we used subjective experiential valence and arousal ratings to estimate emotional contagion, and used ZM and CS EMG measurements to estimate spontaneous facial mimicry (L21-22).

*6. Since especially the contrast in the presentation effect between e.g. individuals low in autistic traits vs. high in autistic traits is interesting, I personally would not say that live interaction designs are especially valuable for individuals low in autistic traits (line 34, similar trait empathy).*

Author's response:

We have implemented the reviewer’s suggestion and changed the last sentence in the abstract to be more neutral: “Our findings imply that studies using live interactions could yield valuable insights when investigating social behaviors in the context of trait emotional empathy and autistic traits.” (L34-36)

*Introduction:
7. It is unclear why findings on modulated facial mimicry by autistic trait levels which are reported in the respective paragraph would “imply a positive correlation between trait emotional empathy and both emotional contagion and spontaneous facial mimicry” (lines 197-198). Further, in the next sentence, it is not very clear why and how the authors relate findings with regard to emotional contagion and facial mimicry to each other, given that they claim to look at them as separate constructs. This might, however, only be due to the way in which it is phrased.*

Author's response:

We have removed the sentence mentioning the trait emotional empathy and focused on the effect of autistic traits. We also revised the following sentence, which provides a summary of the current understanding of ASD diagnosis-facial mimicry and ASD diagnosis-emotional contagion relationships (L130-133).

*Results:
8. I would recommend the authors to be careful when reporting significant main effects in the context of a significant interaction in which the main effect is involved (e.g. lines 614-615) since they might not be apparent in the absence of the interaction.*

Author's response:

We thank the reviewer for reminding us of this, and have revised the Results section accordingly: significant main effects involved in an interaction effect are no longer reported. We have added this information to the “Statistical analysis” section in the Methods (L417-420).

*9. In line with Reviewer 1’s comment on the previous revision, the authors could be more precise in their phrasing. For example, they refer to “significantly more ZM responses” in the description of their results while not using count data but continuous variables (strength of activation) in their analyses.*

Author's response:

As suggested, we now use “stronger/weaker” to describe muscular responses.

*Discussion:
10. As the results greatly differ between the model fits including all data vs. remaining data after model diagnostics, the robustness of effects should be discussed.*

Author's response:

We have included a paragraph in the limitation sub-section discussing how decisions regarding model diagnostics have affected the results (L826-839). As new methods for linear mixed model diagnostics are currently being developed, and because no concrete guidelines have been formulated, we encourage readers to consider the reported results both with and without model diagnostics under this context, and to reanalyze the deposited data as new algorithms and treatment methods emerge.

*- Given the introduction and hypotheses, I would further appreciate if the following points would be touched upon in the introduction:
o Why were no modulatory effects of any trait dimension found in the model on the corrugator data? It is only very briefly mentioned that the corrugator is generally less mimicked.*

Author's response:

Indeed, contrary to Hermans et al. [9], we did not observe an AQ × emotional condition interaction effect on the CS responses. One possible explanation for this is that Hermans et al. included 18 participants in their high AQ group, and 16 in their low AQ group, with extremely high and low AQ scores, respectively, among a sample of 366 participants. In the present study, we did not focus on the extremes of the AQ spectrum. Therefore, the effect would have been much smaller than that in Hermans et al. Similarly, Dimberg et al. selected the 72 highest and 72 lowest QMEE-ranked participants from a large sample (> 500 participants) for their analysis of trait emotional empathy and its interaction with emotional condition. In contrast, we did not select participants with extremely high or low trait emotional empathy, which might have been required for these interaction effects to be detected. Furthermore, such effects might not be linear in an average population. Using our data as a pilot study for future studies, simulation using *simr* showed no increase in power with a sample size of up to 200 for these two-way interactions.

We have added this information to the Limitations section of the Discussion (L805-815).

*o Even though it is clearly mentioned in the abstract, there is no potential explanation on why the presentation condition had no impact on the modulatory effect of empathy on contagion and facial mimicry. As this was one of the primary hypotheses, I would have appreciated a short speculation.*

Author's response:

We simulated the required sample size to detect three-way interaction effects in the population using the R package *simr* for a sample size of up to 200 participants.

- Regarding the valence ratings, a three-way interaction involving IRIPD would achieve 70% power with a sample size of 200, while that involving AQ would reach 60% power.
- Regarding the arousal ratings, a three-way interaction involving IRIPD would achieve 80% power with a sample size of 150.
- Regarding the ZM responses, a three-way interaction involving IRIEC would achieve 70% power with a sample size of 150, and the power would not increase with a sample size of 200. A three-way interaction involving AQ would achieve 70% power with a sample size of 180, and the power would not increase with a sample size of 200.

The simulation results suggested that our ability to make inferences regarding three-way interactions was limited. Also, any effect size was likely to be very small. A much larger sample size would be required to reliably detect such effects. We have added this information to the Limitations section of the Discussion (L792-804).

*o While arguing that emotional contagion and facial mimicry should be regarded as separate constructs in the introduction, the authors relate them to each other again in the discussion of the findings on modulatory effects of autistic traits (lines 728-737). Given that the relationship between emotional contagion and mimicry was quite extensively reviewed in the introduction and their separation strongly favored, the authors might consider referring back to this discussion, given their new findings and interpretation.*

Author's response:

- That section discusses the similar pattern of results of the valence ratings and ZM responses in the context of the two-way AQ × presentation condition interaction. We discussed the extent of the social smile, rather than facial mimicry, which involves emotional condition-specific responses (ZM in the positive condition and CS in the negative condition). To avoid confusion, we have added sub-headings for differentiation from the later discussion concerning the fact that AQ does not modulate facial mimicry.
- In our review of the literature on the relationship between emotional contagion and emotional facial mimicry, we included theories on primitive emotional contagion and social regulator perspectives. We cite Scherer’s comments regarding the ongoing theoretical conflicts [5]: facial mimicry is subject to multilevel regulation, and emotional contagion alone cannot fully account for facial mimicry, as made clear in the Introduction of the revised manuscript (L85-87). We did not rule out a possible influence of primitive emotional contagion; specifically, the “facial feedback hypothesis”, which is supported by empirical data from a meta-analysis (L664-671).

*11. While the “audience effect” (line 703 and following) is surely relevant in research on social interactions, the presence of others (third person perspective) is qualitatively different from interacting with another person (second person perspective) as it occurred in this study. Therefore, I would be careful to claim that stronger smiles are displayed in the live condition due to an “audience effect”.*

Author's response:

We agree that the present second-person perspective paradigm involves more than the mere presence of others, and should have led to greater social preparedness. We have added this information to the “Social smile, reputation management, and autistic traits” sub-section following the discussion of the function of social smiles (L688-691).

*12. In some parts, the authors might consider being more concise, i.e.
- “smiled equally” (line 726): as ZM activity is very close to 0, it could as well be said that they did not smile in both conditions. I would therefore try to phrase it more neutral.
- “rather calming”(line 756): were the ratings of the negative stimuli generally indeed <5 (on the calming side of the affect grid) or were they simply less arousing than the positive stimuli?*

Author's response:

We have changed the first sentence (L699-701) to “participants with high AQ values showed comparable levels of smiles (or no smiles) when viewing video stimuli and real persons.”

We have also changed the second sentence (L726-727) to “…negative stimuli, in contrast to positive stimuli, were less arousing or more calming, irrespective of presentation condition.”

**Reference**

1.	Searle SR, Speed FM, Milliken GA. Population Marginal Means in the Linear Model: An Alternative to Least Squares Means. Am Stat. 1980;34: 216–221. doi:10.1080/00031305.1980.10483031

2.	Hess U, Fischer A. Emotional mimicry as social regulator: theoretical considerations. Cogn Emot. 2022; 1–9. doi:10.1080/02699931.2022.2103522

3.	Kret ME, Akyüz R. Mimicry eases prediction and thereby smoothens social interactions. Cogn Emot. 2022;36: 794–798. doi:10.1080/02699931.2022.2110452

4.	Bernhold QS, Giles H. Emotional mimicry: a communication accommodation approach. Cogn Emot. 2022;36: 799–804. doi:10.1080/02699931.2022.2109599

5.	Scherer KR. The mystery of emotional mimicry: multiple functions and processing levels in expression imitation. Cogn Emot. 2022;36: 781–784. doi:10.1080/02699931.2022.2115614

6.	Mauersberger H, Hess U. When smiling back helps and scowling back hurts: individual differences in emotional mimicry are associated with self-reported interaction quality during conflict interactions. Motiv Emot. 2019;43: 471–482. doi:10.1007/s11031-018-9743-x

7.	Sato W, Yoshikawa S. Spontaneous facial mimicry in response to dynamic facial expressions. Cognition. 2007;104: 1–18. doi:10.1016/j.cognition.2006.05.001

8.	Fujimura T, Sato W, Suzuki N. Facial expression arousal level modulates facial mimicry. International Journal of Psychophysiology. 2010;76: 88–92. doi:10.1016/j.ijpsycho.2010.02.008

9.	Hermans EJ, van Wingen G, Bos PA, Putman P, van Honk J. Reduced spontaneous facial mimicry in women with autistic traits. Biol Psychol. 2009;80: 348–353. doi:10.1016/j.biopsycho.2008.12.002
