# Supplementary material for: An investigation of the modulatory effects of empathic and autistic traits on emotional and facial motor responses during live social interactions
Source: PLoS One. 2024 Jan 9;19(1):e0290765. doi: 10.1371/journal.pone.0290765 (PMC10775989; doi:10.1371/journal.pone.0290765)
Supplement: Attachment — Submitted filename: Reply_to_Reviewers_R4_V1.docx [file pone.0290765.s013.docx]

*Reviewer #3: I would like to thank the editor for the invitation to read the article “An Investigation of the Modulatory Effects of Empathic and Autistic Traits on Emotional and Facial Motor Responses during Live Social Interactions”.*

*The study assessed the amplitude of emotional contagion (subjective valence and arousal) and the electromyographic activities of facial muscles (both zygomaticus major and corrugator supercilia) to estimate spontaneous facial mimicry in response to emotional expressions displayed under live or video conditions. In addition, the authors considered the role of some individual differences (empathic concern, personal and autistic traits).*

*I found the study to address a relevant research question and to be well-conducted. This is also the third revision of the manuscript. In my opinion, the authors have invested a significant amount of time and effort into addressing the comments made by the previous reviewers, leading to an improvement of the manuscript.*

*However, my main concerns are about the sample size, which is rather low (n = 50) to examine the potential moderation of several variables on different outcomes, and the fact that this study integrates data that were already published and discussed before (half of the sample includes data from the previous dataset, that is, the first publication included 23 participants and the present study only added 27 participants). Furthermore, the sample only included women. It would have been more relevant if the authors had collected a much larger sample to also include men. This can be particularly relevant because the research question includes traits that are important to both women and men.*

*Therefore, instead of acknowledging these concerns as limitations to be addressed in the future, I would recommend the authors to address them in this study and collect more data to provide more robust findings. In my view, these concerns can easily be solved by collecting a much larger sample and preferably not including the previous participants. It will also allow examining the nonsignificant two- and three-way interactions that the authors acknowledged might have been due to the low sample size.*

*With a different sample, the authors will also be able to compare some of the current findings with the previous publication, which is also important for identifying patterns that may have been missed in the previous sample. To sum up, given the importance of the research questions and the limitations also acknowledged by the authors concerns, but that can be solved with more data, I would strongly recommend that the authors consider collecting more data to strengthen the study's conclusions.*

*In conclusion, I found the research questions relevant and the methodology sound, but it is crucial to consider further data collection to provide more robust conclusions. By doing so, the study would also be able to have a greater impact among the research community.*

**Authors’ reply:** We thank the reviewer for acknowledging our effort in this manuscript.

We agree with the reviewer that, ideally, it would have been more conclusive to test up to 200 participants as suggested by the power estimation in the “Sample selection and sample size” subsection of the Limitation section. However, this isn't easy in terms of time and budget. Preparing for physiological (EMG) recordings takes time to ensure adequate data quality. For each testing session, both the model and the participant of the dyad need to be monetarily compensated. The budget issue was the main reason we stopped at a sample size of 50 for the current study.

Furthermore, previous studies suggested that dyads of sender-perceiver with different gender combinations might yield different facial mimicry patterns. It is known that females showed stronger smile mimicry than males. When involving between-gender dyads, other confounding factors, such as attractiveness, motivations to flirt, or extroverted traits, would further modulate facial mimicry's pattern and effect size. Therefore, the required sample size for different gender pairs (female sender-male perceiver, male sender-male perceiver, male sender-female perceiver) might be different (presumably even larger) to detect the three-way interaction effect of interest in our paradigm. The introduction of further possible confounding factors would also further increase the complexity of the statistical models. Therefore, we consider that the live effect in different dyadic gender combinations needs to be evaluated separately.

We have added the above information in the “Sample selection and sample size” (lines 828-830) and “Generalizability” (lines 871-876) subsections of the Limitation section.

***Reviewer #4:*** *I really appreciate the authors’ revised version of the document. The reviewer comments have been well addressed and the contents are presented more clearly. There are still a few minor concerns especially regarding the readability of the paper:*

*Abstract:*

1) *L17: Similar to L42 „moreover” (instead of “however”) seems to be more appropriate here.*
2) *L24: “Individual differences included…”: “Individual differences measures” might be clearer.*

**Authors’ reply:** We followed the reviewer’s suggestions.

3) *L25: The term “live conditions” has not been introduced. In the sentences before it was described as “image relay system to present live performances”. The usage of consistent terms would increase the comprehensibility.*

**Authors’ reply:** For consistency, we changed the term “live conditions” to “live performances”.

4) *L33: The abbreviation “AQ” should be introduced before the first use.*

**Authors’ reply:** We changed “AQ” to the full term “autism spectrum quotient.”

5) *L34ff: “…could yield valuable insights” is a bit vague. Can it be made more precise on the basis of the core results of the study? What is the main benefit/implication of the paper?*

**Authors’ reply:** To be more specific, we changed “valuable insights” to “different patterns of trait–behavior relationships” based on our findings.

Introduction:

6) *L83/84: What is meant by “this perspective”?*

**Authors’ reply:** We now specify that “the social regulator perspective also proposed that….”

7) *L174ff: Before deriving the main research questions (L182ff) there a many methodological details concerning the conducted study (e.g. details regarding the sample). It should be considered to move these methodological aspects to the method section and to focus in the last section of the introduction on the main research questions.*

**Authors’ reply:** We deleted the sentence about the three-minute conversation before the actual experiment since this information is also described in the “Paradigm and procedures” section (lines 287-289). We moved the sentence explaining why we recruited only female participants to the beginning of the “Participant” section (lines 230-231)

Materials and methods:

8) *L231: To make even more clear that IRI is your measure for trait emotional empathy you could explicitly name it in the sub-headline, for example: Trait emotional empathy: IRI.*

**Authors’ reply:** We followed the reviewer’s suggestion. We also changed the following sub-headline to “Autistic traits: AQ.”

9) *L296ff: Since the affect grid measures of valence and arousal are important variables in the analysis section (and regarding the main research questions), it could be considered to include them in the instruments section (e.g. after AQ) rather than in the paradigm and procedures section.*

**Authors’ reply:** We followed the reviewer’s suggestion and moved the information related to the Affect Grid to a subsection “Subjective experiential ratings: the Russel ‘affect grid’” after the subsection “Autistic traits: AQ.”

10) *L350ff: Why did you use both paired samples t-Test and the Wilcoxon rank test? You could mention the main reason (data quality, scale level…) or omit one of the two analyses.*

**Authors’ reply:** Because the distribution of the anger and sadness ratings did not violate the assumption of normality (anger ratings: Shapiro-Wilk W = 0.96, p = 0.39; sadness ratings: Shapiro-Wilk W = 0.96, p = 0.30), we updated this information (lines 366-367) and omitted results of the nonparametric Wilcoxon rank test.

Discussion:

11) *L636ff: The first sentence summarizes the main research question of the paper. The following sentence combines information concerning the analysis strategy and specific results (interaction emotional conditions x presentation) that has not been introduced in the sentence before. It would improve the readability of the section if the aspects were clearly separated: main research question, analysis strategy, report of the main results regarding different facets of the main research questions (in different sentences).*

**Authors’ reply:** The second sentence of the discussion was extremely long. We followed the suggestion and separated the information concerning the analysis strategy and results into separate sentences (lines 651-655).

12) *A general recommendation to improve the readability of the discussion section: Due to the complex research design, there a many different results (two-way-/three-way interactions for different dependent variables) that have to be reported. Furthermore, the various results should be clearly related to the questions/hypotheses formulated at the end of the introduction section (L182ff). It might be helpful to number each research question and then systematically refer to these questions/hypotheses in the discussion sections, for example: regarding research question 2 postulating effect b, a two-way interaction between … could be found… Overall, it should be made clear which research question is addressed by reporting results for specific (interaction) effects.*

**Authors’ reply:** We have reorganized the discussion until the section on Limitations. We first defined four research questions at the end of the introduction. In the first part of the discussion, we summarized the main results in relation to the research questions: none of the expected modulatory effects of live conditions were supported by our data. The following discussions were done in the sequence of the four research questions on relevant findings, e.g., although the presentation conditions did not modulate the relationship between autistic traits and emotional contagion/facial mimicry, presentation conditions modulated the relationship between autistic traits and smiling behavior.

13) *Significant interactions are reported at several points in the discussion but are not fully explained. For example: L641ff: interaction emotional condition x presentation, L641 does not explain the nature of the interaction; similar: L647ff, L654ff. When a significant interaction is reported, the main characteristic of the interaction should be named (e.g. difference in this condition of variable a, but not in this condition…).*

**Authors’ reply:** We added descriptions for the nature of interactions for the abovementioned points (lines 655-656, 665-668, 672-673).

14) *L657ff: After the first section of the discussion it is not entirely clear why and according to which criteria sub-headings have been chosen in the following. There are many interactions, dependent variables and research questions. It is recommended to reconsider the structuring of the results summary and interpretation within the discussion section in order to further improve the readability (see also comment 12).*

**Authors’ reply:** We have restructured the discussion to address concerns raised in comments 12 and 14. Please see our reply to comment 12 for details.
