# Supplementary material for: An investigation of the modulatory effects of empathic and autistic traits on emotional and facial motor responses during live social interactions
Source: PLoS One. 2024 Jan 9;19(1):e0290765. doi: 10.1371/journal.pone.0290765 (PMC10775989; doi:10.1371/journal.pone.0290765)
Supplement: Attachment — Submitted filename: Reply_to_Reviewers_R5.docx [file pone.0290765.s014.docx]

*Reviewer #3:*

*Point 1: Dear Authors, thank you for your response regarding the concerns I raised in my previous review. I fully understand the challenges involved in data collection, particularly when it includes physiological measurements. However, I wish to emphasize that the main focus of our scientific mission should be the reliability of the conclusions we share with the community. These aspects are influenced by the statistical power of a given study, since these estimates serve as guides, indicating the necessary sample size to reliably detect an effect, if it indeed exists.
I appreciate your acknowledgment of this principle in the "Sample selection and size" subsection of your paper. However, I encourage you to contemplate the problems of not meeting these power estimates. Conducting statistical tests with insufficient power may increase the risk of Type II errors, leading to the possible overinterpretation of nonsignificant findings related to the failure in detecting effects. If your study's intention is to test moderation effects, a larger sample size is still required based on your power estimates to the robustness of these tests. Despite the constraints you face, I believe addressing this issue is of paramount importance.

The statement in your paper, “If an effect size exists, it is likely to be very small; thus, a much larger sample size is required to detect such an effect reliably...", while acknowledging the need for more data, may not send the most constructive message to the research community. Similarly, since the primary focus of your paper are not the difficulties in data collection, the sentence, "This could be difficult for many researchers in terms of time and budget. Preparing for physiological (EMG) recordings takes time to ensure adequate data quality. For each testing session, both the model and the participant of the dyad need to be monetarily compensated.", might not be relevant to the core message of your study.

Again, the emphasis should be on the reliability of the conclusions drawn from your study. Considering these constraints, I see two potential solutions:
Reframe your conclusions to align with the statistical power available from your current sample size of 50. This would involve narrowing the scope of your statistical tests (e.g. not addressing moderations).
Alternatively, seek additional funding or resources to support further data collection, thereby enhancing the study's power.
In closing, I commend the thoughtful work you have accomplished thus far, and I firmly believe that addressing these issues will increase the value of your contribution to the field. I look forward to reviewing your revised manuscript, if necessary.*

Authors’ reply to point 1:

We considered the reviewer’s suggestions and addressed the issues as follows:

1. We included the rating and EMG data from another published fMRI study using the same design [1], thus making the sample size of the present study 94. We also included the random factor “Type” (behavioral vs. neuroimaging) when model comparisons suggested the necessity, as in the valence ratings.
2. An estimation of the required sample size for two-tailed t-tests of multiple regression coefficients with 12 predictors with a medium effect size, partial *r^2^* = 0.09, *f^2^* = 0.0989 [2,3], to achieve 80% of power using G*Power Version 3.1.9.6 suggested that a sample size of 82 was required. In addition, the sensitivity analysis for two-tailed t-tests of multiple regression coefficients with 12 predictors and a sample size 94 showed that the present study has 80% of power for a minimum detectable effect size of *f^2^* = 0.0855, *r^2^* = 0.0788.
3. Due to the inversed relationship between the observed power and the *p-values*, a very small or null effect would invariably result in a large *p-value* and a low observed power [4,5]. Thus, making inferences based on the effect size under such circumstances is more informative. While it still requires a large sample size to make accurate estimations of small effect sizes, the required sample size for precise estimation is smaller than the required sample size to achieve high power. For a small effect size of *r^2^* = 0.01, only a sample size of 61 is required to achieve the point of stability with 80% confidence for the effect size estimation, while a sample size of 394 is required to observe such an effect with an alpha of 0.05 [6]. Therefore, the sample size of the present study is also more than sufficient to accurately estimate small effect sizes and make inferences based on that.
4. We reduced the number of fixed-effect predictors by excluding IRIPD and associated interaction effects, making the number of fixed-effect predictors 12. IRIPD was significantly correlated with IRIEC and AQ in the expanded sample and easily resulted in overfitting (singular fit) or failure to converge in model estimation.
5. We also simplified our approach to model diagnostics. We iteratively increased model complexity using the complete dataset, and ran robust estimations of the final model [7] to check the significant result patterns when underweighting influential data points. When the result patterns of the robust and non-robust estimations are identical, we report and discuss the result details of the non-robust estimation of the complete dataset, keeping as much data and participants in the analysis as possible to retain statistical power. In the case of CS responses, the result patterns of the robust and non-robust estimations were not identical; we performed model diagnostics as before to exclude influential data points, which resulted in the same result pattern as the robust estimation of the complete dataset. We report and discuss the non-robust result details with model diagnostics for CS responses. Results of robust estimation were included in the supplementary tables (S1-S4 Tables).

We observed two-way interactions between emotional and presentation conditions in all four measurements (valence and arousal ratings and ZM and CS responses). We also observed two-way interactions between IRIEC and emotional conditions in valence and arousal ratings, and CS responses. We still observed no three-way interactions among traits, emotional and presentation conditions. The effect size and confidence interval of effect size estimates suggested that the effects of three-way interactions are indeed small. As the results are more straightforward, the Results and Discussion sections were also much reduced, including paragraphs that the reviewer considered irrelevant.

*Reviewer #4: Thank you very much for the opportunity to review this interesting and relevant paper again.
The authors did a very good job with the revision. From my point of view, all concerns were thoroughly and adequately addressed. The structure of several sections (theory, discussion) is much clearer, and the description of the interactions is much more precise now.
Of course, a larger sample size would provide more robust insights. Considering the high effort of collecting physiological data, I think the current sample size is justifiable. Limitations based on the sample size were sufficiently pointed out in the revised version of the document.*

Authors’ reply: We thank the reviewer for the comments.

**Bibliography**

1.	Hsu C-T, Sato W, Kochiyama T, Nakai R, Asano K, Abe N, et al. Enhanced mirror neuron network activity and effective connectivity during live interaction among female subjects. NeuroImage. 2022;263: 1–19. doi:10.1016/j.neuroimage.2022.119655

2.	Cohen J. Statistical Power Analysis for the Behavioral Sciences. 2nd ed. New York: Routledge; 1988. doi:10.4324/9780203771587

3.	Gignac GE, Szodorai ET. Effect size guidelines for individual differences researchers. Pers Individ Differ. 2016;102: 74–78. doi:10.1016/j.paid.2016.06.069

4.	Hoenig JM, Heisey DM. The Abuse of Power: The Pervasive Fallacy of Power Calculations for Data Analysis. The American Statistician. 2001;55: 19–24. doi:10.1198/000313001300339897

5.	Greenland S. Nonsignificance Plus High Power Does Not Imply Support for the Null Over the Alternative. Annals of Epidemiology. 2012;22: 364–368. doi:10.1016/j.annepidem.2012.02.007

6.	Lakens D, Evers ERK. Sailing From the Seas of Chaos Into the Corridor of Stability: Practical Recommendations to Increase the Informational Value of Studies. Perspect Psychol Sci. 2014;9: 278–292. doi:10.1177/1745691614528520

7.	Koller M. **robustlmm** : An *R* Package for Robust Estimation of Linear Mixed-Effects Models. J Stat Soft. 2016;75. doi:10.18637/jss.v075.i06
