# Supplementary material for: An investigation of the modulatory effects of empathic and autistic traits on emotional and facial motor responses during live social interactions
Source: PLoS One. 2024 Jan 9;19(1):e0290765. doi: 10.1371/journal.pone.0290765 (PMC10775989; doi:10.1371/journal.pone.0290765)
Supplement: Attachment — Submitted filename: Reply_to_Reviewers_R6.docx [file pone.0290765.s015.docx]

1. *First, I appreciated your careful attention to the comments about statistical power. While increasing your sample size by including data from a related study certainly addressed the reviewer’s concern about power, I worry that it also adds an additional confound that may introduce problems if the experimental paradigms introduce unknown systematic differences. For instance, if participants are laying in a MRI magnet, this may alter their attention or response to stimuli in a number of ways. They may be more anxious overall, which could alter their attention to positive or negative stimuli. Or the simple act of laying down may impact the magnitude of muscle activation. Of course, there is no way to know for sure, which is the danger. That said, you may want to keep this larger sample to maintain power. In this case, you might consider ways to verify that they data collection procedures do not impact your models. This could be done by comparing the two data collection methods directly, conducting a sensitivity analysis, or including the data collection method as a factor in the model. However, I think it is also acceptable to just report on your original sample (N=50) as these tests appeared to be sufficiently powered to address your main findings. If you choose to report on your original sample, however, I think your simplified models (as noted in points 4 and 5 of your response letter) are sufficient to address any concerns regarding your analytic approach. That is, you will need to continue to use these refined models if you choose to go back to your original sample.*

Authors’ reply:

We thank the editor for the suggestion. According to the recommendations, we made the following changes to our analysis:

1. We add the random intercept over the random factor “Type,” wherever this did not cause a singular fit. We already included the random intercept over “Type” in the valence rating analysis in the previous revision. We also include the random intercept over “Type” in the ZM and CS response analysis. Including random slopes over “Type,” or modeling the random factor as “subject nested in Type” also invariably caused singular fits, so only random intercept could be added. However, adding the random intercept over “Type” in the arousal rating analysis invariably caused singular fits, so the random factor Type could not be added for the arousal ratings (Line 550). Information about model comparisons between models, including and not including random intercept over “Type,” was also added to the Results section (Lines 490-492, 594-596, 622-624). The significant patterns were the same as in the last revision.
2. We also included robust data estimations from 50 participants in the behavioral study of the same models as with 94 participants in the supplementary tables S2, S4, S6, and S8. We see a few interactions significant with 94 participants, not significant with 50 participants (Emotion X Presentation in valence and arousal ratings; IRIEC X Emotion in CS responses), which we attributed to the effect of statistical power. No further discussion was added. We also added information about a sensitivity analysis for 50 participants in the Participant section of the Methods, showing that 50 is apt for detecting medium effect sizes (Lines 244-247).
2. *Second, and related to the issue of power, you added text regarding a power analysis. While this seems to be an effort to convince the reader that your statistical tests are sufficiently powered, I worry that it would function as a null power test for many readers. As you note, post hoc power studies are inherently flawed, and therefore, I don't think this language is needed. Please remove this language.*

Authors’ reply:

We aimed to justify the sample size we offered in this study by showing that 94 is apt for detecting a small-to-medium effect size, and the effect size estimation for a small effect size is reliable. As the editor suggested, sensitivity analyses are sufficient for this purpose, so we have removed other means of sample size estimation in the Participant section of the Methods (Lines 236-247).
